# Design Refinement of Catalytic System for Scale-Up Mild Nitrogen Photo-Fixation

**DOI:** 10.1007/s40820-025-01695-3

**Published:** 2025-03-12

**Authors:** Xiao Hu Wang, Bin Wu, Yongfa Zhu, Dingsheng Wang, Nian Bing Li, Zhichuan J. Xu, Hong Qun Luo

**Affiliations:** 1 School of Chemistry and Chemical Engineering, Southwest University, Chongqing, 400715 People’s Republic of China; 2 School of Materials Science and Engineering, Nanyang Technological University, 50 Nanyang Avenue, Singapore, 639798 Singapore; 3 Department of Chemistry, Tsinghua University, Beijing, 100084 People’s Republic of China

**Keywords:** Scale-up, Mild nitrogen photo-fixation, Design refinements, Catalyst system, Environmental sustainability

## Abstract

Ammonia and nitric acid, versatile industrial feedstocks, and burgeoning clean energy vectors hold immense promise for sustainable development. However, Haber–Bosch and Ostwald processes, which generates carbon dioxide as massive by-product, contribute to greenhouse effects and pose environmental challenges. Thus, the pursuit of nitrogen fixation through carbon–neutral pathways under benign conditions is a frontier of scientific topics, with the harnessing of solar energy emerging as an enticing and viable option. This review delves into the refinement strategies for scale-up mild photocatalytic nitrogen fixation, fields ripe with potential for innovation. The narrative is centered on enhancing the intrinsic capabilities of catalysts to surmount current efficiency barriers. Key focus areas include the in-depth exploration of fundamental mechanisms underpinning photocatalytic procedures, rational element selection, and functional planning, state-of-the-art experimental protocols for understanding photo-fixation processes, valid photocatalytic activity evaluation, and the rational design of catalysts. Furthermore, the review offers a suite of forward-looking recommendations aimed at propelling the advancement of mild nitrogen photo-fixation. It scrutinizes the existing challenges and prospects within this burgeoning domain, aspiring to equip researchers with insightful perspectives that can catalyze the evolution of cutting-edge nitrogen fixation methodologies and steer the development of next-generation photocatalytic systems.

## Highlights

- The review provides a brief overview of basic mechanisms, element selections, activity confirmation, and experimental protocols of photocatalytic nitrogen fixation under mild conditions.
- The review details strategies for scale-up photocatalysts in nitrogen fixation, emphasizing defect engineering, facet optimization, heteroatom doping, single-atom site creation, and composite synthesis.
- The review emphasizes the importance of environmental assessment for photocatalyst lifecycle sustainability in mild nitrogen fixation for the future.

## Graphical abstract

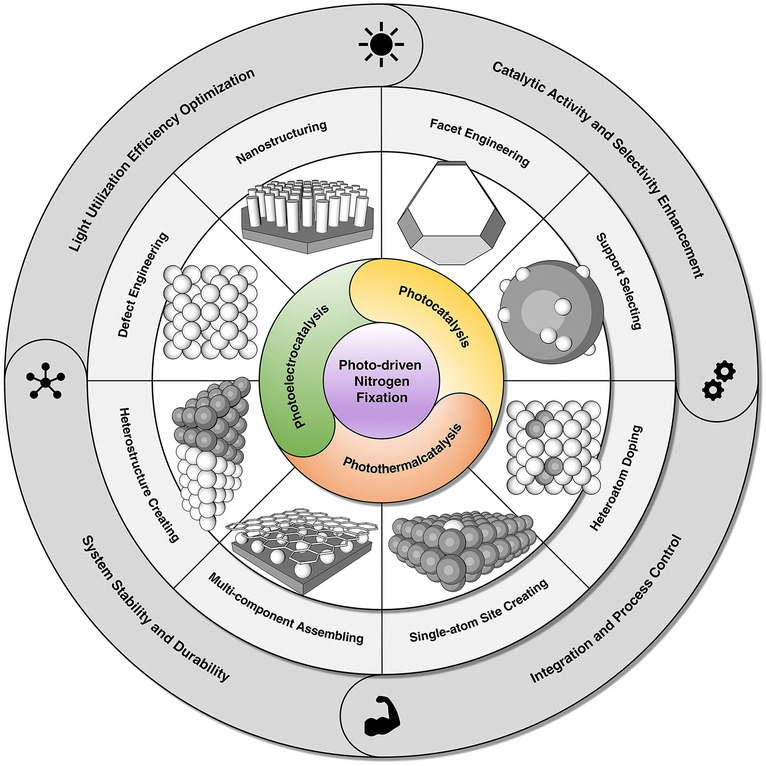

## Introduction

In the critical processes of chemical industry and agricultural production, nitrogen fixation is of paramount importance. The conversion of atmospheric nitrogen (N_2_) into ammonia (NH_3_) and nitric acid (HNO_3_) is a cornerstone for global food security and the development of the chemical industry [1–4]. The traditional Haber–Bosch (H–B) and Ostwald processes, while pivotal in the history of industrial chemistry, exert significant environmental pressure due to their high energy consumption and substantial greenhouse gas emissions [5]. Traditional nitrogen fixation methods rely on high temperatures and pressures, along with catalysts. For instance, the H–B process requires temperatures of 300–400 °C and pressures of 200 atm, which are energy-intensive and limit application flexibility. The produced NH_3_ often contains impurities such as methane and hydrogen (H_2_), reducing the purity and efficiency of the ammoxidation process [6, 7].

To address these issues, researchers have embarked on an innovative exploration of low-energy nitrogen fixation. During this exploration, various alternative approaches have been investigated. However, it is crucial to note that even when considering the use of green hydrogen in the Haber–Bosch process, it still presents significant limitations. The high-temperature and high-pressure requirements of the Haber–Bosch process lead to inevitable energy losses during operation, despite the utilization of a supposedly sustainable energy source. The infrastructure needed to maintain such extreme conditions is costly and complex. In contrast, photocatalytic nitrogen fixation holds the potential to not only avoid these issues but also offers a more decentralized and flexible solution [8, 9]. This is because it can operate under mild conditions, making it adaptable to a wider range of scenarios and potentially reducing the overall environmental footprint. From refining traditional processes to adopting emerging technologies, significant improvements in energy efficiency have been achieved, propelling the green transformation of the chemical industry [10, 11]. Among these advancements, the development of photocatalysis technology has brought breakthroughs to low-energy photocatalytic nitrogen fixation [12, 13]. Photocatalysts under mild conditions utilize solar energy to achieve nitrogen reduction, opening new avenues for nitrogen fixation [14–18].

However, the industrial application of photocatalytic nitrogen fixation technology faces numerous challenges, with the structural design and optimization of photocatalysts being crucial [19–21]. Traditional photocatalysts suffer from low-light absorption efficiency and high recombination rates of photogenerated electron–hole pairs, limiting their performance [22, 23]. Structural engineering has emerged as a key means to enhance performance. The photophysical properties of photocatalysts are essential for their nitrogen fixation capabilities. Ideal photocatalysts should efficiently absorb light and generate electron–hole pairs, with their lifetime, migration rate, and recombination rate being pivotal [14, 24, 25]. By adjusting the material's band structure and electronic configuration, these properties can be optimized to enhance catalyst activity [26, 27]. For example, nanoscale structural design can significantly improve light absorption and charge-separation efficiency. Transition metal dichalcogenides (e.g., MoS_2_ [28]) and carbon-based materials (e.g., g-C_3_N_4_ [29, 30]) have demonstrated outstanding performance in photocatalytic nitrogen fixation due to their unique electronic and optical properties [31, 32]. Surface modification and heterostructure construction are also effective strategies for optimizing photocatalysts [33–38].

Despite progress in the study of photocatalytic nitrogen fixation catalysts, achieving industrialization remains challenging. The large-scale production of photocatalysts is a critical step from laboratory innovation to industrial application [39]. Many high-performance photocatalysts are still confined to research laboratories, hindered by cost, process complexity, and repeatability [40, 41]. Developing new, low-cost, and scalable synthesis methods and optimizing production processes are essential to enhance their industrial viability [42]. Chemical stability is indispensable for the industrial application of photocatalysts. They must maintain structural and chemical integrity under sunlight and non-ideal reaction conditions to avoid deactivation. Introducing doping elements or constructing protective layers can enhance stability and extend operational lifespans. Before widespread adoption, a comprehensive environmental impact assessment is necessary. This includes evaluating the potential environmental effects throughout the catalyst's lifecycle, from production to disposal. Through lifecycle assessment and environmental risk assessment, potential hazards can be identified and mitigated, ensuring the sustainability of the catalyst in practical applications [43, 44].

A deeper analysis of the challenges faced by photocatalysts in practical applications can help clarify research bottlenecks and guide future directions. Scaling-up photocatalytic nitrogen fixation catalysts requires learning from successful cases in similar applications, including equipment design, adaptation strategies, performance measurement techniques, and the establishment of performance metrics [45]. By dissecting these success stories, a better understanding of the principles and practices of photocatalyst structural engineering can be achieved, facilitating the transition from laboratory to industrial application and achieving comprehensive improvements in cost, activity, stability, and environmental friendliness, laying the foundation for sustainable nitrogen fixation [46, 47].

**Fig. 1:**
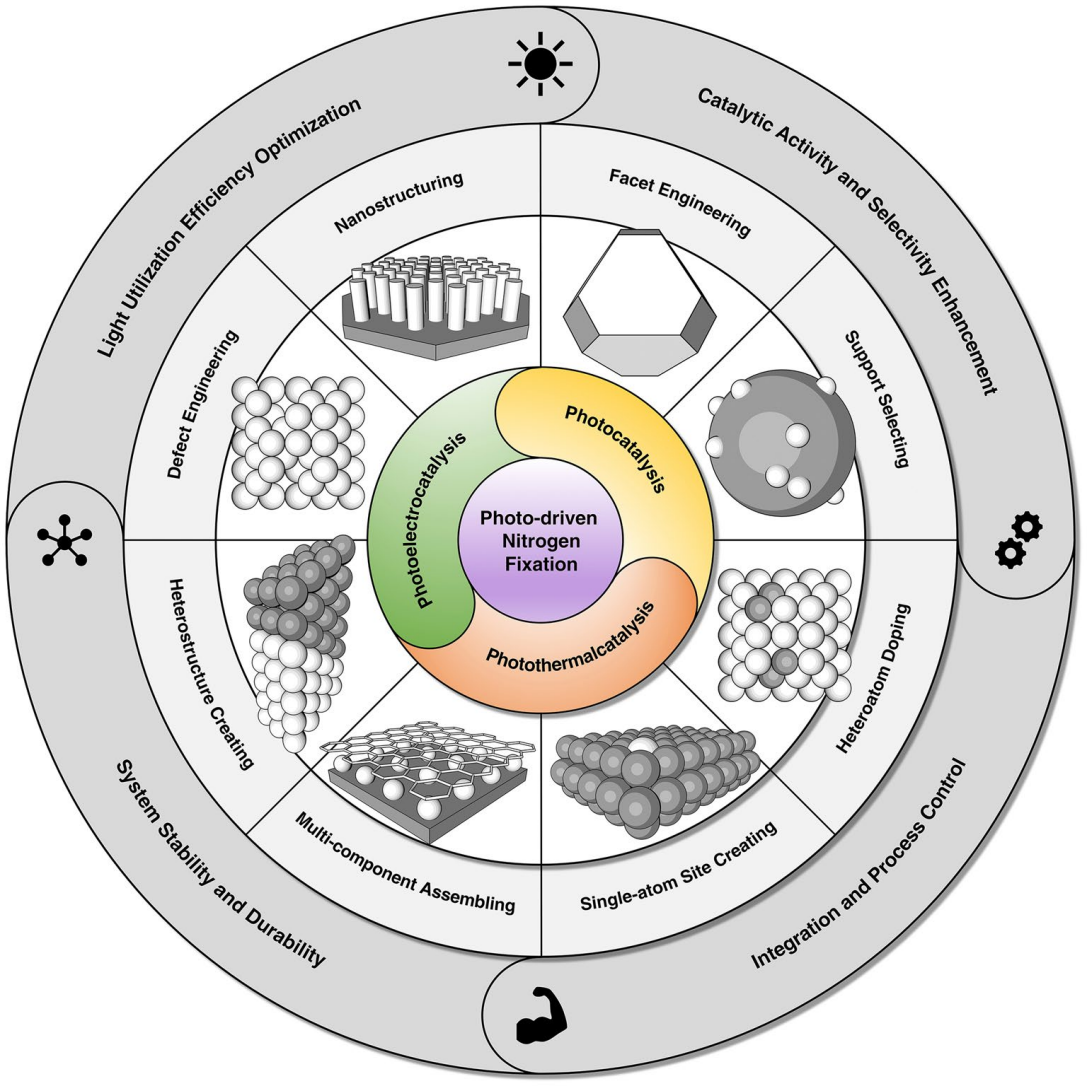
Schematic illustration of the design refinement of catalytic system for scale-up mild photocatalytic nitrogen fixation

In this review, we systematically present the design improvements and achievements of photocatalytic nitrogen fixation catalytic systems under mild conditions, from laboratory to scaled-up applications (Fig. 1). We begin by analyzing the fundamental principles, elements, and experimental protocols of the catalytic system to provide a theoretical basis for research. We then focus on strategies to enhance catalyst performance through structural engineering, considering cost, stability, and environmental friendliness. Future research directions are envisioned, exploring the use of artificial intelligence and machine learning to optimize photocatalyst structures, develop new synthesis techniques, and explore potential applications in other fields [48, 49]. Finally, we outline the current challenges and future prospects of rational design for photocatalytic nitrogen fixation systems, offering a comprehensive and insightful perspective on the research and application of energy-saving nitrogen fixation, and supporting the industrial-scale application of photocatalytic nitrogen fixation technology and the green and sustainable development of the chemical industry.

## Theoretical Insights into Photocatalytic Nitrogen Fixation

The scrutiny of reaction intermediates and transition states throughout the photocatalytic nitrogen fixation process has garnered considerable research interest. An integrative approach encompassing in situ spectroscopic techniques coupled with theoretical calculations is instrumental in revealing the critical reaction intermediates and energetic landscapes, thereby offering prescriptive guidance for catalyst optimization. In the purview of photocatalytic systems, the generation of electron–hole pairs subsequent to light irradiation is deemed indispensable for the start of nitrogen fixation process. The efficacious separation and harnessing of photogenerated charge carriers are paramount to augmenting nitrogen fixation efficacy. Consequently, the band structure dynamics of photocatalysts and the kinetics of photogenerated charge carriers have emerged as focal points within theoretical research domains. The theoretical research on photocatalytic nitrogen oxidation reactions is a multifaceted and intricate endeavor, intersecting various disciplines such as catalyst surface chemistry, reaction kinetics, thermodynamics, and materials engineering. Rigorous theoretical dissection is imperative for underpinning the development of efficacious, eco-friendly, and sustainable nitrogen fixation catalytic systems, thereby propelling forward the frontiers of nitrogen utilization and transformation.

### Nitrogen Reduction Reaction

The direct transformation of nitrogen to ammonia, synonymous with the natural process of nitrogen reduction reaction (NRR), is recognized for its significance within the sustainable energy production, environmental protection, and advancement of chemical industry.

**Fig. 2:**
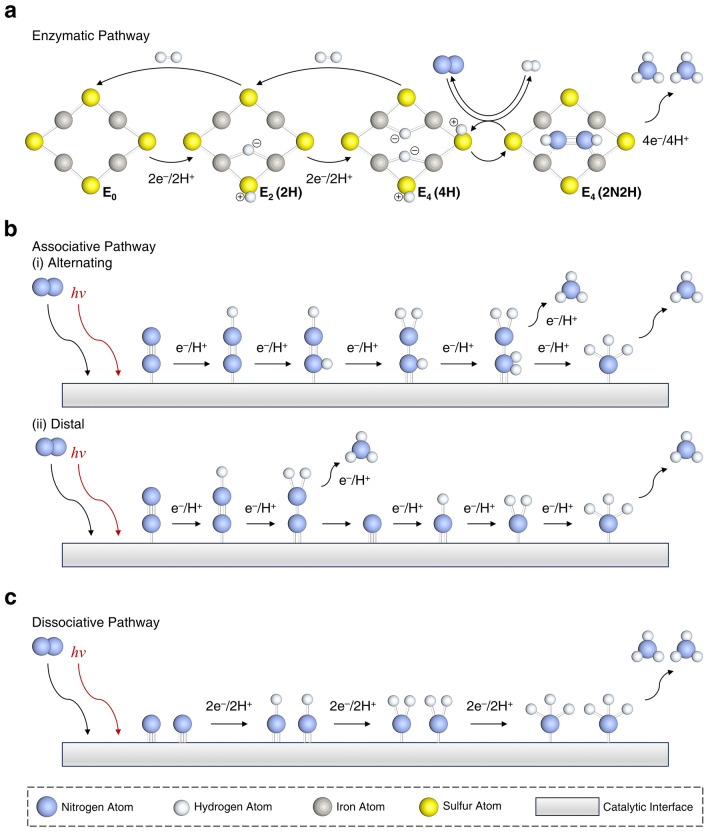
**a** Proposed hydride reduction procedure and the NRR at the active site that occurs in most of nitrogenases. **b** N≡N triple bond in N_2_ typically remains intact until NH_3_ formation. N_2_ adsorbs onto catalysts in two configurations. **c** N_2_ molecules first dissociate, cleaving the N≡N bond to produce adsorbed N species, which subsequently hydrogenate stepwise to form NH_3_

In nature, the process of enzymatic nitrogen fixation, electron transfer begins with nitrogen-fixing microorganisms generating electrons and protons through respiration, fermentation, or photosynthesis, reducing nicotinamide adenine dinucleotide (NAD) or nicotinamide adenine dinucleotide phosphate (NADP) to nicotinamide adenine dinucleotide-hydrogen (NADH) or nicotinamide adenine dinucleotide phosphate-hydrogen (NADPH). These reduced electron carriers further reduce ferredoxins or flavodoxins, and these reduced electron carriers transfer electrons to the (4Fe − 4S)^1+^ in the iron protein of nitrogenase, reducing it to (4Fe − 4S)^2+^. Subsequently, the iron protein transfers electrons to the iron–sulfur (Fe–S) cluster of molybdenum–iron (MoFe) protein, which then transfers electrons to the MoFe cofactor. Concurrently, adenosine triphosphate (ATP) combines with magnesium (Mg) ions to form Mg-ATP complexes, which bind with the Fe protein, hydrolyzing two Mg-ATP molecules per electron transferred, providing energy for the entire process. Under the catalysis of the MoFe cofactor in MoFe protein, nitrogen gas accepts electrons and protons from the aforementioned electron transfer process and is gradually reduced according to the reaction:

1 $${\text{N}}_{2}+8{\text{H}}^{+}+8{\text{e}}^{-}+16\text{ATP}\to 2{\text{NH}}_{3}+{\text{H}}_{2}+16\text{ADP}/\text{Pi}$$

with 2 protons and electrons used to generate H_2_, completing the substrate reduction process. This series of complex and exquisite reactions constitute the key steps in enzymatic nitrogen fixation (Fig. 2a). In certain special circumstances, such as when Mo is scarce in the environment, vanadium (V) plays a crucial role. It is present in the FeV-co structure of vanadium nitrogenase, functioning similarly to Mo as a component of the active site. This enables microorganisms to synthesize vanadium nitrogenase, which can bind and reduce nitrogen gas. However, there are differences between V nitrogenase and MoFe nitrogenase in terms of substrate affinity and electron transfer efficiency, among other aspects of the reaction mechanism.

Similarly, the partial process of nitrogen reduction is widely considered as follows:

2 $${\text{N}}_{2}+6{\text{H}}^{+}+6{\text{e}}^{-}\to 2{\text{NH}}_{3}, { E}^{0}=-0.148 \text{V versus RHE}$$

And, the reaction starts from the following step:

3 $${\text{N}}_{2}+{\text{H}}^{+}+{\text{e}}^{-}\to {\text{N}}_{2}\text{H}, { E}^{0}\approx -3.2 \text{V versus RHE}$$

The first step in nitrogen reduction is the formation of N_2_H* species based on the proton-coupled electron transfer (PCET) mechanism. Unfortunately, this step requires a very negative potential to activate (*E*^0^ ≈ − 3.2 V versus RHE), which means that the first step to open the N≡N triple bond is tremendously difficult [50]. Afterward, further proton transfer can get two possible intermediates, including diazene (N_2_H_2_) and hydrazine (N_2_H_4_). The above two possible reaction processes can be expressed as follows [51]:

4 $${\text{N}}_{2}+{2\text{H}}^{+}+{2\text{e}}^{-}\to {\text{N}}_{2}{\text{H}}_{2},{ E}^{0}\approx -1.1 \text{V versus RHE}$$

5 $${\text{N}}_{2}+{4\text{H}}^{+}+{4\text{e}}^{-}\to {\text{N}}_{2}{\text{H}}_{4}, {E}^{0}\approx -0.36 \text{V versus RHE}$$

6 $${\text{N}}_{2}+6{\text{H}}_{2}\text{O}+{6\text{e}}^{-}\to {\text{N}}_{2}{\text{H}}_{6}+6{\text{OH}}^{-},{E}^{0}\approx -0.36\text{V versus RHE }\left(\text{when pH}=14\right)$$

There are indications that the dissociation of NH_2_* is the last step of the NRR. The decisive steps in NRR are the generation of high-energy intermediates by N≡N triple bond cleavage. Because there are numerous reaction steps in NRR and corresponding products are complex, there is no absolute unified theory to explain the reaction mechanism (Fig. 2a).

The current mainstream recognized reaction mechanism includes two types: associative mechanism and dissociative mechanism [52]. The associative mechanism includes two approaches: a distal pathway and an alternating pathway (Fig. 2b). The distal pathway proposes that the N_2_ molecules are continuously hydrogenated until the first NH_3_ molecule is released. After the release of the first NH_3_ molecule, another N atom bound to the surface of the catalyst will hydrogenate to form a second NH_3_ molecule. Differently, the alternative pathway proposes that the hydrogenation process will occur sequentially on two N atoms of N_2_ molecule, and then, one molecule of NH_3_ obtained by hydrogenation will break the N≡N triple bond and release. After the release of the first NH_3_ molecule, the second is followed. While the dissociative mechanism proposes that the N≡N triple bond of the N_2_ molecule is firstly broken after the N_2_ molecule bounded on the surface of the catalyst. Then, two N atoms independently undergo a hydrogenation process and eventually form two NH_3_ molecules. The above mechanisms occur in two cases, respectively. Initially, the associative mechanism is generally accepted to occur in biological nitrogen fixation in nature [53, 54]. Additionally, artificial NH_3_ synthesis based on the H–B process follows the dissociative mechanism (Fig. 2c) [55]. The catalytic mechanism needs to be clarified before using improved synthetic techniques. Calculations and experiments show that the catalytic pathway of NRR in aqueous media varies with catalytic systems and catalysts [56, 57].

**Fig. 3:**
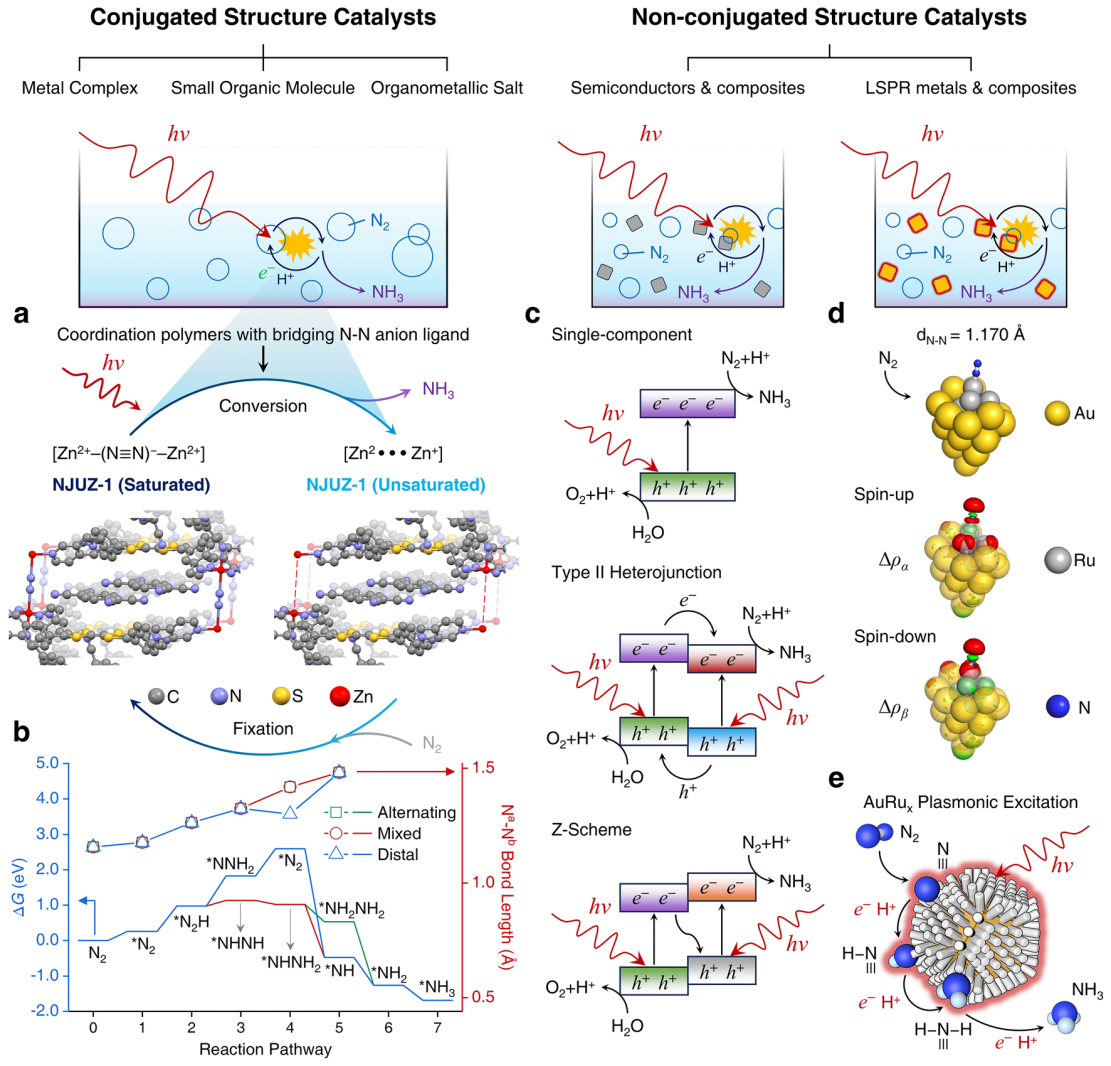
**a** Scheme of cycling process of NJUZ-1 during the photocatalytic NRR process. **b** Variation of the calculated free energies (Δ*G*) and N^a^–N^b^ bond lengths in each step of different possible reaction pathways. **c** Scheme of photocatalytic NRR processes of single-component, Type-II heterojunction, and Z-scheme semiconductor-based catalysts. **d** Optimized structures of N_2_ adsorbed on Ag_22_Ru_6_ cluster, the electron density difference for* α* (spin-up) and *β* (spin-down) is normalized, with red and green colors represent an increase and decrease in electron density, respectively. **e** AuRu core-antenna structures absorb light broadly and possess active sites that, under room temperature, pure water, and 2 bar pressure, leverage plasmonic fields and hot electrons to reduce N_2_ through dissociative pathway, producing NH_3_ swiftly without sacrificial reagents. Reproduced with permission [67]. Copyright 2019, American Chemical Society

The theoretical exploration of electrocatalytic NRR under benign conditions has been articulated by several researchers across various platforms [58–65]. However, comprehensive theoretical studies and syntheses on photocatalytic NRR remain rather sparse. Proposed mechanisms for NRR mediated by different photocatalysts can be broadly categorized based on the presence or absence of conjugated structures. Photocatalysts endowed with conjugated structures, upon photon irradiation, facilitate the transition of electrons from the highest occupied molecular orbital (HOMO) to the lowest unoccupied molecular orbital (LUMO), thereby generating photoinduced electrons and holes. A case in point is the work by Jin et al., where the [Zn^2+^–(N≡N)^−^–Zn^2+^] site in NJUZ-1 serves as the active center; its unsaturated and variable-valence metal core provides vacant sites and electron transfer to activate dinitrogen species, with the donor–acceptor–donor cavity being instrumental for light harvesting and perpetuating the catalytic cycle [66]. Upon photoexcitation, TTF-TCNQ absorbs photon energy, prompting the separation of electron–hole pairs, with electrons migrating to the active site to activate dinitrogen anions. Subsequently, proton-coupled electron transfer (PCET) leads to the formation of *N_2_H intermediates, which undergo multiple hydrogenation steps to form *NHNH, *NHNH_2_, and other intermediates, culminating in NH_3_ production, with an elongation of the N–N bond length, and NJUZ-1 acting as an electron reservoir. Concurrently, the [Zn^2+^–(N≡N)^−^–Zn^2+^]site transforms into [Zn^2+^···Zn^+^] intermediates, regenerating the catalyst through external dinitrogen exchange cycles, akin to the Mars–van Krevelen mechanism, with TCNQ stabilizing the crystal structure to sustain the cycle (Fig. 3a, b). In the case of semiconductor photocatalysts, when the photon energy exceeds or matches the semiconductor's bandgap, electrons from the valence band (VB) absorb photon energy and transition to the conduction band (CB), generating photogenerated electrons and holes. This is followed by charge separation and transfer of photogenerated charge carriers, with electrons and holes migrating to the catalyst's bulk or superficial active sites, where they engage in reduction reactions with adsorbed N_2_, adhering to a associative pathway (Fig. 3c). Moreover, certain noble metal nanoparticles, such as gold (Au), silver (Ag), and platinum (Pt), exhibit strong absorption of photon energy when the frequency of incident photons aligns with the material's overall vibrational frequency, leading to localized surface plasmon resonance (LSPR) and the consequent enhancement of electromagnetic fields, which promotes the excitation and reaction of surrounding molecules. As demonstrated by Xiong et al., a hybrid metal catalyst with an Au core and Ru antennae provides an apt platform for energy transfer from plasmonic Auto proximal Ru catalytic sites, subsequently injecting it into adsorbed N_2_ (Fig. 3d) [67]. Notably, changes in electron density predominantly occur on the surface of N_2_ and Au_22_Ru_6_ clusters, with N_2_ acquiring electrons that may localize in the antibonding orbitals of N − N, leading to the elongation of the N − N bond. This suggests the formation of a highly hybridized system between the N_2_ molecule and the plasmonic catalyst, permitting the reduction of N_2_ through a dissociative pathway [68].

Drawing inspiration from biological nitrogen fixation, the half reaction of the photocatalytic NRR process can be articulated as follows:

7 $${\text{N}}_{2}+{(6+2\text{x})\text{H}}^{+}+{(6+2\text{x})\text{e}}^{-}+\text{n}hv\to 2{\text{NH}}_{3}+{\text{xH}}_{2}$$

or

8 $${2\text{N}}_{2}+{(6+2\text{x})\text{H}}_{2}\text{O}+\text{n}hv\to 4{\text{NH}}_{3}+{(3+\text{x})\text{O}}_{2}+{\text{xH}}_{2}$$

The distinction from biological nitrogen fixation lies in the substitution of the 16 ATP units in the equation with photon energy. The theoretical potential required for photocatalytic NRR is akin to that of the hydrogen evolution reaction (HER); hence, competition between NRR and HER may be inevitable. From a production scale-up perspective, semiconductor photocatalysts are undoubtedly the most promising, with materials satisfying NRR demands necessitating a sufficiently negative CB potential for the following reasons:

1. Starting from the nature of the electron donor, it is essential to ensure that the conduction band potential is sufficiently low to initiate the overpotential required for the nitrogen reduction reaction (NRR). The characteristics of the electron donor, such as redox potential and electron-donating ability, directly affect its ability to provide the initial driving force for NRR, enabling the reaction to overcome the initial energy barrier and initiate the conversion of nitrogen molecules to ammonia.
2. By integrating the control of external reaction conditions, the conduction band potential can be sufficiently lowered to overcome the energy barriers associated with chemical bond dissociation. External conditions such as reaction temperature, pressure, and reactant concentration influence the energy state of the system. Together with the low conduction band potential, these conditions facilitate the dissociation of strong chemical bonds like the N–N triple bond, creating conditions for subsequent reaction steps and ensuring the continuous progression of the reaction.
3. Focusing on changes in the system's microscopic structure, it is crucial to maintain a sufficiently low conduction band potential to compensate for energy losses during electron migration. Changes in the microscopic structure of the system, such as the crystal structure, defects, and interfaces of the catalyst, not only affect the pathways and rates of electron migration but also require a sufficiently low conduction band potential to offset energy dissipation during electron transport. This ensures the effective flow of electrons within the reaction system, guaranteeing the successful completion of the nitrogen reduction reaction.

Consequently, enhancing NRR performance primarily hinges on selecting materials with high intrinsic capabilities and refining other components of the catalytic system. Moreover, the step prioritization and reaction pathways of photocatalytic NRR are intricately linked to the catalyst type and the subtle electronic structure of the active surface. The convergence of theoretical computations and experimental investigations will be instrumental in elucidating the reaction mechanisms of photocatalytic NRR with precision.

### Nitrogen Oxidation Reaction

**Fig. 4:**
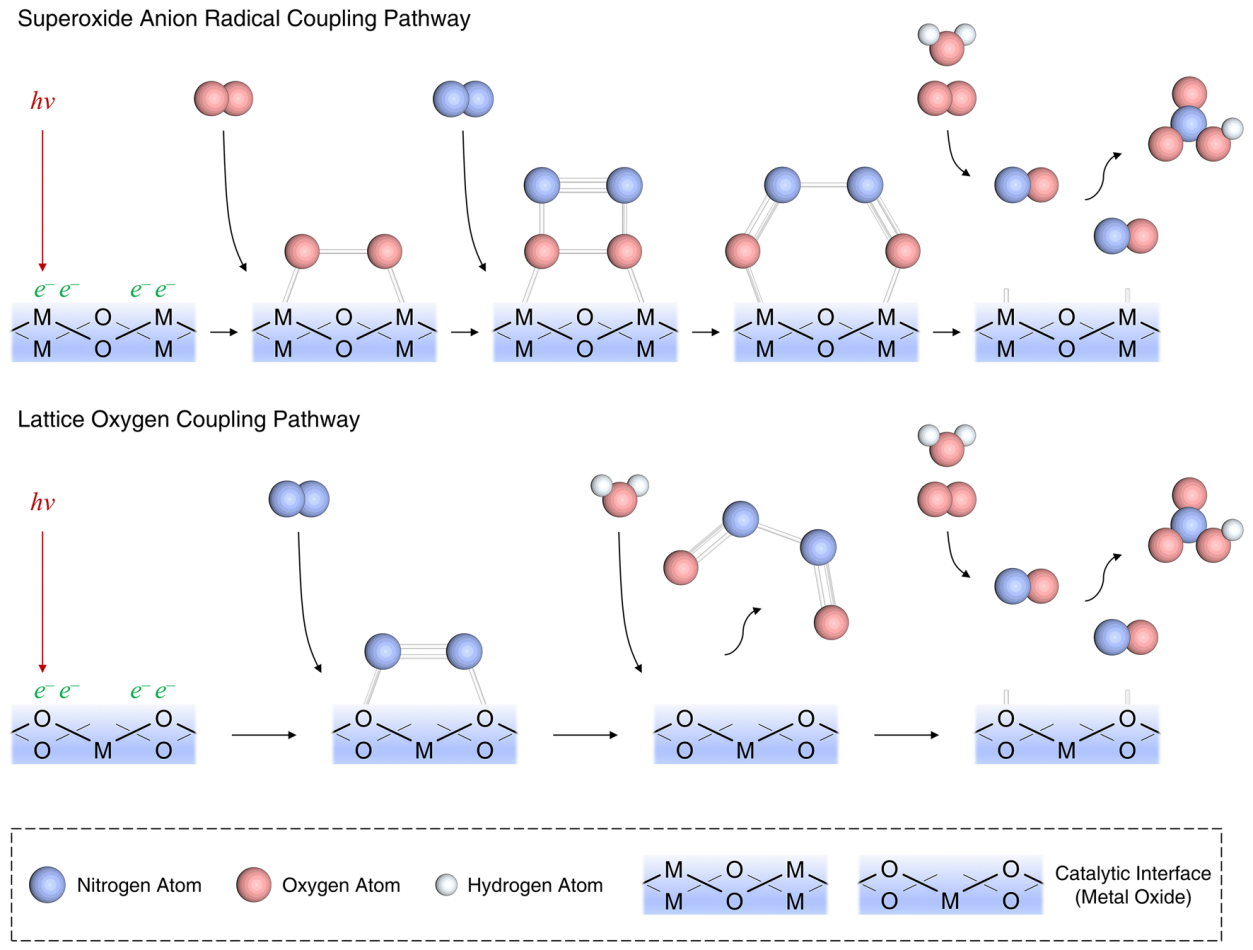
Schematic illustration of the suggest mechanisms of photocatalytic NOR process

Although the natural world encompasses an indirect mechanism for the conversion of N_2_ to HNO_3_, this process is characterized by its slow pace and intricate biogeochemical cycling, diverging from a direct oxidative reaction. In contemporary industrial applications, the predominant method for the production of HNO_3_ is the Ostwald process, which necessitates conditions of elevated temperature and pressure, thereby distinguishing itself from the natural nitrogen cycle [9, 69]. The direct photocatalytic conversion of N_2_ to HNO_3_, facilitated by solar energy, represents an environmentally advantageous alternative to traditional multi-stage synthesis methodologies. However, the underlying mechanisms pertaining to the activation and transformation of the N_2_ molecule's inert chemical bonds remain obscure and are yet to be comprehensively delineated within the scientific community. The theoretical investigation into photocatalytic nitrogen oxidation reactions (NOR) is fundamentally centered on elucidating the mechanisms of nitrogen molecule adsorption, activation, and subsequent oxidation on catalytic surfaces (Fig. 4). This sequence of events is equally pivotal for nitrogen fixation and the synthesis of nitrogenous compounds [69].

Initially, the molecular adsorption and activation of N_2_ on the catalyst surface constitute the nascent phase of NOR. Given the inherent stability conferred by the N≡N triple bond, nitrogen molecules pose a significant challenge in terms of activation on catalytic surfaces. Empirical data suggest that the presence of oxygen vacancies at the catalyst surface can facilitate the adsorption and activation of N_2_ molecules, consequently diminishing the activation energy threshold for the reaction [70].

Subsequently, the nitrogen atoms, once activated, engage in oxidative coupling with oxygen species, culminating in the formation of nitric oxide (NO) or other nitrogen oxides. This cascade of events may entail a series of electron and proton transfer processes. For redox potential of N_2_/NO_2_, it is generally 1.06 V versus SHE, but it is noted that this value can vary slightly depending on reaction conditions. When it comes to NO_2_/HNO_3_, the redox potential should be the 0.93 V versus SHE. Theoretical frameworks, exemplified by density functional theory (DFT) computations, are utilized to delineate these reaction pathways and prognosticate the reaction kinetics and thermodynamic properties across a spectrum of conditions. One possible reaction mechanism is as follows [71]:

9 $$\text{MO}+\text{n}hv\to {h}^{+}(\text{MO})+{e}^{-}(\text{MO})$$

10 $${\text{O}}_{2}+{e}^{-}\to \cdot {\text{O}}_{2}^{-}$$

11 $${\text{N}}_{2}+2{h}^{+}+2^. {\text{O}}_{2}^{-}\to 2{\text{NO}}_{2}$$

12 $$4{\text{NO}}_{2}+{\text{O}}_{2}+2{\text{H}}_{2}\text{O}\to 4{\text{HNO}}_{3}$$

MO represents the metal oxide semiconductor photocatalytic catalyst. Under sunlight, electrons can be excited from the valence band (VB) of the catalyst to its conduction band (CB) and then migrate to the active site. In this way, the photoexcited electrons can reduce O_2_ to form ·O_2_^–^. The formed ·O_2_^–^ can combine with adsorbed N_2_ molecules to form NO_2_, and finally, NO_2_, O_2_, and water react to form HNO_3_.

An alternative mechanistic pathway is delineated as follows [72]:

13 $${\text{N}}_{2}+2{\text{H}}_{2}\text{O}+4{h}^{+}\to 2\text{NO}+4{\text{H}}^{+}$$

14 $$4\text{NO}+{3\text{O}}_{2}+2{\text{H}}_{2}\text{O}\to 4{\text{HNO}}_{3}$$

Within the confines of a defect-laden semiconductor photocatalytic catalyst, N_2_ engages in a dual modality of adsorption, encompassing both physical and chemical interactions. At sites characterized by an abundance of electrons—namely, defect sites—the N≡N triple bond is subjected to an activation process, culminating in bond cleavage and the subsequent generation of an adsorbed metastable intermediate. These intermediates, upon further activation, are converted into dinitrogen dioxide (N_2_O_2_), which then proceeds to react with molecular O_2_ and H_2_O, culminating in the formation of HNO_3_. Simultaneously, the vacancies in oxygen at the adsorption sites are ameliorated by the introduction of extrinsic oxygen atoms from the adsorbed water molecules, thus maintaining the integrity of the catalytic process.

Considering the initial advantages demonstrated by electrocatalytic nitrate reduction reaction to ammonia (ENRR) [73–75], photocatalytic NOR technology is progressively revealing its benefits within the emerging low-carbon and even negative-carbon nitrogen cycle. Compared to the Haber–Bosch process, the photocatalytic generation of nitrate requires only solar energy as the driving force. Under mild conditions of ambient temperature and pressure, photocatalytic NOR technology can precisely and efficiently convert nitrogen gas from the air into nitrate, involving only nitrogen, oxygen, and water, and can subsequently be coupled with nitrate electroreduction technology to further transform into ammonia.

This serial zero-carbon technology, on the one hand, significantly reduces the dependence of traditional ammonia production on fossil fuels, markedly decreasing greenhouse gas emissions and meeting the demands of sustainable development. On the other hand, the new technology can reverse the nitrogen cycle, enabling a distributed, low-energy catalytic conversion model that can meet regional economic needs for nitric acid or ammonia resources at any time.

## Element Strategies for Nitrogen Fixation Photocatalysts

**Fig. 5:**
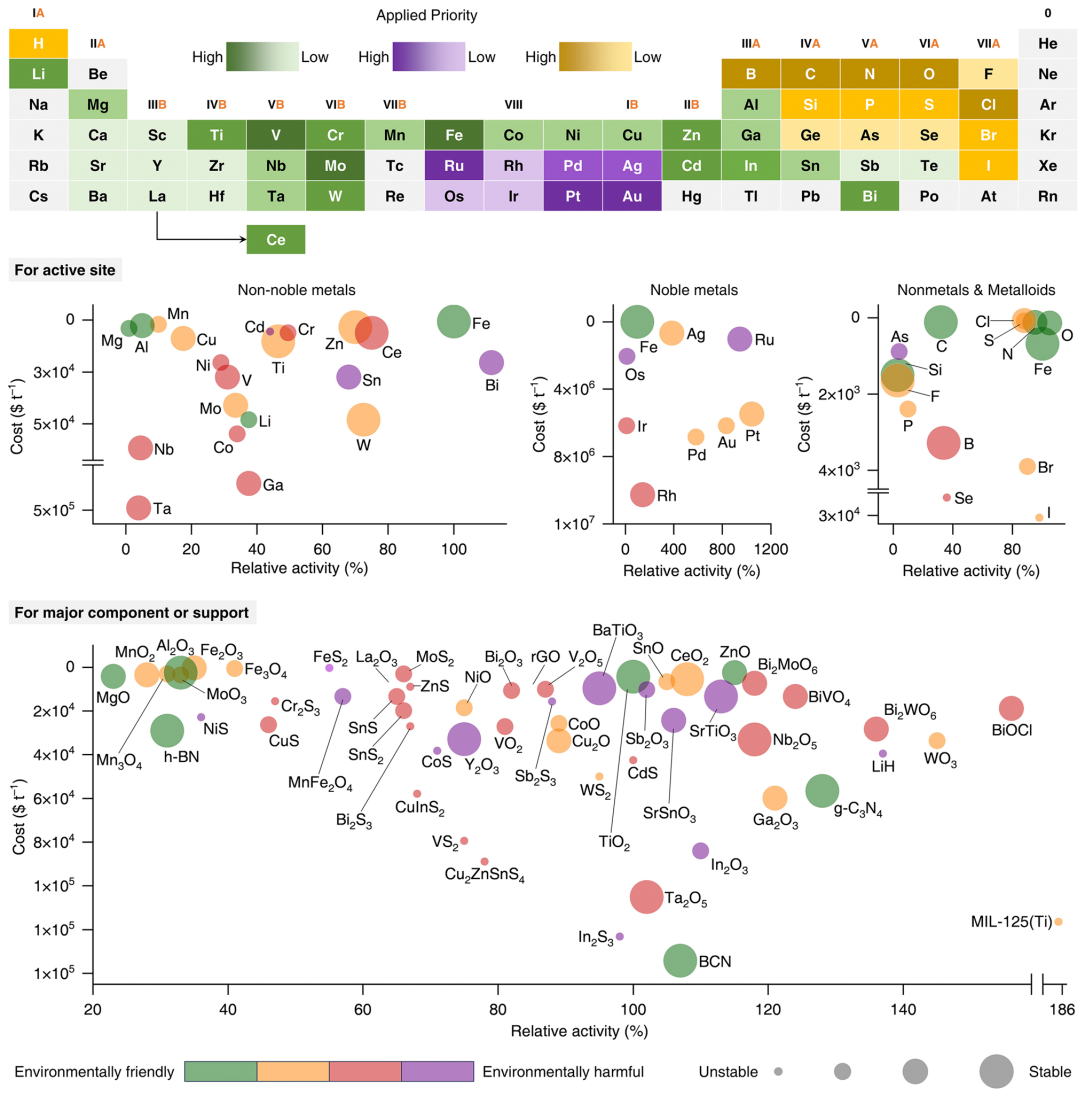
Classification and application strategies of elements suitable for constructing scale-up nitrogen fixation photocatalysts. Catalysts are typically composed of active sites and support materials. Each individual element or support material is evaluated across four dimensions: cost, activity, stability, and environmental friendliness. For non-noble metals, noble metals, non-metals, and semi-metals, the benchmark for catalytic activity is set with reference to iron. For a variety of catalyst bodies or support materials, the benchmark is set with reference to TiO_2_

To industrialize photocatalytic nitrogen fixation, the development and optimization of catalysts are crucial. By dissecting catalysts into active sites and supports for in-depth consideration, it becomes evident that balancing cost, performance, stability, and environmental friendliness from an elemental perspective is no easy feat. It requires comprehensive and meticulous planning, with a multi-pronged approach to achieve the desired outcome (Fig. 5).

Focusing first on the critical component of active sites, cost should be prioritized during scaled-up production. Therefore, elements such as gold (Au) and ruthenium (Ru), which have high market prices, must be approached with caution and should be avoided in large quantities whenever possible. Instead, the primary focus should shift to elements like iron (Fe) and bismuth (Bi). Not only are these elements relatively inexpensive, thereby significantly reducing raw material procurement costs and alleviating economic burdens on enterprises, but they also possess a certain level of inherent activity. This provides ample room for further optimization of catalyst performance through various technical means. For non-metal elements, the pressure from a cost perspective is not significant, and the rationale for selection mainly lies in their compatibility with other components of the catalyst.

Due to the competition from water oxidation–reduction, nitrogen fixation demands high reaction selectivity. When it comes to performance, choosing elements with strong adsorption and activation capabilities for nitrogen molecules is particularly urgent. Some metal elements, such as Ru and Fe, can interact with nitrogen molecules in a strong and efficient manner, thanks to their unique electronic structures, thus meeting the industrial requirements for catalytic efficiency. For precious metal elements, efforts should be concentrated on enhancing their dispersion and utilization rates. For non-precious metal elements, the focus should be on ingenious structural construction and reasonable element combinations. Non-metal or semi-metal elements typically do not directly provide active sites, but their combination with metal elements requires careful consideration. A well-designed metal–non-metal combination can significantly improve catalyst performance.

Moving on to stability, selecting elements with relatively stable chemical properties is key. These elements are less prone to aggregation, effectively preventing the decrease in catalytic activity caused by the clustering of active sites. Additionally, they are less likely to deactivate due to oxidation–reduction reactions, allowing them to maintain good catalytic performance over long periods and preventing the failure of active sites during prolonged, high-intensity reaction processes. This ensures the smooth and continuous progression of the entire catalytic process.

Since catalyst deactivation often culminates in the loss of active elements, the choice of different elements also determines the environmental pressure during the catalyst's usage period. The industrialization of catalysts must incorporate environmental friendliness into consideration. Taking Fe and Bi as examples, if the performance of catalysts based on these two elements as active sites is comparable, Fe would be the preferred choice solely from an environmental friendliness standpoint.

Shifting focus to the carrier aspect, this is equally indispensable. From a cost perspective, materials such as titanium dioxide (TiO_2_) and zinc oxide (ZnO), which are abundant in nature, easily obtained, and affordable, are favored as carriers. The widespread availability of these materials results in relatively low procurement costs, effectively reducing the overall cost of the catalyst system and enhancing the product's competitiveness in the market. It is worth noting that non-metal carriers, such as graphitic carbon nitride (g-C_3_N_4_), are promising, and with advancements in technology, they will become increasingly competitive once they gain a cost advantage.

In terms of performance, priority should be given to carriers that facilitate the uniform and efficient dispersion of active sites. This maximizes the actual utilization rate of active sites, allowing each one to fully exert its function and thereby enhancing catalytic effectiveness. Carriers with high porosity, such as alumina (Al_2_O_3_) and reduced graphene oxide (rGO), can greatly expand the contact area between reactants and active sites, akin to creating numerous "green channels" for reactants, aiding in the efficient progression of catalytic reactions.

Regarding stability, it is essential to select elements with robust structures that can withstand temperature and pressure shocks, as well as possess strong resistance to chemical corrosion. Only in this way can the carrier maintain its integrity in complex and harsh industrial environments, without being eroded or damaged by high temperatures, pressures, or various chemical substances, thus ensuring the overall stability of the catalyst. From this perspective, bismuth-based carriers (e.g., BiOCl) and titanium-based carriers (e.g., TiO_2_) are both excellent choices.

From an environmental friendliness standpoint, the characteristics of the carrier elements themselves, as well as their fabrication processes, should adhere to green environmental principles. When selecting carrier elements, it is crucial to ensure that they are non-toxic and harmless and that the fabrication processes are strictly controlled to avoid the introduction of toxic or harmful substances. The goal is to make the entire catalyst system fully compliant with environmental regulations and meet the public's expectations for environmental protection.

Overall, photocatalytic nitrogen fixation catalysts constructed around TiO_2_, BiOCl, ZnO, WO_3_, and g-C_3_N_4_ as support, and Fe, Bi, Sn, and Ru as active sites, show certain prospects for industrialization. However, further optimization remains a systematic project.

## Experimental Protocols for Photocatalytic Nitrogen Fixation

**Fig. 6:**
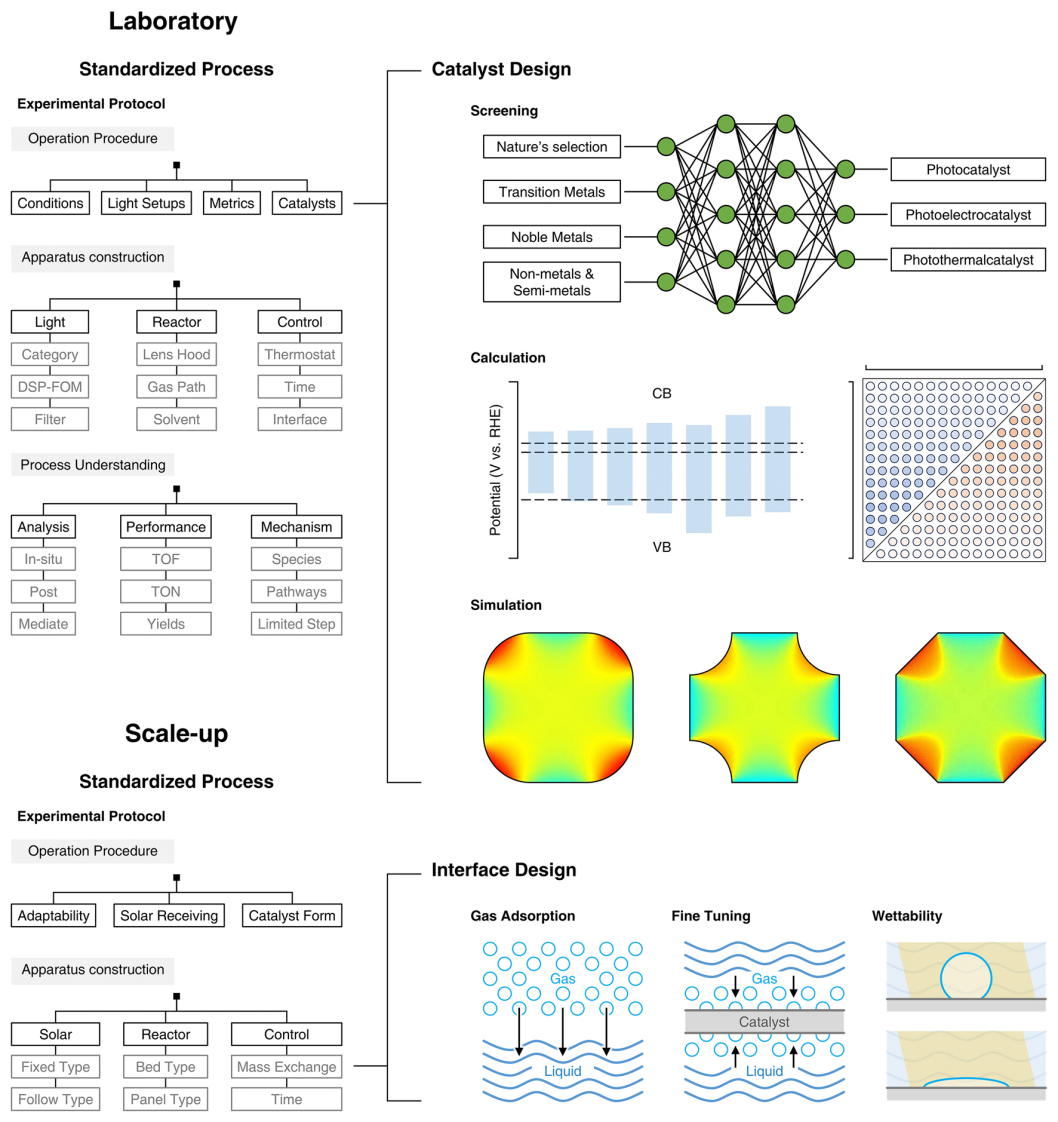
Scheme outlines a framework for transitioning photocatalytic nitrogen fixation research from the lab to the scaled-up production, guiding the industrialization of heterogeneous photocatalysis. In the laboratory section, standardized steps include experimental protocols (procedures, conditions like light settings, catalysts, and reactor setup, as well as process analysis such as yield and limiting steps). Catalyst design involves first-principles-based screening and optimization, covering various photocatalyst types, with simulations of band structures and performance relationships. In the scale-up section, steps include operational protocols and interface design, focusing on gas adsorption, structural tuning, and wettability adjustments

In the burgeoning domain of photocatalytic nitrogen fixation technology, the establishment of standardized protocols for experimental procedures, instrumental methodologies, and product analytics is of paramount importance from the inception of laboratory investigations to the realization of industrial applications. Such standardization not only augments the reproducibility and comparability of scientific endeavors but also fosters a profound comprehension of the photocatalytic efficiency and underlying mechanisms within the scholarly community. Furthermore, the adoption of standardized protocols is instrumental in surmounting technical impediments and facilitating the transition of bench-scale innovations to scalable engineering solutions. The implementation of these protocols has bolstered the competitive edge of photocatalytic catalytic technologies within the marketplace, propelling advancements within the industrial sector and providing a robust foundation of technical and regulatory guidance essential for the commercial viability of such technologies. The integration of advanced automation and artificial intelligence, exemplified by platforms such as RoboChem, has significantly amplified the efficacy of research endeavors [76]. Standardized operational protocols also ensure the safety and environmental sustainability of the processes involved, offering a critical technical framework for policy formulation and regulatory oversight, thereby enhancing the enforceability of pertinent legislative measures (Fig. 6).

### Catalyst Morphology and Apparatus

**Fig. 7:**
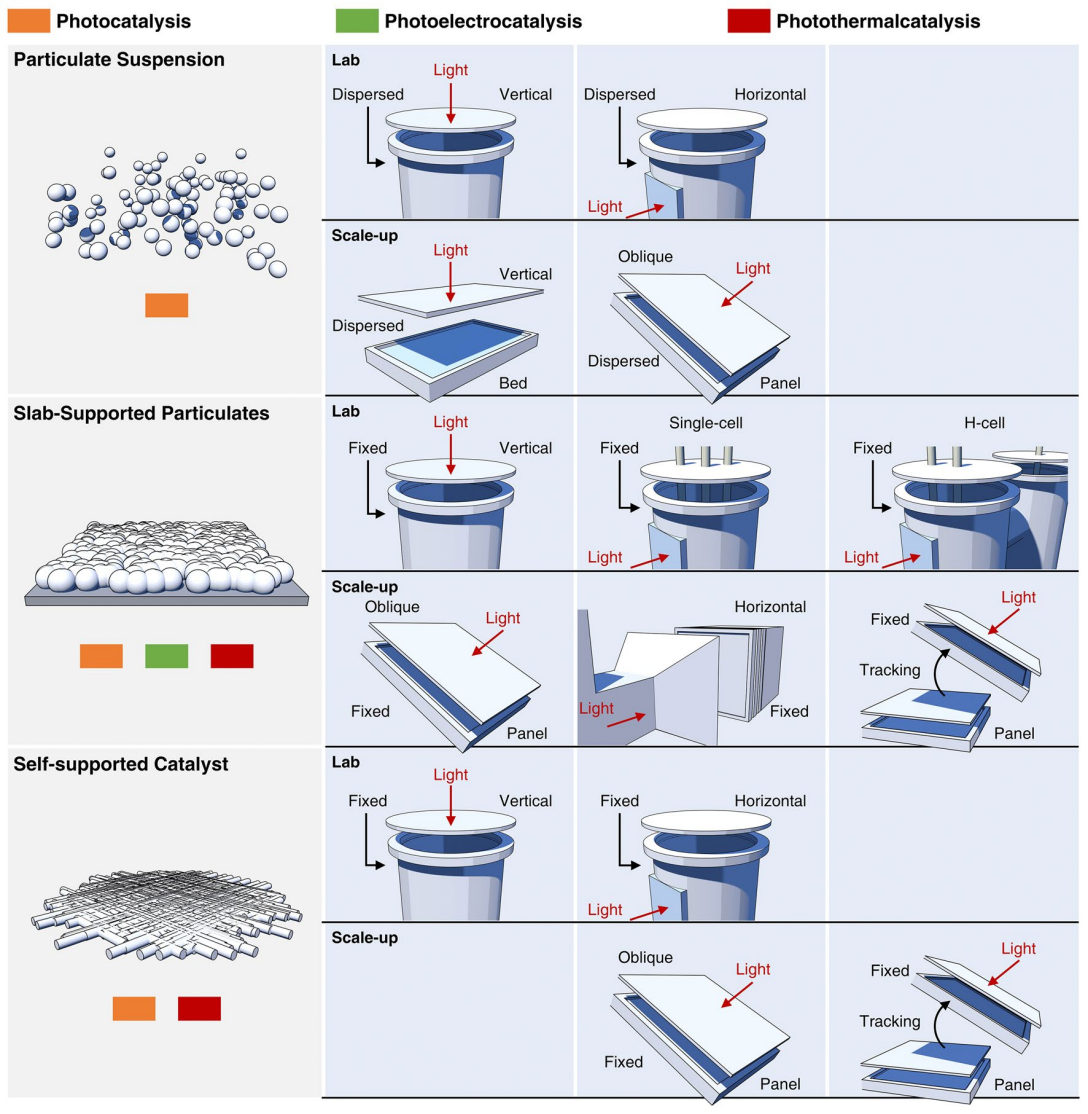
Scheme illustrates three catalyst morphologies and their applications in photocatalytic scenarios, covering both laboratory and scale-up applications. Particulate suspension catalysts enhance photon utilization efficiency in the lab by adapting to various light incidence angles. In scale-up applications, they utilize vertical or inclined lighting with sun-tracking mechanisms, and optimized stirring and reactor design to maintain catalyst dispersion. Slab-supported particulate photocatalysts improve light absorption and charge separation in the lab through strategic positioning of photoelectrodes or photothermal components. In scale-up, they employ fixed-angle lighting and reflective pathways to maximize light use, with optimized support structures to prevent particle movement. Self-supported catalysts accelerate electron transfer in the lab due to their uniform active site distribution. For scale-up, they focus on precise light source control and optimized three-phase interfaces to ensure efficient gas adsorption and catalyst stability

In the crafting of catalytic system for mild photocatalytic nitrogen fixation, the harmonious integration of catalyst morphology with apparatus design stands as a paramount endeavor, pivotal for the sustenance of stable, long-term operational excellence. For catalysts exhibiting various morphological characteristics, their applicability can be delineated into three principal categories that are optimally suited for different catalytic situations. These categories are specifically tailored to address the unique demands of photocatalytic processes, photoelectrochemical systems, and photothermal catalytic scenarios (Fig. 7).

Particulate suspension-based catalysts offer a range of benefits and challenges within practical applications. Their large surface area and superior dispersibility contribute to high light utilization rates, which is essential for optimal catalytic performance. The malleability of these materials, allowing for composite formation, modification, or alteration, further enhances their catalytic capabilities. However, their stability can be undermined by particle aggregation and sedimentation, impacting their reliability in certain applications. The implementation of these catalysts may also require additional separation steps post-reaction, potentially elevating operational complexity and costs. In catalysis, these particulate suspension-based catalysts are particularly adept at facilitating direct photocatalytic processes. In laboratory settings, the versatility of light sources necessitates the prioritization of apparatus design that accommodates both vertical and horizontal light incidences. When scaling to industrial applications, the natural light source characteristics often lead to the adoption of vertically fixed or angled lighting fixtures. To augment photocatalytic efficiency, the integration of sun-tracking mechanisms can be considered. Uniform dispersion of particulate catalysts is imperative to prevent aggregation in both laboratory and scaled-up processes. This can be achieved through the strategic optimization of stirring speeds, impeller configurations, and reactor internal structures, ensuring that the catalyst's active sites are optimally exposed to the reaction environment. The reactor's design should facilitate equitable light distribution across the catalyst surface, potentially necessitating a thoughtful arrangement of light sources and the use of reflective materials. These measures aim to maximize photo-efficiency and fully exploit the energy of solar photons.

Slab-supported particulate photocatalysts, characterized by their unique structural properties, demonstrate significant potential in the domain of photosynthesis. By strategically exposing specific crystal facets and constructing heterojunctions, these catalysts significantly improve light absorption and charge separation, thus enhancing their catalytic efficacy. Compared to conventional powder-suspended photocatalysts, slab-supported counterpart exhibits enhanced mechanical integrity and chemical robustness, which is conducive to their enduring performance in extended operational periods. Furthermore, the recovery of slab-supported particulate photocatalysts is more straightforward, simplifying their separation from the reaction milieu and reducing associated costs and environmental risks.

Despite these advantages, the application of slab-supported particulate photocatalysts faces certain challenges. Some materials are prone to photo-corrosion, which can induce structural instability and diminish performance under continuous illumination. The fabrication of these catalysts may also involve more complex methodologies and incur higher costs, which could impede their broader application. It is important to note that, despite their efficacy in charge separation, the recombination of photogenerated charge carriers remains a potential issue that can affect photocatalytic efficiency.

In photocatalytic processes, slab-supported particulate photocatalysts are applicable across various reaction types. In laboratory settings, the strategic placement of light sources and the positioning of photoelectrodes or photothermal components are pivotal for the design of experimental setups, which can be configured either in an integrated or separate manner. Scaling to industrial applications, device designs often incorporate fixed-angle or fixed-light-path lighting fixtures to accommodate the characteristics of slab structures. The incorporation of reflective pathways that track solar movement may also be considered to enhance light utilization. The design of devices for slab-supported particulate catalysts must focus on creating a support structure that ensures uniform dispersion of catalyst particles, maximizing light exposure and preventing particle movement or sedimentation. This involves selecting an appropriate pore size and distribution for adequate particle support. Additionally, optimizing the fluid dynamics within the device is crucial for maintaining efficient reactant–catalyst contact and avoiding uneven fluid distribution, thereby augmenting overall catalytic efficiency.

Self-supported catalysts have risen to prominence in catalysis, offering structural robustness and synthetic elegance. These catalysts feature a homogeneous distribution of active sites, which significantly boosts catalytic activity and expedites electron transfer, thus improving the overall catalytic efficiency. Under intense illumination, the superior durability of self-supported catalysts makes them prime contenders for sustained photochemical synthesis operations.

However, the synthesis of self-supported catalysts can be complex, involving advanced deposition techniques that may elevate process complexity and costs. Additionally, the accessibility of active sites may be limited by the catalyst's structural architecture, potentially affecting catalytic efficiency. Therefore, material selection requires careful consideration to ensure that the chosen materials provide the necessary catalytic activity while maintaining a self-supported structure.

Self-supported catalysts are particularly adept in both direct photocatalytic and photothermal catalytic processes. The apparatus design, whether at the laboratory or industrial scale, must consider the precise control of light sources, especially in photocatalytic processes. For photothermal catalysis, the apparatus should be designed to leverage the high photothermal conversion efficiency of the catalyst to achieve broad solar spectrum absorption. It is also essential to maintain a uniform temperature distribution within the reactor to prevent localized hot spots, which could compromise the catalyst's long-term stability and activity.

Furthermore, the apparatus design should optimize the gas–liquid-solid three-phase interface to enhance mass transfer efficiency, which is crucial for reactions involving gaseous reactants. The adjustment of surface properties, such as hydrophobic and hydrophilic characteristics, in conjunction with simulation techniques, ensures thorough wetting of the active sites and accelerates the adsorption of gaseous reactants, thereby enhancing catalytic potency.

Lastly, the choice of catalytic substrate is critical, as the interactions between the substrate and the catalyst can significantly influence the catalyst's stability and activity. The substrate's properties should be carefully selected to complement and enhance the performance of the self-supported catalyst.

### In Situ Detection

In the realm of photocatalytic nitrogen fixation, in situ real-time detection technologies have emerged as indispensable tools, facilitating the meticulous observation of the temporal dynamics of catalytic active sites throughout the reaction sequence. Such scrutiny affords researchers a profound comprehension of the underlying catalytic mechanisms, thereby enabling the elucidation of kinetic and thermodynamic nuances. The deployment of this technology is instrumental in the augmentation of catalyst selectivity and intrinsic activity. Through the meticulous fine-tuning of reaction parameters and the strategic engineering of catalyst architectures, there is a marked enhancement in the productive yield and the celerity of the target chemical transformations. Furthermore, in situ detection methodologies have shed light on the intricate phenomena of photogenerated charge carrier dynamics, including their separation, migration, and interfacial reaction kinetics. This insight provides a robust experimental foundation for the swift appraisal and sophisticated optimization of catalytic systems. The capability for real-time surveillance and modulation of reaction pathways, afforded by in situ techniques, is of paramount importance. It underpins the development of robust protocols for process control and paves the way for the translation of concern technologies from laboratory to scale-up.

#### Diffuse Reflectance Infrared Fourier Transform Spectroscopy

**Fig. 8:**
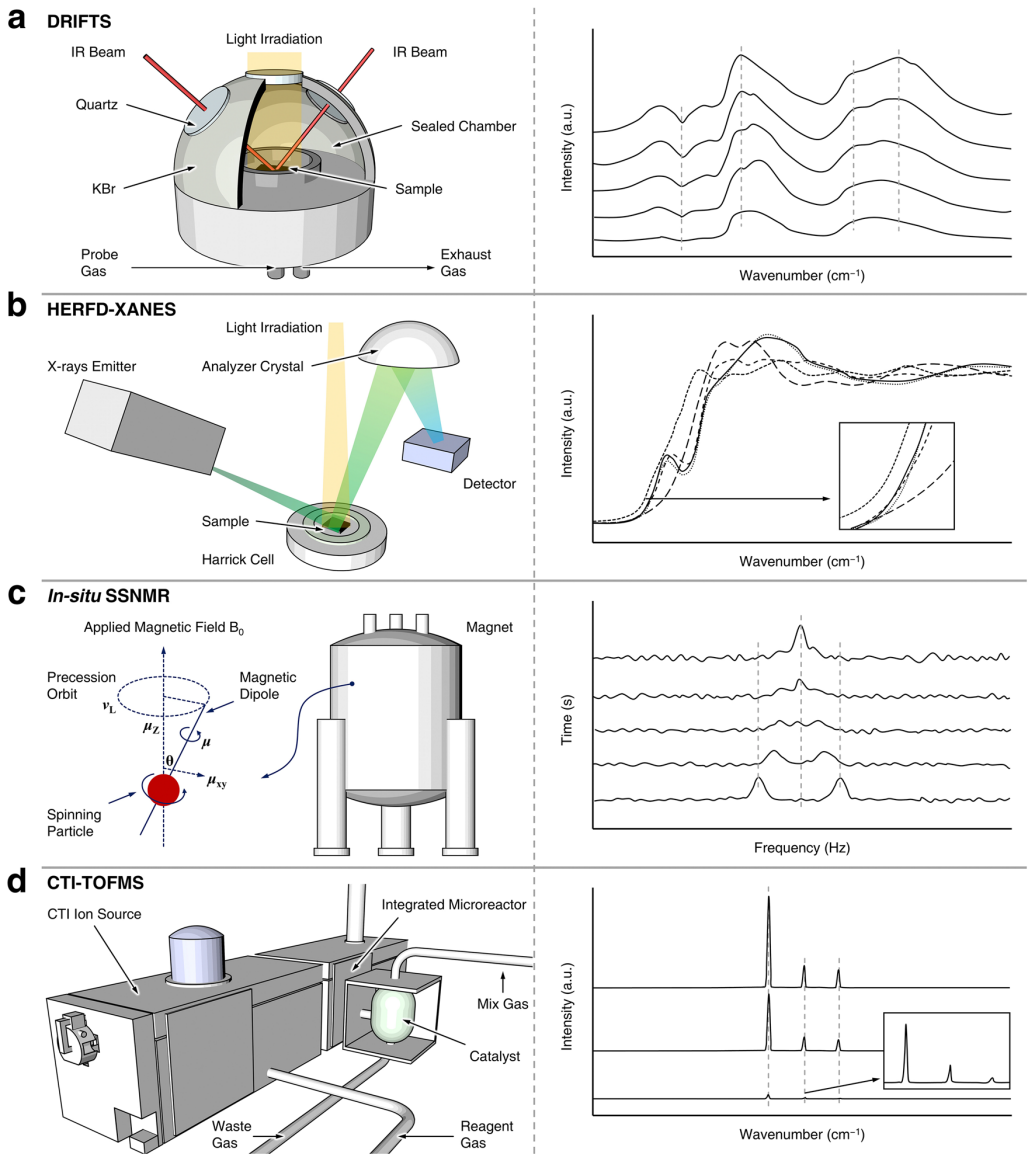
Schematic illustration of instrument construction and corresponding spectra for several advanced in situ detection methods, respectively. **a** DRIFTS, **b** HERFD-XANES, **c** In situ SSNMR, **d** CTI-TOFMS

Diffuse reflectance infrared Fourier transform spectroscopy (DRIFTS), as depicted in Fig. 8a, unequivocally elucidates the nitrogen binding process and the ensuing reduction under irradiation conditions. The paramount advantage of this analytical technique lies in its seamless amalgamation of the diffuse reflection methodology, infrared spectroscopy, and in situ infrared technology. This convergence allows for direct sample examination without the necessity for pressing or extensive processing, thereby preserving the intrinsic characteristics of the samples to the utmost extent. Consequently, in situ DRIFTS analysis across a spectrum of temperatures, pressures, and atmospheric conditions is more readily attainable compared to other infrared spectroscopic methodologies. The resultant spectral data visually corroborate the photocatalytic efficacy of the engineered materials, thereby providing a pivotal guide for subsequent experimental endeavors. As an authoritative modality for in situ characterization, it is imperative that all spectral acquisitions are conducted subsequent to a minimum of thirty minutes of argon (Ar) pre-treatment. This stringent protocol necessitates meticulous attention to several critical factors: (i) Illumination extinction and volatile contaminant removal: The light source must be extinguished to eliminate any potential volatile pollutants from the reaction chamber, utilizing an argon purge in a rigorous manner. (ii) Moisture absorption and trace hydrogen removal: Wet argon should be insufflated into the chamber to absorb trace quantities of, ensuring that water vapor is absorbed onto the sample surface under dark conditions. (iii) Superfluous moisture elimination: The chamber should be re-insufflated with argon to remove any excess H_2_O under dark conditions, ensuring a pristine sample environment. (iv) Nitrogen chamber saturation: The entire reaction chamber must be saturated with nitrogen in the absence of light to create an anaerobic environment conducive to the experimental conditions. (v) Continuous nitrogen introduction and photoactivation: N_2_ gas should be continuously introduced into the reaction chamber, followed by the activation of the light source to initiate the photochemical process.

#### High-Energy-Resolution Fluorescence-Detected X-Ray Absorption Near-Edge Structure and X-Ray Emission Spectroscopy

High-energy-resolution fluorescence-detected X-ray absorption near-edge structure (HERFD-XANES) and X-ray emission spectroscopy (XES) are two sophisticated analytical techniques that have garnered significant attention in the field of catalysis. HERFD-XANES, as illustrated in Fig. 8b, is capable of elucidating the alterations in the electronic structure of a catalyst and the intricate interactions between the catalyst and gas molecules during the course of a reaction. This capability provides a trove of direct experimental data, which is invaluable for deciphering the underlying catalytic mechanisms [77]. XES serves as a complementary technique to XANES, offering insights into the electronic density of the filled valence orbitals. Together, HERFD-XANES and XES have addressed a multitude of challenges associated with conventional X-ray analytical methods. These include the restricted irradiation area imposed by traditional X-ray window materials, the complexities inherent in the application of multi-component systems, and the limitations of XANES in discerning subtle spectral variations. The synergistic application of HERFD-XANES and XES is particularly well-suited for investigating solid–gas reaction systems that are pertinent to photocatalysis, thermos-catalysis, and photothermal synergistic catalysis. This encompasses a broad spectrum of applications, such as the degradation of gaseous pollutants and the harnessing of clean energy resources. The integration of these advanced spectroscopic techniques not only broadens the horizons of our understanding of catalytic processes but also paves the way for the development of more efficient and selective catalysts. The ability to scrutinize the electronic structure and orbital densities with such precision is a testament to the power of these methodologies in advancing the frontiers of catalysis research.

#### In Situ Solid-State NMR

In situ solid-state NMR (in situ SSNMR) spectroscopy represents an advanced analytical technique, pivotal for elucidating the molecular architectures of adsorbed reactants, transient intermediates, and resultant products on the surfaces of solid catalysts (Fig. 8c) [78, 79]. This methodology discerns catalytically active sites through the strategic introduction of adsorbates and reactants within the NMR rotor, facilitated by sophisticated resonance methodologies such as magic angle spinning (MAS) [80], cross polarization (CP) [81], decoupling techniques, and multiple quantum magic angle spinning (MQMAS) [82]. The synergistic application of in situ SSNMR with computational approaches, specifically density functional theory (DFT) calculations, enables the identification of reaction intermediates at the catalyst interface, thereby demystifying the underlying reaction mechanisms. Furthermore, the kinetic profiling of catalytic processes within confined nano-spaces can be accomplished through laser-enhanced SSNMR methodologies, employing an in situ flow configuration. The burgeoning application of in situ SSNMR within the domain of photocatalysis is noteworthy. This technology affords the opportunity to scrutinize the structural nuances of heteroatom-doped semiconductor catalysts, thereby unveiling the intricate correlations between photocatalytic efficacy and the nanostructural attributes of the photocatalyst.

#### Charge-Transfer Ionization Time-of-Flight Mass Spectrometry

To address the inherent limitations of conventional analytical techniques, such as diminished sensitivity, suboptimal temporal resolution, and the complexities associated with sample handling, the application of charge-transfer ionization time-of-flight mass spectrometry (CTI-TOFMS), integrated with a vacuum ultraviolet (VUV) light source, has been implemented for the real-time monitoring of NH_3_ synthesis, as depicted in Fig. 8d [83]. This state-of-the-art methodology is characterized by its exceptional resolving power, adept detection capabilities for multi-charged ions, expedited full-scan spectral acquisition, and stringent detection limits. During the catalytic process, the temporal dynamics of NH_3_ conversion rates in response to varying reaction temperatures are discernible with quantitative precision. Furthermore, the integration of intelligent self-regulating algorithms, coupled with the judicious presetting of reagent ion intensities, endows CTI-TOFMS with the potential to serve as a robust analytical instrument for the prolonged surveillance of photocatalytic reaction sequences. The acquisition of conversion rates for photochemically fixed NH_3_, alongside the elucidation of product distribution profiles at distinct stages of the reaction, facilitates a comprehensive assessment of the catalytic performance of the semiconductors under investigation. These data are instrumental in dissecting the catalytic mechanisms and delineating the reaction pathways.

### Post-Reaction Detection

Post-reaction analytical techniques are paramount for ensuring the veracity of scientific investigations, optimizing the design of catalysts, accelerating the technological translation to industrial applications, bolstering the replicability of research findings, underpinning sustainable energy initiatives, elucidating the underlying reaction mechanisms, and enhancing the precision of ammonia yield quantification. Precise detection methodologies circumvent spurious outcomes that may arise from residual contaminants, pH variations, or the presence of sacrificial reagents, thereby guaranteeing a dependable estimation of ammonia yields. Moreover, these techniques afford researchers the capacity to dissect the catalytic mechanisms operative in nitrogen fixation reactions with greater granularity, offering empirical support for the iterative refinement of catalysts. Standardized analytical protocols, in turn, augment the dissemination of scholarly knowledge and ensure the congruence of experimental outcomes across diverse research laboratories. As a conduit for the conversion of solar energy into a storable chemical form, photocatalytic nitrogen fixation is a burgeoning field that harbors significant potential for the development of renewable energy technologies. The deployment of rigorous and precise analytical methods for the detection of ammonia or nitric acid is imperative for the appraisal of the viability and efficacy of such technologies in real-world scenarios.

#### Unique NRR Detection Methods

NH_3_ quantification is an integral component of chemical analytical procedures, with the Nessler's reagent method and the indigo carmine assay being two prevalent techniques for its detection. The Nessler's reagent method is predicated on the formation of a yellow chromophore subsequent to the reaction between the reagent and ammonia, followed by the colorimetric quantification of ammonia concentration. However, this analytical approach is susceptible to interference from other constituents within the matrix, such as residual solvents and nitrogenous surfactants, which may confound the interpretation of results. Conversely, the indigo carmine assay, while also designed for the detection of ammonia, is subject to variability in its response due to the influence of metal ions at divergent pH levels, potentially skewing the accuracy of the assay outcomes. Consequently, when employing these methodologies, it is imperative to account for the potential confounding factors to ensure the veracity and reliability of the analytical data generated.

#### Unique NOR Detection Methods

UV spectrophotometry is a sophisticated analytical technique predicated on the pronounced absorption characteristics of the nitrate ion's –N = O functional group within the ultraviolet spectral region. The relationship between the absorbance of this moiety and the concentration of nitrate ions adheres to the Lambert–Beer law, thereby enabling the precise quantification of nitrate ions utilizing UV spectrophotometers. Furthermore, the potassium permanganate titration method exploits the redox reaction between nitrate ions and permanganate ions (MnO_4_^–^) under acidic conditions, where the permanganate is reduced to colorless manganese ions (Mn^2+^). This reaction serves as the basis for the quantitative determination of nitrate ions in a solution. The brown ring test, a classical laboratory procedure, involves the oxidation of ferrous ions to ferric ions by nitrate ions in a ferrous sulfate solution, with the concomitant reduction of nitrate ions to nitric oxide. The nitric oxide then reacts with ferrous sulfate to form a dark brown complex of ferrous nitrosyl sulfate, which is indicative of the presence of nitrate ions. Lastly, the UV light absorption method, utilized by certain online nitrate nitrogen analyzers, assesses the degree of absorption of UV light at specific wavelengths by the solution. This measurement is pivotal for the quantitative analysis of both nitrate and nitrite ions.

#### Common Detection Methods

Electrochemical methodologies, encompassing ion-selective electrode techniques, serve as pivotal analytical tools for quantifying specific ions within a solution. These methods leverage the selectivity of ammonium or nitrate ion-selective electrodes to ascertain the concentration of their respective ions. Ion chromatography further augments the analytical arsenal, employing sophisticated systems to effectuate the separation and detection of ammonium and nitrate ions, thereby facilitating precise quantification. Moreover, isotopic labeling experiments represent a sophisticated approach in the realm of chemical research. By incorporating nitrogen gas enriched with specific isotopes, these experiments enable the tracking of nitrogen's incorporation into ammonia or nitric acid within the reaction products. This capability is instrumental in verifying the occurrence of nitrogen fixation processes, thereby providing a robust mechanism for validating the underlying biochemical pathways. The integration of these analytical techniques not only enriches our understanding of chemical reactions but also enhances the precision and reliability of experimental outcomes.

## Parameters for Evaluating Photocatalytic Activity

Building upon the foundational work of our predecessors, we have formulated a comprehensive set of metrics designed to rigorously evaluate the efficacy of photocatalytic nitrogen fixation processes. It is imperative to meticulously examine the pivotal facets of energy and quantum conversion efficiency within these systems to ensure the robustness and reliability of our evaluative framework.

### Faraday Efficiency

In the context of nitrogen fixation, Faradaic efficiency (FE) denotes the efficacy with which electrical charges are conveyed to a NRR or NOR system. For all of the photocatalytic NRR and NOR processes, FE (%) is quantitatively delineated as the ratio of the actual yield of ammonia or nitrite to the theoretical yield predicated by the measured photocurrent. The concentration of NH_3_ (${c}_{{\text{NH}}_{3}}$) or NO_3_^–^ (${c}_{{\text{NO}}_{3}^{-}}$) (mol L^–1^) can be ascertained through the application of indophenol blue spectrophotometry. The total electrical charge (*Q*_total_) is derived from the photocurrent measurements. Given that the synthesis of one molecule of NH_3_ or NO_3_^–^ necessitates the transfer of three or five electrons, the FE can be empirically determined when the volume of the NH_3_ or NO_3_^–^ solution (${V}_{{\text{NH}}_{3}}\text{or}\,{V}_{{\text{NO}}_{3}^{-}}$) (L) is specified. Consequently, formulas for calculating *FE* of NRR and NOR, under these conditions, are articulated as follows:

15 $${\text{FE}}_{\text{NRR}}={c}_{{\text{NH}}_{3}}\times {V}_{{\text{NH}}_{3}}\times 3\text{F}/{(17Q}_{\text{photo}})$$

16 $${\text{FE}}_{\text{NOR}}={c}_{{\text{NO}}_{3}^{-}}\times {V}_{{\text{NO}}_{3}^{-}}\times 5\text{F}/(62{Q}_{\text{photo}})$$

where F represents the Faraday constant (96,485.3 C mol^–1^), the *Q*_photo_ (C) represents the theoretical consumption of electricity based on the photocurrent. Herein, the *Q*_photo_ can be calculated as follows:

17 $${Q}_{\text{photo}}={j}_{\text{photo}}\times A\times T$$

where *j*_photo_ represents the real-time photocurrent density (mA cm^–2^); A and T represent the irradiation area (cm^2^) and irradiation time (s), respectively.

### Product Yield Rate

The NH_3_ or NO_3_^–^ yield rate here indicates the molar number of NH_3_ or NO_3_^–^ produced per unit time and mass (mol s^–1^ g^–1^). It can be calculated as follows when the reaction time (t) is known:

18 $${\text{NH}}_{3}\text{ yield rate}={c}_{{\text{NH}}_{3}}\times {V}_{{\text{NH}}_{3}}/(17\times t\times {m}_{\text{cat}})$$

19 $${\text{NO}}_{3}^{-}\text{ yield rate}={c}_{{\text{NO}}_{3}^{-}}\times {V}_{{\text{NO}}_{3}^{-}}/(62\times t\times {m}_{\text{cat}})$$

Herein, the mass (*m*_cat_) (g) represents the active mass of photocatalysts and photothermal catalyst or the loadings on photoelectrodes.

### Standard Nitrogen Photo-Fixation Efficiency

The efficiency of nitrogen photo-fixation, including solar-to-ammonia and solar-to-nitrite, both of them can be denoted as *η*_STN_ (%), is a critical parameter that quantifies the capability of a catalytic system to harness solar energy and transform it into storable chemical energy. The *η*_STN_ is mathematically delineated by the ratio of the total chemical energy produced to the total incident solar irradiation energy, as articulated in Eqs. (20) and (21):

20 $${\eta }_{\text{STN}}=\frac{{\Delta }_{\text{f}}{G}_{\text{m}}^{\theta }\times {r}_{{\text{NH}}_{3}}}{{P}_{\text{irrad}}\times S}$$

21 $${\eta }_{\text{STN}}=({\Delta }_{\text{f}}{G}_{\text{m}}^{\theta }\times {r}_{{\text{NO}}_{3}^{-}})/({P}_{\text{irrad}}\times S)$$

where ${\Delta }_{\text{f}}{G}_{\text{m}}^{\theta }$ symbolizes the standard Gibbs free energy change (kJ mol^−1^) associated with the formation of ammonia or nitrite, which is empirically determined to be 16.45 kJ mol^−1^ (NH_3_) and 111.3 kJ mol^−1^ (NO_3_^–^). The term ${r}_{{\text{NH}}_{3}}$ or ${r}_{{\text{NO}}_{3}^{-}}$ represents the modified yield rate (mol s^−1^) of NH_3_ or NO_3_^–^, which accounts for the actual production without the normalization by mass, and is mathematically expressed by Eqs. (22) and (23):

22 $${r}_{{\text{NH}}_{3}}={c}_{{\text{NH}}_{3}}\times {V}_{{\text{NH}}_{3}}/(17\times t)$$

23 $${r}_{{\text{NO}}_{3}^{-}}={c}_{{\text{NO}}_{3}^{-}}\times {V}_{{\text{NO}}_{3}^{-}}/(62\times t)$$

Here, ${c}_{{\text{NH}}_{3}}$, ${V}_{{\text{NH}}_{3}}$ and ${c}_{{\text{NO}}_{3}^{-}}$, ${V}_{{\text{NO}}_{3}^{-}}$ denote the concentration (mol L^−1^) and volume (L) of ammonia or nitrite produced, respectively, and *t* represents the time (s) over which the reaction occurs. The variable *P*_irrad_ signifies the intensity of the incident photoirradiation (kW m^−2^), with the standard unit being 1 kW m^−2^, which is equivalent to 100 mW cm^−2^. Lastly, *S* refers to the illuminated surface area of the photoelectrode, measured in cm^2^, which is pivotal in determining the extent of solar energy absorption.

### Applied Bias Photon-to-Current Efficiency

The application of an external electrochemical workstation to provide a bias voltage has been demonstrated to significantly enhance the photoelectrochemical nitrogen fixation including NRR and NOR, thereby augmenting the yield of NH_3_ or NO_3_^–^. Consequently, the impact of the bias voltage (V) on the photo-fixation process of NH_3_ or NO_3_^–^ can be quantified through the metric termed as the applied bias photon-to-current efficiency (ABPE). This efficiency is calculated utilizing the following formula:

24 $$\text{ABPE}=\frac{{P}_{\text{out}}-{P}_{\text{in}}}{{P}_{\text{irrad}}}={j}_{\text{sph}}({V}_{\text{redox}}-{V}_{\text{bias}})/{P}_{\text{irrad}}$$

In this equation,* P*_out_ and *P*_in_ refer to the input and output photon flux (photons cm^−2^ s^−1^), the *j*_sph_ denotes the steady-state photocurrent density (mA cm^−2^), a critical parameter indicative of the system's ability to convert light energy into electrical current. The term *V*_redox_ (V) signifies the redox potential associated with the initial reduction of N_2_ to N_2_H or N_2_ to NO in an aqueous medium. The variable *V*_bias_ corresponds to the actual potential differential imposed across the working electrode (W.E.) and the counter electrode (C.E.) during the reaction. Lastly, *P*_irrad_ refers to the incident photon flux (photons cm^−2^ s^−1^), which is conventionally measured using standard protocols.

### Incident Photon-to-Current Efficiency

Materials need to absorb electromagnetic irradiation in a specific wavelength range to be stimulated to achieve nitrogen photo-fixation. Therefore, the determination of the incident photon-to-current efficiency (IPCE) (%) at fixed incident wavelength is an effective characteristic parameter to evaluate the photocatalytic performance. Herein, wavelength-based IPCE (*λ*) can be defined as the number of photoinduced electron carriers contributing to generated photocurrent by incident photons, which can be expressed as the ratio of total energy of converted electrons (*E*_TCE_) to total energy of the incident photons (*E*_TIP_):

25 $$\text{IPCE}\left(\lambda \right)=\frac{{E}_{\text{TCE}}}{{E}_{\text{TIP}}}=(({j}_{\text{photo}}(\lambda )/e)\times (hc/\lambda ))/{P}_{\text{irrad}}(\lambda )$$

herein, *λ* represents the specific wavelength of a incident light (nm); *j*_photo_ (*λ*) represents the photocurrent density recorded under the specific incident light irradiation (mA cm^–2^); *e* represents the elementary charge (1.602 × 10^–19^ C); *h* and *c* represent the Planck constant (6.626 × 10^–34^ J s) and the speed of light (3.0 × 10^17^ nm s^–1^), respectively; and *P*_irrad_ (*λ*) represents the PI intensity at the specific wavelength (mW cm^–2^).

### Absorbed Photon-to-Current Conversion Efficiency

The possible losses (reflection, refraction and transmission path) in irradiation in some cases will have a great impact on the IPCE values. To eliminate the interference of these losses on IPCE, the corrected value is called the absorbed photon-to-current conversion efficiency (APCE) (%), which represents the number of photoinduced electron carriers contributing to generated photocurrent by real absorbed incident photons with specific wavelength. Therefore, this equation for accurately calculating internal quantum efficiency is expressed as follows:

26 $$\text{APCE}\left(\lambda \right)=\frac{\text{IPCE}(\lambda )}{{R}_{\text{A}}(\lambda )}=\text{IPCE}(\lambda )/(1-{R}_{\text{flec}}-{R}_{\text{frac}}-{T}_{\text{path}})$$

where *R*_A_(*λ*) represents the real absorption at specific wavelength (nm), *R*_flec_ represents the reflection part, *R*_frac_ represents the refraction part, and *T*_path_ represents the part of transmission path. This parameter is more representative of the realistic photocatalytic process.

## Design Refinement of Catalysts

In the contemporary scientific landscape, a plethora of innovative design paradigms have been postulated to augment the nitrogen fixation efficacy of photocatalytic systems. These paradigms encompass a spectrum of strategies, including but not limited to: (1) nano-structuring to modulate the morphology at the nanoscale, (2) facet engineering to optimize the exposed crystal facets, (3) support effects to leverage the synergies with substrates, (4) the creation of single-atom catalysts to maximize atom utilization efficiency, (5) defect engineering to introduce controlled defects for enhanced catalytic activity, (6) heteroatom doping to alter the electronic structure and reactivity, (7) alloying to combine multiple elements for improved performance, and (8) the construction of heterojunctions to facilitate charge transfer and separation (Fig. 1). A comprehensive elucidation of the distinctive attributes of these octet design principles is delineated in Table 1. These methodologies are predicated on the axiomatic principles of augmenting electrocatalytic potency by amplifying the count of catalytic sites or by bolstering the intrinsic reactivity of individual sites. Concurrently, they aim to refine selectivity and optimize multiple reaction parameters through the fine-tuning of binding energies and the adsorption geometries of NRR and NOR intermediates. Furthermore, these strategies are often amalgamated to elicit a synergistic enhancement in the overall catalytic performance (see Table 1 for detail catalytic performances).

**Table 1:** Nitrogen photo-fixation catalysts summarized according to design refinement

Catalyst	*T* (°C)	*λ* (nm)	Feedstock	Scavenger	Scenarios	Products	Yields	References
OVs-rich TiO_2_	227	UV	N_2_	None	PTC^d^	NH_3_	0.83 μM g^−1^ h^−1^	[84]
Surface-OVs TiO_2_	40	280–420	N_2_	None	PC^b^	NH_3_	6.7 μM g^−1^ h^−1^	[85]
OVs-rich BiOBr NSs	25	Vis	N_2_	None	PC^b^	NH_3_	2084 μM g_cat_^−1^ L^−1^ h^−1^	[86]
NVs-induced g-C_3_N_4_	25	Vis	N_2_	None	PC^b^	NH_3_	1.24 mM g^−1^ h^−1^	[87]
Zn_0.1_Sn_0.1_Cd_0.8_S	30	400–800	N_2_	C_2_H_5_OH	PC^b^	NH_3_	0.40 g_cat_ L^−1^; 105 μM h^−1^	[88]
Mo_0.1_Ni_0.1_Cd_0.8_S	30	400–800	N_2_	C_2_H_5_OH	PC^b^	NH_3_	0.40 g_cat_ L^−1^; 71 μM h^−1^	[89]
CVs-rich S-doped g-C_3_N_4_	RT^a^	Vis	N_2_	CH_3_OH	PC^b^	NH_3_	5.99 mM g^−1^ h^−1^	[90]
Bi_5_O_7_Br NTs	RT^a^	> 400	N_2_	None	PC^b^	NH_3_	1380 μM g^−1^ h^−1^	[91]
(001) facet-BiOCl	25	UV–Vis	N_2_	CH_3_OH	PC^b^	NH_3_	0.67 g_cat_ L^−1^; 46.2 μM h^−1^	[92]
Bi-rich Bi_5_O_7_I	20	Uv–Vis	N_2_	CH_3_OH	PC^b^	NH_3_	0.5 g_cat_ L^−1^; 111.5 μM h^−1^	[93]
0.2 wt% Fe-doped TiO_2_	40	390–420	N_2_	None	PC^b^	NH_3_	10 μM g^−1^ h^−1^	[94]
0.4 wt% Co-doped TiO_2_	40	390–420	N_2_	None	PC^b^	NH_3_	6.3 μM g^−1^ h^−1^	[94]
0.4 wt% Mo-doped TiO_2_	40	390–420	N_2_	None	PC^b^	NH_3_	6.7 μM g^−1^ h^−1^	[94]
0.4 wt% Ni-doped TiO_2_	40	390–420	N_2_	None	PC^b^	NH_3_	2.9 μM g^−1^ h^−1^	[94]
2.3 wt% Fe-doped/TiO_2_	NA	NA	N_2_	None	PC^b^	NH_3_	1.0 g_cat_ L^−1^; 10 μM h^−1^	[95]
0.5 wt% Fe-doped/TiO_2_	80	UV	N_2_	None	PC^b^	NH_3_	6.0 μM g^−1^ h^−1^	[96]
0.01 wt% Fe^3+^-doped/TiO_2_	25	254	Air	C_2_H_5_OH	PC^b^	NH_3_	1.0 g_cat_ L^−1^; 400 μM h^−1^	[97]
2 wt% Mg-doped/TiO_2_	NA	UV	N_2_	None	PC^b^	NH_3_	0.67 g_cat_ L^−1^; 6.9 μM h^−1^	[98]
0.5 wt% Cr-doped/TiO_2_	80	UV	N_2_	None	PC^b^	NH_3_	2.6 μM g^−1^ h^−1^	[99]
10 wt% Ce-doped/TiO_2_	NA	UV	N_2_	None	PC^b^	NH_3_	0.80 g_cat_ L^−1^; 3.4 μM h^−1^	[100]
10 wt% V-doped/TiO_2_	NA	UV	N_2_	None	PC^b^	NH_3_	0.80 g_cat_ L^−1^; 4.9 μM h^−1^	[100]
0.24 wt% Ru-doped/TiO_2_	NA	UV–Vis	N_2_	C_2_H_5_OH	PC^b^	NH_3_	22.6 μM g^−1^ h^−1^	[101]
5 wt% RuCl_3_-modified TiO_2_	RT^a^^)^	UV–Vis	N_2_	Humic acid	PC^b^	NH_3_	10 μM g^−1^ h^−1^	[102]
Pt-doped/CdS	38	UV	N_2_	None	PC^b^	NH_3_	3.3 μM g^−1^ h^−1^	[103]
Pt-doped/GaP	38	UV	N_2_	None	PC^b^	NH_3_	5.0 μM g^−1^ h^−1^	[103]
Pt-doped/ZnO	RT^a^^)^	UV	N_2_	Na_2_SO_3_	PC^b^	NH_3_	860 μM g^−1^ h^−1^	[104]
Fe^3+^-doped/g-C_3_N_4_	25	400–800	N_2_	C_2_H_5_OH	PC^b^	NH_3_	5.4 mg L^−1^ h^−1^ g_cat_^−1^	[105]
Mo-doped W_18_O_49_ UTNW	RT^a^^)^	> 400	N_2_	Na_2_SO_3_	PC^b^	NH_3_	195.5 μM g^–1^ h^–1^	[106]
C-doped/HWO hybrids	RT^a^^)^	UV–Vis	N_2_	None	PC^b^	NH_3_	220 μM g^−1^ h^−1^	[107]
B-doped ECG diamond	RT^a^^)^	UV	N_2_	None	PC^b^	NH_3_	3.7 μg h^−1^	[108]
Ru_1_/d-UiO-66	RT^a^^)^	UV	N_2_	None	PC^b^	NH_3_	53.28 μmol g^–1^ h^–1^	[109]
TiO_x_/P3MeT	20	Vis	N_2_	None	PC^b^	NH_3_	19 μM m^−2^ h^−1^	[110]
TiO_x_/ClO_4_^−^-doped P3MeT	24	Vis	N_2_	None	PC^b^	NH_3_	3.2 mM m^−2^	[111]
Fe_2_O_3_/TiO_2_-supported γ-Al_2_O_3_	120	> 400	N_2_	None	PC^b^	NH_3_	NA	[112, 113]
Fe_3_O_4_/α-Fe_2_O_3_	30	UV–Vis	N_2_	None	PTC^b^	NH_3_	10 μM g^−1^ h^−1^	[114]
CdS/Pt/RuO_2_	30	Vis	N_2_	None	PC^b^	NH_3_	4.0 g_cat_ L^−1^; 620 μM h^−1^	[115]
Pt/CdS·Ag_2_S/RuO_2_	30	UV–Vis	N_2_	None	PC^b^	NH_3_	2.0 g_cat_ L^−1^; 1260 μM h^−1^	[116]
CdS/MoFe protein	NA	405	NA	None	PC^b^	NH_3_	315 μM g_protein_^−1^ min^−1^	[117]
[Mo_2_Fe_6_S_8_(SPh)_3_]^3+^-[Sn_2_S_6_]^4–^	RT^a^	UV–Vis	N_2_	NA	PC^b^	NH_3_	10.1 μM h^−1^	[118]
Mo_2_Fe_6_S_8_(SPh)_3_-Fe_4_S_4_-Sn_2_S_6_]^4–^	RT^a^	UV–Vis	N_2_	NA	PC^b^	NH_3_	18.8 μM h^−1^	[119]
ZnSnCdS/g-C_3_N_4_	30	400–800	N_2_	C_2_H_5_OH	PC^b^	NH_3_	0.4 g_cat_ L^−1^; 168 μM h^−1^	[120]
ZnMoCdS/g-C_3_N_4_	25	400–800	Air	C_2_H_5_OH	PC^b^	NH_3_	0.4 g_cat_ L^−1^; 78 μM h^−1^	[121]
MgAlFeO/g-C_3_N_4_	30	400–800	N_2_	C_2_H_5_OH	PC^b^	NH_3_	0.4 g_cat_ L^−1^; 167 μM h^−1^	[122]
W_18_O_49_/g-C_3_N_4_	NA	UV–Vis-NIR	N_2_	C_2_H_5_OH	PC^b^	NH_3_	0.4 g_cat_ L^−1^; 58 μM h^−1^	[123]
2.4 wt% Ga_2_O_3_-DBD/g-C_3_N_4_	NA	UV–Vis	N_2_	C_2_H_5_OH	PC^b^	NH_3_	0.4 g_cat_ L^−1^; 113 μM h^−1^	[124]
(-C≡N)/K^+^-modified g-C_3_N_4_	NA	Vis	N_2_	None	PC^b^	NH_3_	3.42 mmol g^−1^ h^−1^	[125]
2D/2D rGO/g-C_3_N_4_	30	400–800	N_2_	EDTA-Na_2_	PC^b^	NH_3_	9.276 mg L^−1^ h^−1^ g_cat_^−1^	[126]
Fe@3D graphene	200	UV	N_2_/H_2_	None	PC^b^	NH_3_	24 μM g^−1^ h^−1^	[127]
Fe-Al@3D graphene	200	UV	N_2_/H_2_	None	PTC^d^	NH_3_	25.3 μM g^−1^ h^−1^	[128]
Au/TiO_2_-OV	RT^a^	420–700	N_2_	CH_3_OH	PTC^d^	NH_3_	78.6 μM g^–1^ h^–1^	[129]
5 wt% Ru@n-InGaN	10	290–380	N_2_	None	PC^b^	NH_3_	120 μM g^–1^ h^–1^	[130]
p-GaP	NA	Vis	N_2_	AlCl_3_	PC^b^	NH_3_	NA	[131]
BaTiO_3_	40	UV	N_2_	None	PEC^c^	NH_3_	0.87 μM g^–1^ h^–1^	[132]
BaTiO_3_-OV	RT^a^	Piezo + Vis	N_2_	None	PEC^c^	NH_3_	106.7 μM g^–1^ h^–1^	[133]
RuO_2_-NiO-BaTiO_3_	40	UV	N_2_	None	PEC^c^	NH_3_	2.6 μM g^–1^ h^–1^	[134]
FeTiO_3_	NA	*λ* ≥ 400 nm	N_2_	C_2_H_5_OH	PEC^c^	NH_3_	0.57 μM cm^–2^ h^–1^	[135]
Au-NPs/Nb-SrTiO_3_/Ru	RT^a^	550–800	N_2_	C_2_H_5_OH	PEC^c^	NH_3_	1.1 nM cm^–2^ h^–1^	[136]
Au-NPs/Nb-SrTiO_3_/Zr/ZrO_x_	RT^a^	550–800	N_2_	C_2_H_5_OH	PEC^c^	NH_3_	6.5 nM cm^–2^ h^–1^	[137, 138]
Bi_2_MoO_6_	RT^a^	*λ* ≥ 420 nm	N_2_	None	PEC^c^	NH_3_	1.3 mM g^–1^ h^–1^	[139]
TiO_2_/Au/a-TiO_2_	RT^a^	Vis	N_2_	None	PEC^c^	NH_3_	13.4 nM cm^−2^ h^−1^	[140]
Cs_2_O-promoted Os-Au hybrid	60	*λ* ≥ 450 nm	N_2_/H_2_	None	PEC^c^	NH_3_	2685 μM g^–1^ h^–1^	[141]
GNP/bSi/Cr	NA	UV–Vis	N_2_	None	PEC^c^	NH_3_	0.78 μM cm^−2^ h^−1^	[142]
p-Si/Ti/PTFE/Au	RT^a^	Vis	N_2_	None	PEC^c^	NH_3_	18.9 μg cm^−2^ h^−1^	[143]

^a)^RT is denoted as room temperature

^b)^PC is denoted as photo-catalysis

^c)^PEC is denoted as photoelectro-catalysis

^d)^PTC is denoted as photothermal catalysis

### Construction of Defects

Defect construction plays a pivotal role in augmenting the efficacy of photocatalysts through multiple mechanisms: it broadens the spectral absorption range into the visible and near-infrared spectrum, thus enhancing solar energy harnessing; functions as a charge-separation medium to mitigate electron–hole recombination and extend the lifetime of photogenerated carriers; modulates the electronic structure to refine catalytic properties; fortifies the structural integrity for enduring performance; adjusts the bandgap to optimize redox potential; and tailors surface characteristics to augment reactant adsorption. The judicious manipulation of defects facilitates the rational design of photocatalysts with heightened efficiency for applications in energy conversion and environmental amelioration.

#### Oxygen Vacancies

Vacancies as a type of defects are often used to improve the performance of photocatalytic materials. The elements that can form vacancies usually come from groups IIIA to VIIA. The defects-rich semiconductor catalysts are classified according to their vacancy elements and defect forms.

**Fig. 9:**
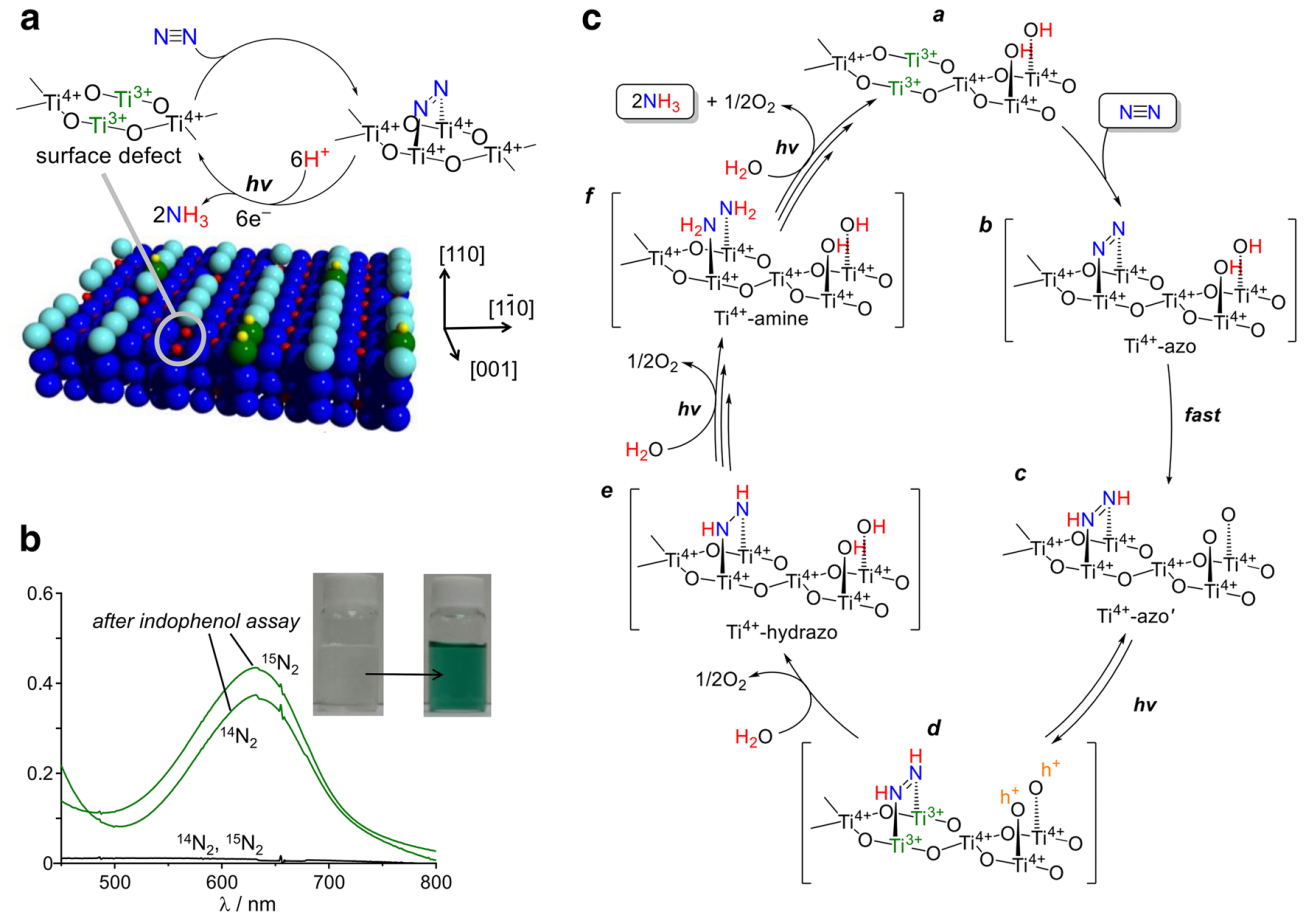
**a** Proposed photocatalytic cycle for N_2_ fixation on the rutile TiO_2_ (110) surface. **b** Photographs show the color change of the ^15^N_2_ solutions by the indophenol assay. **c** Proposed mechanism for photocatalytic fixation of N_2_ around the surface oxygen vacancies of TiO_2_. Reproduced with permission [85]. Copyright 2017 American Chemical Society

For metal-oxide semiconductor materials, the concept of defects and vacancies is similar, which could be denoted as OVs. If O exists in other types of materials, such as CdS, then O may enter the lattice gap and become interstitial O defect, or O may replace S and become impurity O defect. This situation is no longer called OVs, but only O defects. OVs are formed spontaneously in the synthesis procedure, and most of them are constructed intentionally. Although, some early researches ignored the effects of O defects on NRR performance, its effects were soon discovered [84, 94]. An important study elaborated a detailed catalytic mechanism of OVs-rich TiO_2_ under UV illuminations by Hirai and co-workers [85]. Surface-loaded OVs make the easy transformation of Ti^4+^ to Ti^3+^ in the original TiO_2_ (Fig. 9a). This simple and stable defective material has an obvious catalytic effect for nitrogen photo-fixation (Fig. 9b). Each Ti^3+^ exists between bridged OVs, when one molecule of N_2_ is adsorbed, Ti^3+^ becomes Ti^4+^ and N≡N bond is cleaved with the electron transfer. Accompanied by the production of Ti^4+^-azo’ species, H provided by Ti–OH pair rapidly interacted with the adsorbed N_2_. The photoexcitation of TiO_2_ substrate with Ti^4+^-azo’ species produces e^−^ and h^+^ pair at CB and VB, respectively. VB h^+^ are located on the Ti–OH pair and CB e^−^ captured on surface defects revive the inlaid Ti^3+^. The dissociation of N=N is accompanied by the Ti^4+^-azo’ species production and h^+^-assisted water oxidation simultaneously. After several photocatalytic steps, NH_3_ is obtained eventually, and Ti^4+^ are reconverted to Ti^3+^ and undergo the next photocatalytic cycle (Fig. 9c). Ti^3+^ as active sites greatly promote the N_2_ adsorption and activation. The probing mechanism clarifies the positive effects of OVs on photocatalytic NRR and guides the proper use of defect-engineering strategy. Due to the wide existence of OVs in metal-oxide semiconductors, fine adjustment of internal OVs is especially useful for performance enhancement.

Bi (II) species (such as BiO quantum dots) could activate and hydrogenate N_2_ without using any sacrificial reagent. Moreover, the derivative bismuth oxyhalides (BiOX, X = Cl, Br, or I) prepared by BiO and hydrogen halides as 2D semiconductor are also attractive [144–148]. BiOX has a unique 2D-layered structure: the [Bi_2_O_2_]^2+^ layer is the main structure with halogen atom X (X = Cl, Br, or I) on both sides, and X–Bi–O–Bi–X as the basic unit is staggered stacking in (001) direction. Defects’ construction as primary means for photocatalytic modification was widely used. A typical work demonstrated that OVs embedded BiOBr nanosheets (NSs) could realize the efficient nitrogen photo-fixation [86]. DFT calculations show that the original N≡N bond is elongated after being adsorbed on the OVs. This illustrates the occurrence tendency of N≡N bond cleavage on OVs-rich BiOBr (001) facets is higher. The suitable band position of BiOBr can effectively inhibit the recombination of photoexcited electrons and holes without the assistance of scavenger and co-catalysts. Moreover, some researches have made unremitting efforts to maintain the stability of OVs in the catalytic procedure [91, 93, 149, 150].

#### Nitrogen Vacancies

**Fig. 10:**
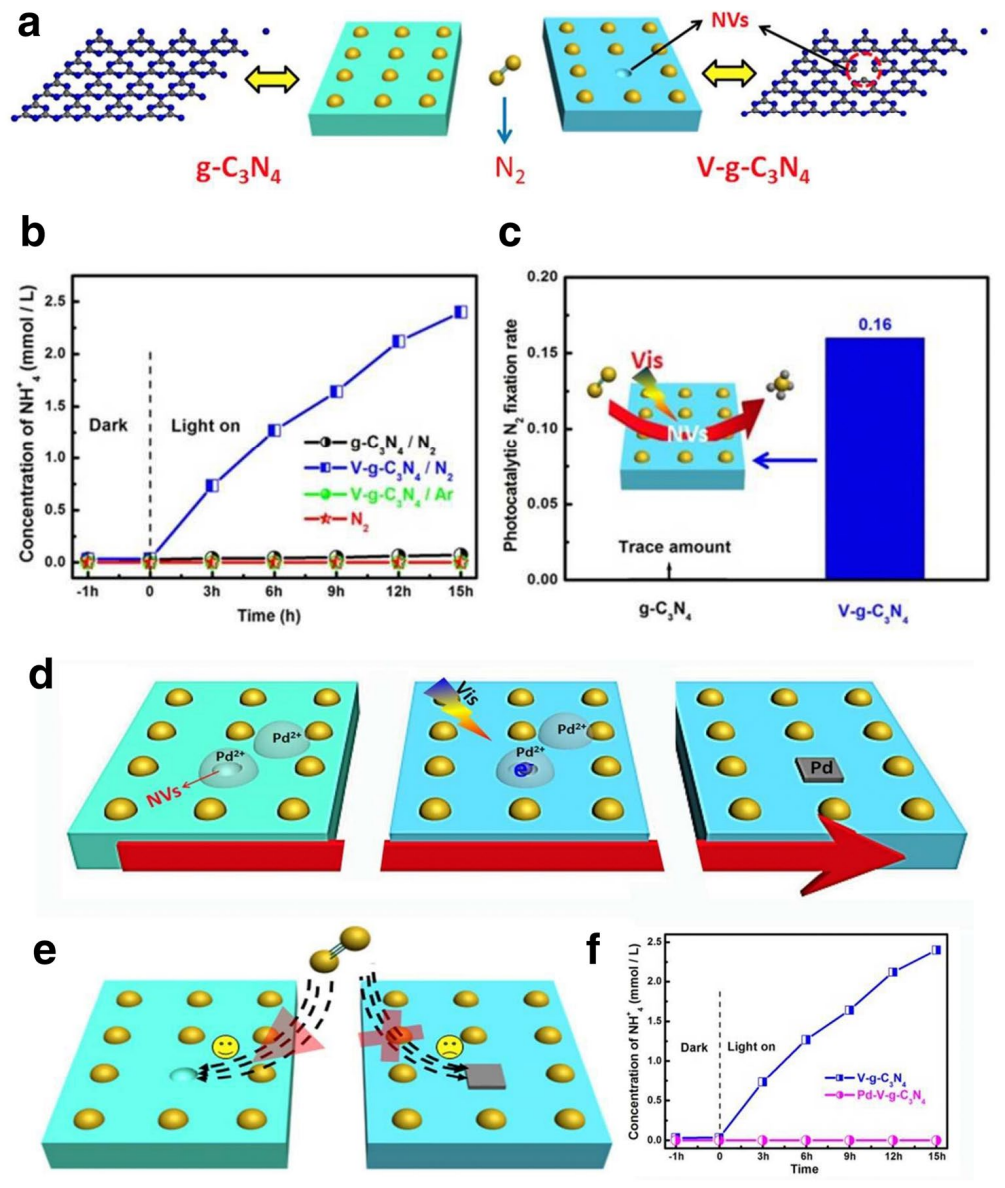
**a** Schematic of g-C_3_N_4_ and V-g-C_3_N_4_. **b** Concentration of generated NH_4_^+^ in different systems. **c** Photocatalytic N_2_ fixation rate of g-C_3_N_4_ and V-g-C_3_N_4_. **d** Pd particles can be selectively deposited onto NVs. **e** N_2_ will not be adsorbed on the Pd particle because of the considerable work-function. **f** Loading Pd onto V-g-C_3_N_4_ can completely suppress photocatalytic N_2_ fixation. Reproduced with permission [87]. Copyright 2015 Royal Society of Chemistry

The construction of nitrogen vacancies (NVs) is considered as another choice to enhance photocatalytic performance [151–153]. NVs possess multiple advantages for photocatalytic nitrogen fixation with following aspects: (i) The N defect matches both the shape and the size of the N atom. It means that materials with NVs may have similar abilities of molecular imprinted polymers to specifically recognize, selectively adsorb, and activate N_2_; (ii) As an indispensable ability of defects, it can effectively trap photoexcited electrons to inhibit the recombination of electrons and holes; (iii) NVs-construction is easy to operate, usually by heating or spontaneous formation under N_2_ atmosphere. A typical work was reported by Wang et al. [87]. The NVs of g-C_3_N_4_ (NV-g-C_3_N_4_) was obtained by calcination in N_2_ atmosphere (Fig. 10a). The highlight of this work is the source of NV-g-C_3_N_4_ NRR catalytic activity was described in detail. Firstly, the NV-g-C_3_N_4_ was tested and found to have a good NRR catalytic selectivity (Fig. 10b). Further characterizations were applied to confirm the role of NVs in catalysis. Secondly, the individual g-C_3_N_4_ was tested and found to have no catalytic activity (Fig. 10c). Thirdly, when the NVs are filled with Pd, the material no longer has photocatalytic activity again (Fig. 10d-f). It is speculated that the catalytic activity is derived from NVs. Moreover, DFT calculations provide the theoretical support for the reaction mechanism of NV-g-C_3_N_4_. The simulated adsorption procedure shows that when a N_2_ molecule is adsorbed on NVs, two σ bonds are formed between the N_2_ molecule and nearest two C atoms. Tight bonding and N≡N bond length increase theoretically confirmed the specific adsorption of N_2_ by NV-g-C_3_N_4_. In fact, g-C_3_N_4_ has photo-reactivity to other gases, but NV-g-C_3_N_4_ can only specifically catalyze N_2_, which further confirmed the selectivity of NVs-rich NV-g-C_3_N_4_. This strategy also applies to other systems [154, 155].

#### Sulfur Vacancies

Sulfur vacancies (SVs) usually exist in defective metal sulfides. The multi-component metal sulfides Zn_0.1_Sn_0.1_Cd_0.8_S [88] with abundant SVs realized the mild photocatalytic nitrogen fixation under visible light. The chemisorption and activation of N_2_ could successfully occur on superficial SVs. Electrons are soon captured by the SVs, preventing the combination of excitation electrons and holes. So, the NRR catalytic performance could be greatly improved. The introduced SVs have successfully elongated the N≡N bond length, and the number of SVs is linearly positively correlated with NH_3_ production. A similar application presents ternary metal sulfide (Mo_0.1_Ni_0.1_Cd_0.8_S [89]) as a semiconductor nitrogen photo-fixation catalyst.

#### Carbon Vacancies

There are few carbon-based semiconductor materials with photocatalytic response signals. The application of carbon vacancies (CVs) mainly appears in the modification of g-C_3_N_4_. For example, CV-rich g-C_3_N_4_ NSs with lattice-embedded S (SCNNSs) was synthesized by gas-phase polymerization [90]. As expected, the introduction of S atom in g-C_3_N_4_ lattice structure improves the N_2_ adsorption performance. After further construction of CVs, it was found that the N_2_ chemisorption performance was greatly enhanced. The occurrence of CVs redistributes the charge density of material. Electrons are transferred to N_2_ via CVs, and N_2_ is excited to form high-energy intermediates [90]. Theoretically, CVs have larger size than NVs, which can effectively adsorb N_2_ molecules and enhance the activation ability. The groups IIIA to VIIA also include many other elements. Semiconductor photocatalytic materials with such elements as vacancies to construct catalytic active centers are worthy of further discussion.

#### Other Types of Defects

The photocatalytic NOR was reported by Xie et al. [72]. The body defects were constructed in this research. The adsorption of N_2_ and activation of N≡N bonds were realized by using ultrathin pothole-WO_3_ NSs. Atomic size pores enhance the catalytic performance of the WO_3_ NSs so that realize another type of conversion. Through DFT calculation, reaction pathway based on photogenerated-hole oxidation mechanism can be expressed as follows:

27 $${\text{1st}}:{\text{ N}}_{{2}} + {\text{ 2H}}_{{2}} {\text{O }} + {\text{ 4h}}^{ + } \to {\text{ 2NO }} + {\text{ 4H}}^{ + }$$

28 $${\text{2nd}}:{\text{ 4NO }} + {\text{ 3O}}_{{2}} + {\text{ 2H}}_{{2}} {\text{O }} \to {\text{ 4HNO}}_{{3}}$$

29 $${\text{Total reaction}}:{\text{ 2N}}_{{2}} + {\text{ 3O}}_{{2}} + {\text{ 6H}}_{{2}} {\text{O }} + {\text{ 8h}}^{ + } \to {\text{ 4NO}}_{{3}}^{-} + {\text{ 12H}}^{ + }$$

The dangling bonds existing in the hole-rich structure realize the chemisorption and activation of N_2_. The activated N≡N bonds are converted to N–N bonds and finally to NO. The NO molecule spontaneously converts to nitrate with the participation of O_2_ and H_2_O. The participation of high momentum electrons in photocatalysis realized the acceleration of activation and cleavage procedure. Compared with direct nitrogen-to-ammonia (*η*_STN_) conversion, through different reaction mechanisms, nitrate as another important industrial raw material could also be prepared by mild nitrogen photo-fixation effectively. To sum up, these works not only clarify the importance of defects construction in photocatalytic NRR process, but also contribute to the advancements of selectivity and durability in the design of photocatalysts for long-term preparation procedure.

**Fig. 11:**
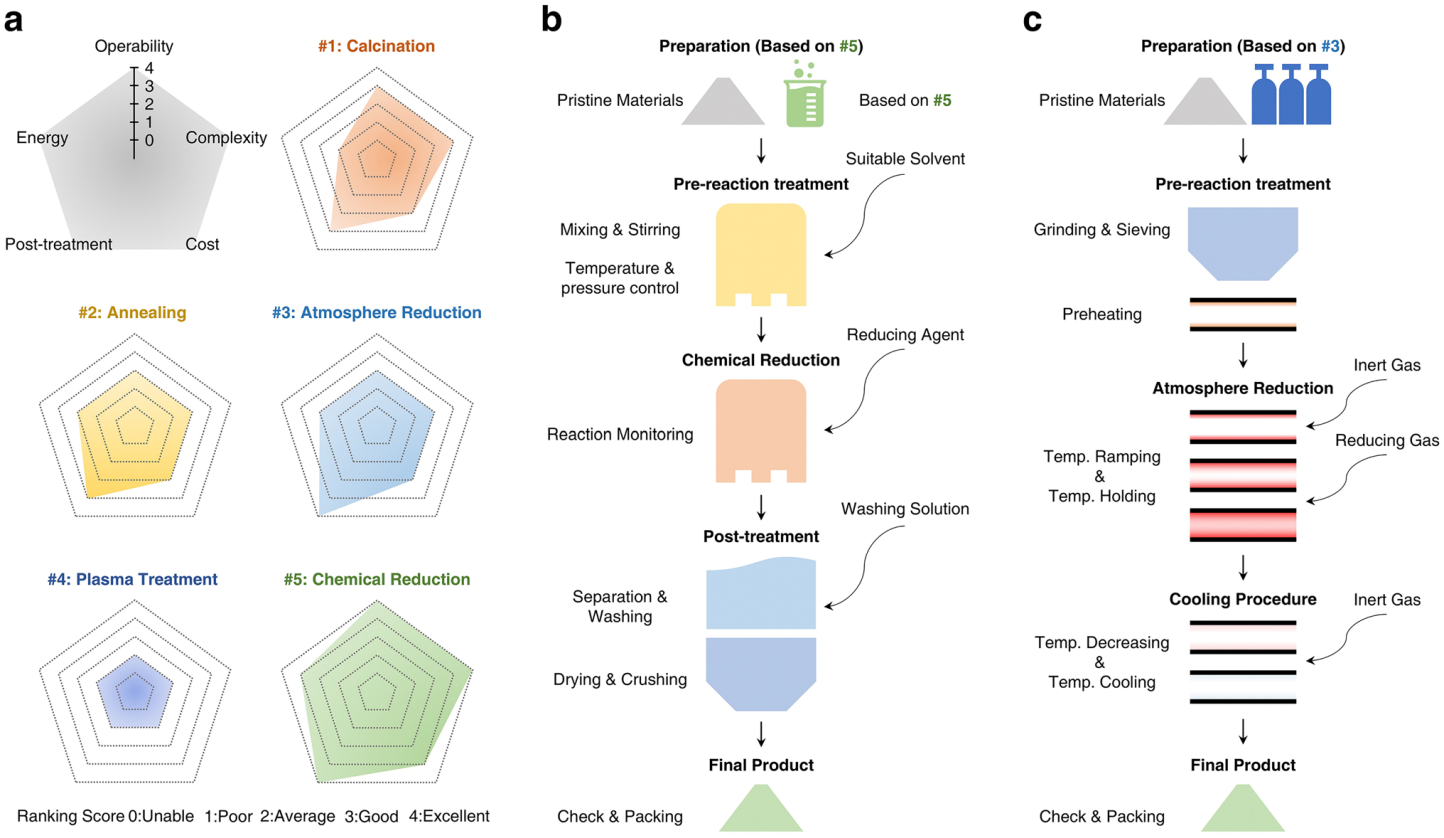
**a** Radar chart illustrates the evaluation details of different scaled-up approaches suitable for introducing defects across five important dimensions. The ranking scores for each dimension range from 0 to 4, corresponding to "unable", "poor", "average", "good", and "excellent" respectively. **b** Scheme of the general process flow for scale-up production using chemical reduction methods. **c** Scheme of the general process flow for scale-up production using atmosphere-assisted thermal reduction methods

Defect construction, as a simple and effective means to enhance catalytic performance, is a refinement method with great industrial prospects. From the perspective of scaled-up preparation, thermal treatment methods (including high-temperature calcination, rapid thermal annealing, and atmosphere-assisted thermal reduction), plasma treatment, and chemical reduction are the most promising approaches. We assess these potential scale-up approaches from the perspectives of operational feasibility, energy cost, system complexity, post-treatment requirements, and investment expenditure (Fig. 11a). Thermal treatment methods hold promise overall, yet they are characterized by high energy consumption and low efficiency. Firstly, they struggle to align with the trend toward carbon-free industries. Secondly, the reduction of total expenditure remains a significant challenge. Despite the high efficiency of plasma treatment, its performance in scaled-up production is suboptimal. Operationally, it necessitates specialized equipment, intricate parameter tuning, dependence on specific environmental conditions, and a high level of operator expertise. Furthermore, the system is characterized by high energy consumption, increased complexity, reduced operability, and significant expenditure. The post-processing is also problematic, necessitating meticulous cleaning and adjustment. Overall, the chemical reduction method stands out with its advantages. It is easy to operate, requires mild conditions, and uses simple equipment. After pre-mixing, the reaction can proceed by adjusting basic parameters (Fig. 11b). In most cases, the reaction can be carried out at room temperature, which means low energy consumption and low expenditure. The product can be separated and purified simply by filtration and washing, thereby ensuring constant quality. Due to the system's simplicity, both setup and maintenance are easy, which facilitates invest control and enhances competitiveness. As a supplement, for solvent-free scenarios and demands for high purity and precise control, atmosphere-assisted thermal reduction is a promising choice. This method is uniform, efficient, simple, and requires virtually no post-treatment (Fig. 11c). In terms of energy conservation, the process can be optimized by adjusting the thermal atmosphere temperature, and the residual heat from water vapor can be recovered and reused (taking hydrogen as an example). Moreover, with the development of green hydrogen technology, the cost of reducing agents has further decreased, which not only benefits cost reduction but also facilitates resource recycling.

### Nanostructuring

When investigating the structure–activity relationship of nitrogen fixation catalysts, it was discovered that nanostructured catalysts possess distinct advantages in the separation of photogenerated carriers and the adsorption and activation of nitrogen, owing to their unique physicochemical characteristics. These include a larger specific surface area, a greater number of defects and active sites, as well as shortened diffusion pathways. By modulating the nanostructures of photocatalysts, such as one-dimensional (1D) nanotubes, two-dimensional (2D) nanosheets, and three-dimensional (3D) hierarchical architectures, the photocatalytic nitrogen fixation activity can be augmented, paving the way for efficient nitrogen fertilizers. The research by Lin et al. also indicated that these hierarchical nanostructures can markedly enhance the catalytic nitrogen fixation capacity [156]. This enhancement is ascribed to the increased specific surface area, the augmented number of defects and active sites, and the diminished diffusion paths. These features collectively facilitate the dissociation of charges and the activation of N₂ molecules.

Designing nanostructured photocatalysts with optimal charge-separation efficiency is pivotal for enhancing nitrogen fixation performance. The optimization of nanostructures can reinforce the separation of photogenerated charges and boost nitrogen fixation efficiency. Concurrently, adjusting the nanostructures enables the design of efficient photocatalysts with optimal nitrogen activation sites, which are essential for the adsorption and activation of nitrogen molecules.

Moreover, the electronic structure of the catalyst can modulate the energy levels of the active sites and the stability of the reaction intermediate states, thereby influencing the rate and selectivity of the catalytic reaction. The adjustment of nanostructures can optimize the electronic structure and subsequently improve the catalyst's performance. Finally, the regulation of the nitrogen fixation reaction pathway induced by the hybridization of bimetallic atoms can effectively achieve high nitrogen fixation efficiency, presenting novel concepts for the design of new catalysts and the exploration of nitrogen fixation kinetics. Nanostructured materials remarkably enhance the efficacy of the catalytic system, especially in terms of stability during catalytic reactions, thanks to their unique architectural attributes.

**Fig. 12:**
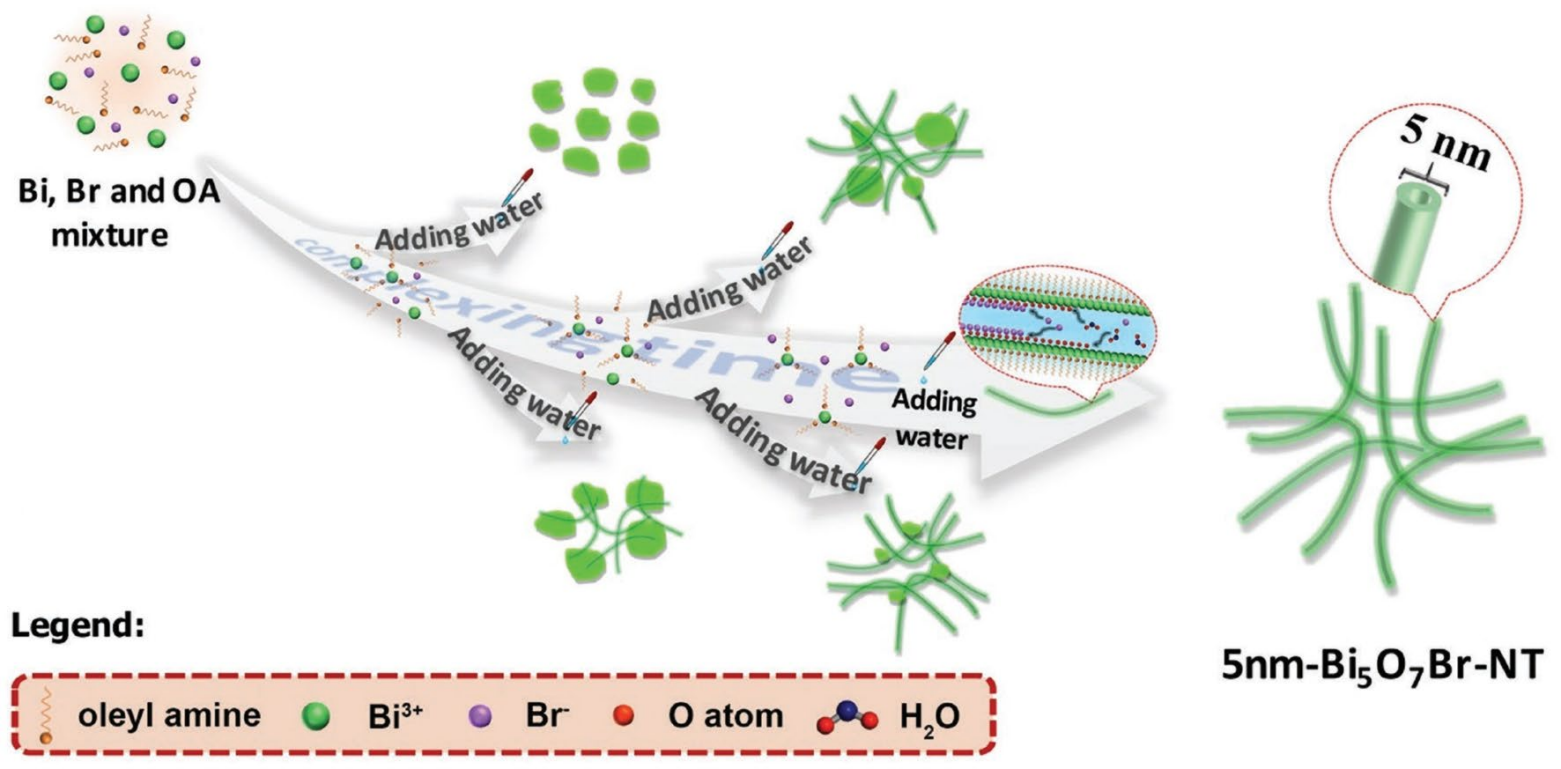
Schematic illustration of the preparation process of ultrafine Bi_5_O_7_Br nanotubes. Reproduced with permission [91]. Copyright 2017 Wiley–VCH

In a seminal study, Chen and co-authors have successfully synthesized self-assembled Bi_5_O_7_Br nanotubes through a novel approach involving the coordination of oleylamine with bismuth ions to create a precursor, which was subsequently subjected to a meticulous hydrolysis process (Fig. 12). The Bi_5_O_7_Br nanotubes possess significant advantages. Their uniform tubular morphology, with a diameter of about 5 nm and a length reaching tens of micrometers, greatly increases the specific surface area (up to 96.56 m^2^ g^–1^), providing more active sites for catalysis [91]. The uniform distribution of Bi, O, and Br elements along the nanotubes ensures the stability and consistency of the material. A high Bi/Br ratio endows the material with enhanced photostability and stronger visible light response. Particularly noteworthy are the abundant and photo-controllable reversible OVs on the surface; these vacancies can effectively capture and activate nitrogen under light, becoming key active sites. After the light is turned off, they can recapture oxygen atoms from water or oxygen to return to their original state. Compared to the previous BiOBr-001-OV nanosheets, this material has a higher concentration of oxygen vacancies under light. The synergistic effects of these structural, compositional, and surface properties make it perform excellently in photocatalytic nitrogen fixation reactions, providing important references for the design of high-efficiency photocatalysts. Subsequently, other researchers have refined the material using the same strategy and achieved remarkable results [157].

The construction of nanostructures faces numerous challenges in industrialization. From a scale-up perspective, the processes are complex and cumbersome, requiring high-standard equipment, stringent reaction conditions, and consistent batch-to-batch quality is difficult to ensure. Furthermore, the stability of nanostructures is suboptimal. Physically, the high surface energy leads to particle aggregation, reducing the specific surface area, a problem exacerbated during storage and transportation. Chemically, the high surface reactivity is only effective under ideal conditions; in industrial settings, active sites are prone to loss or deactivation.

To address these challenges, selecting an appropriate construction approach is crucial. We believe that self-assembly of materials offers significant advantages for scaled-up production [158]. Taking Prussian blue analogues as an example, this "bottom-up" fabrication method boasts high raw material utilization and precise synthesis. Renewable and biodegradable materials can be employed, promoting sustainable resource use. Their structures are stable, with some variants possessing self-healing capabilities, facilitating the construction of complex three-dimensional networks and hierarchical architectures. Moreover, this approach can utilize conventional reaction vessels, reducing equipment investment. The production process is simplified, leading to lower energy consumption and labor costs [159].

Regarding efficiency, the integration of automation and intelligent manufacturing enables high-throughput production. For instance, self-assembly reactions within microfluidic chips can rapidly synthesize large quantities of material. These processes are highly controllable and precise, allowing for the fine-tuning of active sites and the determination of optimal conditions, ensuring uniform product quality [160].

### Facets Engineering

**Fig. 13:**
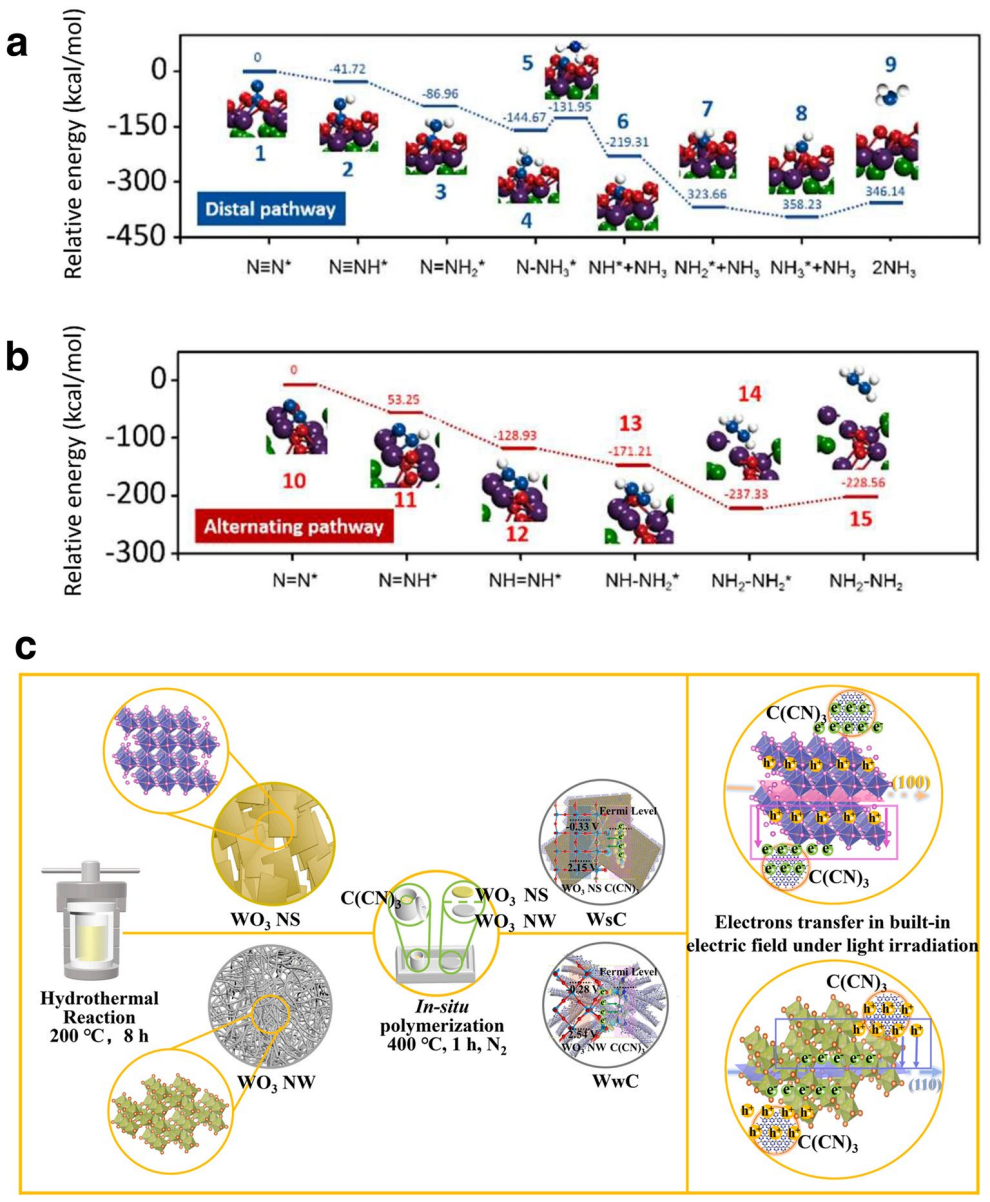
**a** OV-mediated N_2_ fixation on the (001) surface following a distal pathway; **b** OV-mediated N_2_ fixation on the (010) surface following an alternating pathway. Reproduced with permission [92]. Copyright 2016 Royal Society of Chemistry. **c** Synthesis of WsC and WwC composites, and the electrons transfer in built-in electric field under light irradiation. Reproduced with permission [166]. Copyright 2022 Elsevier

Surface properties are in-depth related to the multiple performances of crystalline materials. In general, distinctive properties of a crystalline material originate from the facets possessing different geometric and electronic structures [161]. The well-defined morphologies of crystal semiconductors on different facets bring about different properties for photocatalysis, photoelectricity, and so on. Therefore, methods and techniques for controllable synthesis of desired morphologies and crystal facets have received considerable attention [162]. In order to obtain the desired photocatalytic properties in clean-energy-related realm, it is crucial to select and control the exposed crystal facets [163, 164]. This approach capitalizes on the inter-facet charge-separation phenomenon to refine the generation and dissociation of charge carriers, while also enhancing the separation of photogenerated charges and the efficacy of catalytic reactions through controlled morphological adjustments and variations in surface band bending. As a group of pioneers, Zhang et al. began to study the influence of facet orientations on photocatalysis as early as 2012. The reaction mechanism about OVs-rich BiOX with different exposed facets was investigated. The photo-reactivities of BiOCl single-crystalline NSs with different exposed facets ((001) and (010)) were distinguishable [165]. BiOCl NSs with exposed (001) facet exhibited the high activity for pollutant degradation under UV illumination while the BiOCl NSs with exposed (010) facet showed the excellent activity for indirect photosensitized degradation of dyes with visible illumination. Another study reported the effects of different facets with OVs on the photocatalytic NRR pathway and final products [92]. For OVs-rich (001) facet, NH_3_ is generated through the distal pathway (Fig. 13a). For OVs-rich (010) facet, N_2_H_4_ is prone to be produced through the alternating pathway (Fig. 13b). The BiOX-based facet engineering to enhance the catalytic activity of nitrogen photo-fixation also includes the work from Chen’s group [93]. In addition, other semiconductor-based materials with photocatalytic NRR properties can also be engineered to forge efficiency/selectivity-enhanced catalysts.

Furthermore, facet engineering facilitates the targeted deposition of co-catalysts on specific facets, thereby amplifying the photocatalytic rate. Herein, Zou et al. have introduced an innovative approach to enhance electron pump facet engineering by integrating the organic half-metal C(CN)_3_ onto the (100) and (110) facets of WO_3_ nanosheets and nanowires (Fig. 13c). In the case of WO_3_ nanosheet-based composites (WsC), the photoinduced electrons are directly transferred to the active sites of C(CN)_3_, which functions as the inaugural organic co-catalyst for nitrogen fixation [166].

For multi-component semiconductor-based materials, facets engineering for one of the components is an easy means to improve photocatalytic performance. For example, facet engineering of M-S type materials can select metal or semiconductor separately. The exposed facets of metal nanoparticles (NPs) or semiconductors may have an opportunity to influence the overall performance of photocatalysts [167]. There are numerous applications of this strategy in other fields, which is worthy of reference in photocatalytic nitrogen fixation [168–171].

Facets engineering, as a means to optimize photocatalysts, presents unique opportunities and challenges in large-scale industrialization. On the one hand, its strengths lie in its high specificity, which allows for the precise exposure of highly active and selective crystal planes according to the characteristics of photocatalytic reactions, thereby significantly enhancing the efficiency and selectivity of photocatalytic reactions and optimizing the separation efficiency of photogenerated electron–hole pairs. Moreover, the theoretical research in this area is well-developed, with mature theoretical foundations and computational methods guiding practical applications, thereby increasing the precision of optimization. Additionally, photocatalysts optimized through crystallographic plane engineering exhibit stable crystal structures, demonstrating excellent activity and stability in long-term photocatalytic reactions.

On the other hand, facets engineering also faces significant challenges. In the preparation phase, the precision required for controlling crystal growth processes and conditions is extremely high. For instance, when using hydrothermal methods to synthesize nanocrystals with specific crystal planes, minor fluctuations in conditions can easily alter the exposure and morphology of the crystal planes. From a cost perspective, the preparation costs are often high due to expensive raw materials, special templates, or complex post-processing steps. Furthermore, current research is predominantly focused on a few typical materials [172], and the applicability to other photocatalysts remains to be explored, limiting its widespread industrial application due to restricted generalizability.

### Support Selecting

Support materials are instrumental in the photocatalytic process, markedly augmenting the catalytic efficiency and bolstering the structural integrity of photocatalysts, which in turn protracts their operational lifespan. These materials streamline the processes of catalyst separation and recycling, thereby facilitating their sustainable reuse. Employing specialized structural configurations, support materials augment the capacity for light capture, enhancing the absorption of photons.

WO_3_ has been integrated as a substrate material with other semiconductor photocatalysts, effectively mitigating the limitations of TiO_2_ in the context of visible light utilization while concurrently enhancing its photocatalytic efficacy. Characterized by a reduced bandgap energy of 2.8 eV, WO_3_ is capable of absorbing a more extensive spectrum of visible light. Furthermore, it exhibits superior resistance to photo-corrosion and chemical stability, which are pivotal attributes for its application in photocatalytic processes [173]. Besides, a spectrum of materials has been recognized for their substantial enhancement of photocatalytic performance. Bismuth oxyhalides (BiOX, with X denoting Cl, Br, or I) are distinguished by their exceptional photocatalytic activity and stability, attributed to their distinctive layered architecture and well-suited bandgap [174]. The exploration of two-dimensional materials, such as graphene and transition metal dichalcogenides (e.g., MoS_2_), as support materials is also burgeoning, given their capacity to augment photocatalytic efficiency [156]. Three-dimensional ordered macro-porous (3DOM) materials have garnered attention for their extensive surface area and plethora of active sites, which are conducive to the capture of photons and the segregation of photogenerated charge carriers, thus ameliorating photocatalytic efficacy [175]. The advent of oxygen vacancy-enriched tungsten oxide (WO_3-x_) nanowires has elucidated that the spin polarization induced by OVs can markedly elevate the photocatalytic capabilities of the material [176]. Cadmium sulfide (CdS), classified as an n-Type semiconductor, has garnered significant interest in photocatalysis due to its narrow bandgap and elevated negative conduction band potential, rendering it particularly adept for visible-light-induced NRR [117]. Cobalt oxide (Co_3_O_4_) is also gaining prominence as a photocatalyst, with its photocatalytic properties being notably augmented through a suite of modification techniques, underscoring the multifaceted strategies essential for optimizing the photocatalytic materials for applications in energy conversion and environmental remediation [177].

**Fig. 14:**
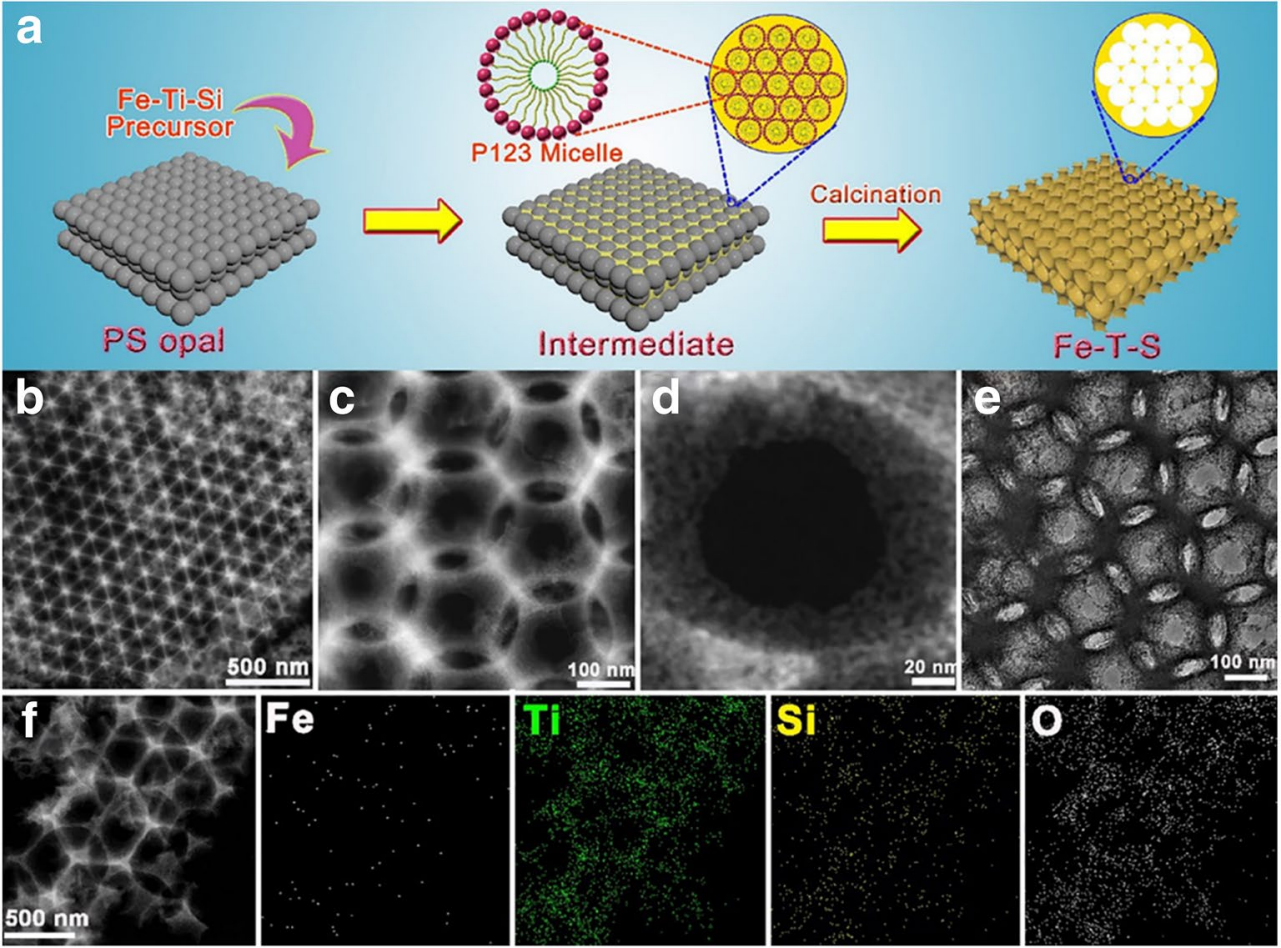
**a** Schematic illustration of the synthesis process for Fe-T-S. **b − d** STEM images of Fe (2%)-T-S at different magnifications. **e** TEM image of Fe (2%)-T-S. **f** STEM image of Fe (2%)-T-S and corresponding elemental mapping of Fe, Ti, Si, and O. Reproduced with permission [178]. Copyright 2021, American Chemical Society

Taking the work of Zhang et al. as an example, the Fe-T-S catalyst was prepared using the evaporation-induced self-assembly (EISA) method, with polystyrene opal as the macroporous hard template and P123 micelles as the mesoporous soft template [178]. The Fe-Ti-Si precursor solution was injected into the PS interstices, followed by hydrolysis and sintering to remove the templates and form a porous structure. By varying the amount of FeCl_3_·6H_2_O added, samples with different Fe doping levels were prepared. Interestingly, Fe-T-S possesses a uniform and ordered mesoporous structure, with Fe atoms doped into the TiO_2_-SiO_2_ framework in the form of single atoms, primarily existing as Fe (III). Doping did not significantly increase the concentration of OVs and N_2_ chemical adsorption, but it did cause a redshift in the absorption edge, enhancing the absorption of visible light. In this study, SiO_2_ played multiple key roles in the structure of the catalyst. Firstly, it formed a composite structure with TiO_2_, providing a robust skeletal support for the catalyst, ensuring the stability of the catalyst's overall shape and pore structure during the reaction process, preventing the aggregation or structural collapse of the catalyst, which is crucial for maintaining the catalyst's long-term performance and catalytic activity. Secondly, SiO_2_ significantly affected the catalyst's pore structure, working synergistically with TiO_2_ to form mesopores and macropores of specific sizes, facilitating the diffusion and transport of reactants and products, increasing the contact opportunities between reactants and active sites, thereby improving the efficiency of the catalytic reaction. Additionally, some Fe atoms were doped into the SiO_2_ framework, working together with Fe doped in TiO_2_, affecting the catalyst's electronic structure and distribution of active sites. This diverse doping and composite structure may alter the catalyst's adsorption properties for reactants and electron transfer pathways, which helps to improve the adsorption and activation efficiency of nitrogen in photocatalytic reactions, as well as promote the progress of key steps such as water oxidation and nitrogen hydrogenation, thereby enhancing the overall photocatalytic nitrogen fixation performance (Fig. 14).

Insulators also play a significant role in photocatalytic nitrogen fixation, as they can serve as support materials or synergize with semiconductors to optimize light absorption, charge separation, and nitrogen activation. Materials such as aluminum oxide (Al_2_O_3_) and silicon dioxide (SiO_2_) are commonly used as insulating support materials to improve the dispersion of active photocatalytic sites. Al_2_O_3_ isolates active sites to reduce charge recombination, while SiO_2_ enhances surface area and minimizes side reactions. For instance, the g-C_3_N_4_/SiO_2_ hybrid system demonstrates improved photocatalytic nitrogen fixation performance due to better dispersion of g-C_3_N_4_ and enhanced nitrogen adsorption on SiO_2_, with SiO_2_'s light scattering properties promoting higher catalytic efficiency. Insulators also synergize with active materials; for example, hexagonal boron nitride (h-BN) combined with TiO_2_ facilitates electron migration and nitrogen activation, with h-BN adsorbing nitrogen molecules to provide polarization and activation sites. In ZnO/SrTiO_3_ composite materials, the insulating nature of SrTiO_3_ extends the lifetime of photogenerated electrons, enhancing nitrogen reduction efficiency. Insulators with oxygen vacancies, such as CeO_2_ and ZrO_2_, when combined with semiconductors, act as electron reservoirs and nitrogen adsorption sites, enhancing catalytic activity. Furthermore, the curvature and surface effects of insulators can also promote their role in improving nitrogen fixation performance. For example, ZrO_2_ with engineered surface oxygen vacancies, when paired with semiconductors, synergizes with the semiconductor's light absorption capabilities to maximize charge separation and stabilize reaction intermediates. Layers of Al_2_O_3_ combined with Ti_3_C_2_ MXenes maintain effective electron transfer and suppress charge recombination.

Lv et al. have delineated the advancements in the realm of insulator-associated photocatalysts [179]. Within the photocatalytic process, insulators can perform multiple pivotal roles: (i) they can act as co-catalysts to expedite the transfer of charge carriers from semiconductor photocatalysts, (ii) they can serve as robust support matrices to disperse semiconductor photocatalysts and/or enhance the substrate's adsorptive capacity, and (iii) they can function as protective barrier layers to mitigate charge carrier recombination and avert photocatalytic degradation. Furthermore, the activation of insulators through the modulation of material surface properties has been identified as a strategy to foster charge carrier separation and augment light absorption. Such strategies encompass: (i) the introduction of oxygen vacancies, (ii) the incorporation of metal impurities, and (iii) the exploitation of the localized surface plasmon resonance (LSPR) phenomenon.

**Fig. 15:**
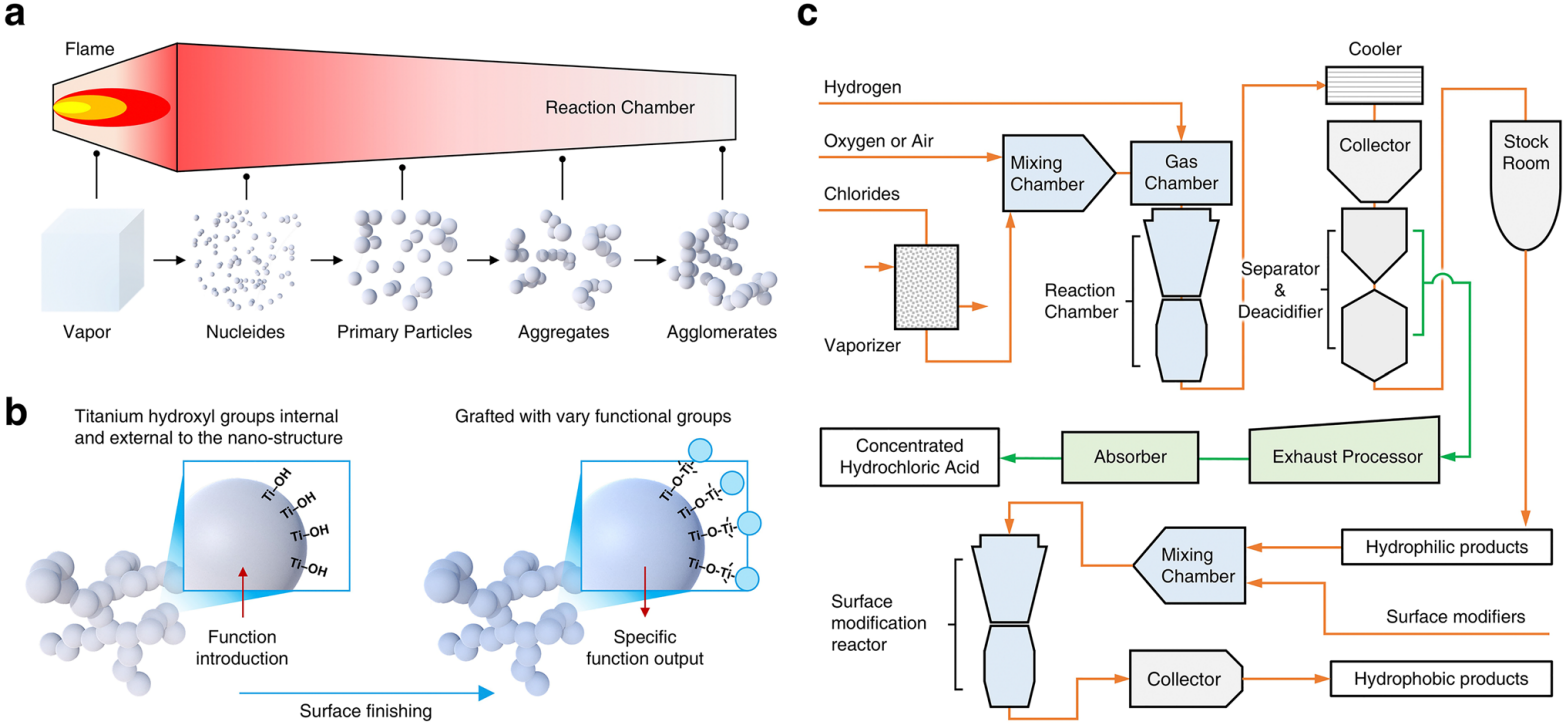
**a** Scheme of gas-phase metal oxide synthesis based on the high-temperature nucleation and evolution mechanism. The total reaction steps include (i) reaction and nucleation; (ii) nuclides collision and primary particles formation; (iii) primary particles collision and aggregates formation; (iv) aggregates collision and agglomerates formation. **b** Scheme of fumed titanium dioxide functionalization modification based on titanium hydroxyl groups. **c** Scheme of fumed non-noble metal oxide nano-sized powder substrate materials production process flow chart

To meet the demands of scale-up production of photocatalyst substrates, flame synthesis methods demonstrate significant advantages. In terms of process efficiency, the reaction speed is extremely fast. For instance, a non-equilibrium flame aerosol synthesis technique employed by Dun et al. can convert multiple elements into metastable materials and form them into nanoscale structures in approximately 200 ms. The production cycle is short, eliminating the need for the prolonged aging and drying required in sol–gel methods, enabling rapid transformation from raw materials to finished products and facilitating continuous production [180]. Regarding product characteristics, the resulting products are highly pure and can integrate multiple modification steps. Taking the preparation of ultrafine fumed TiO_2_ by gas-phase hydrogen flame hydrolysis as an example, the principle involves the hydrolysis and oxidation of titanium tetrachloride (TiCl_4_) in a mixed atmosphere of H_2_ and O_2_, followed by rapid nucleation. As the reaction progresses, these nuclei grow larger and more structurally stable through collision and agglomeration [181]. Temperature and atmosphere control can promote surface and grain boundary diffusion, regulate particle size and connectivity. A reducing atmosphere can induce surface defects, increasing the density of charge carriers and adsorption sites, thereby enhancing photocatalytic activity (Fig. 15a) [182]. The presence of titanium hydroxyl groups allows for surface modification, such as coating to construct core–shell structures, further preventing agglomeration or enhancing catalytic performance (Fig. 15b). From a cost control perspective, the equipment is relatively simple and the operation is straightforward, with no complex post-processing required. The high-temperature flame ensures complete reaction of the raw materials, resulting in high material utilization and minimal waste, thus saving costs. Moreover, exhaust gases can be easily treated by absorption and separation using alkaline or acidic solutions and can be sold as by-products (Fig. 15c). This technology is almost universally applicable to all non-noble metal elements used in the construction of photocatalysts, and substrates such as TiO_2_, ZnO, and Al_2_O_3_, which are excellent candidates, have already been produced on a large scale.

### Heteroatom Doping

#### Metal Doping

**Fig. 16:**
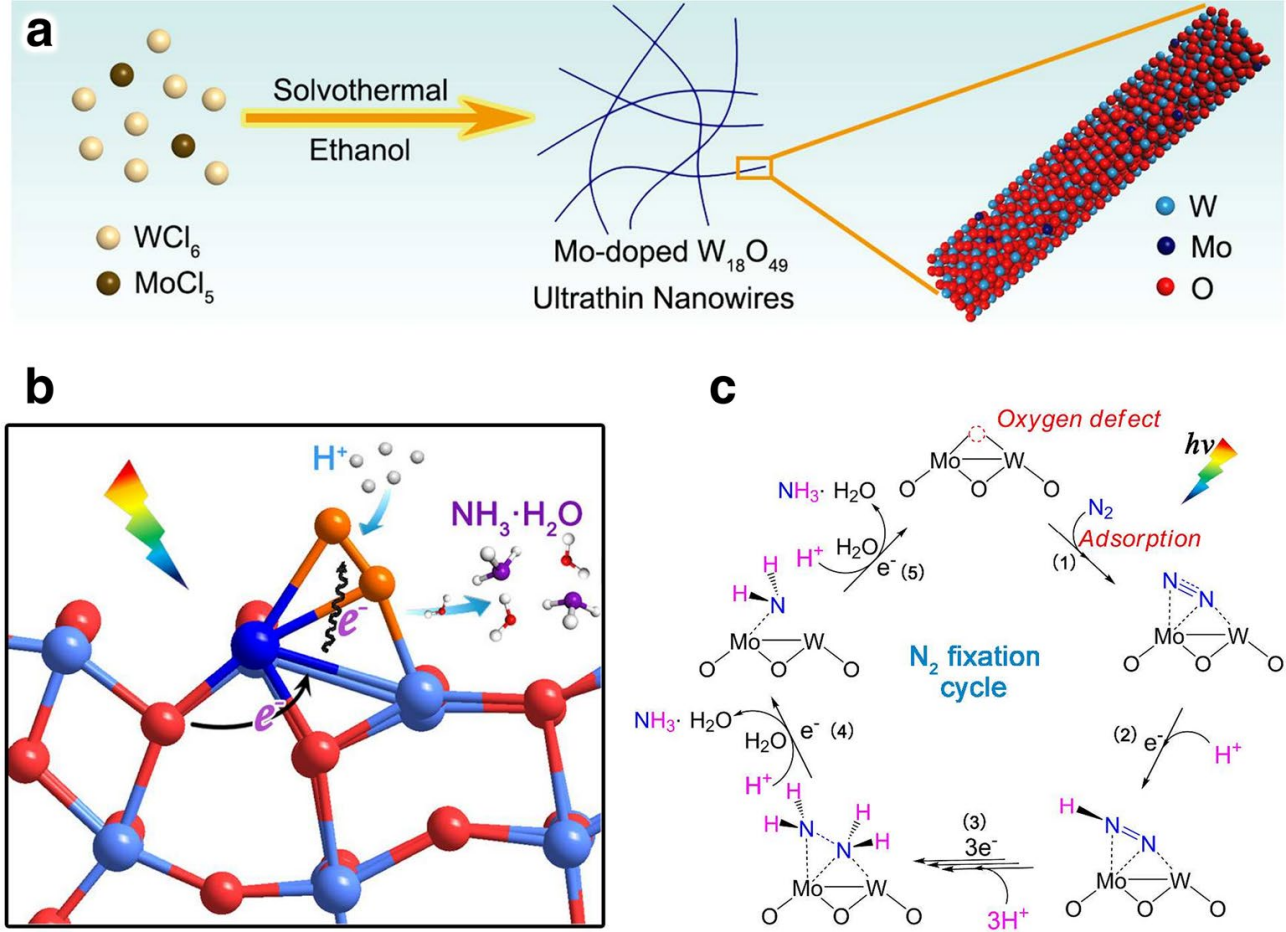
**a** Schematic illustration for the synthesis of Mo-doped W_18_O_49_ ultrathin nanowires (UTNWs). **b** Schematic illustration for the photocatalytic PCET process for N_2_ reduction in the case of Mo-doped W_18_O_49_. **c** Proposed reaction pathway for photocatalytic N_2_ fixation to NH_3_·H_2_O using MWO-1 UTNWs as a catalyst. Reproduced with permission [106]. Copyright 2018 American Chemical Society

Doping as a fundamental strategy has been widely applied in catalyst design. In 1977, a pioneering work reported that the main NRR catalytic activity was contributed by rutile TiO_2_, Fe doping could improve the yield rate of NH_3_ and N_2_H_4_ [94]. Then, better performance and higher yield rate were obtained by the metal-vapor-synthesized Fe-doped TiO_2_ [95]. The promotion of Fe on photocatalysis was proved in mechanism by follow-up study; this contribution comes from the presence of dispersed Fe^3+^ [96]. The electrons can be trapped by Fe^3+^ to prevent the recombination with holes. Meanwhile, holes on the surface facilitate the formation of hydroxyl radicals and combine them with N_2_ to form [N_2_–OH]*. Wang et al. have made a breakthrough in performance for this material in 2014 [97]. The midway generated OVs associated with the doped Fe further promote the H_2_O adsorption and –OH radical formation. Moreover, numerous metals (Mg, Cr, V, Ce, Ru, Rh, Os, Pd, Pt, and so on) have also been doped into TiO_2_ to improve photocatalytic NRR performance [98–100]. The generated metal–semiconductor (M-S) junction and electric field at the interface of metal/TiO_2_ promote the isolation of electrons and holes, which improve the photocatalytic performance [42]. Thereinto, Ru is recognized as the most preferred noble metal with good properties [101]. Furthermore, other M-S-type photocatalysts have also been designed to improve catalytic activity. TiO_2_ film modified with RuCl_3_ possesses n-scheme semiconductor behavior and enable the photo-fixation of N_2_ to NH_3_ and nitrate in the presence of C_2_H_5_OH or humic acid [102]. CdS-based doping such as Pt/CdS [103], and carbon-based doping such as Fe-doped g-C_3_N_4_, Similarly, some rare M-S type semiconductors (Pt/GaP, Pt/ZnO) have also been designed [104, 105]. Moreover, a significant work reported by Xiong et al. demonstrated an ingenious method of regulating N_2_ activation for nitrogen photo-fixation [106]. The W_18_O_49_ ultrafine nanowires have a uniform and dispersed structure with high specific surface area (Fig. 16a). The active coordinatively unsaturated sites in W_18_O_49_ are beneficial to N_2_ adsorption and electron transfer (Fig. 16b). The low-valence Mo doping promotes the activation and dissociation of N_2_. Mo doping brings two contributions: (i) The N_2_ polarization and electron transfer promotion make it possible to cleave N≡N bond. (ii) The center of the defect band is elevated to Femi’s level, and the energy of photoexcited electrons is retained for NRR (Fig. 16c). The innovative design approaches and mechanism explanation can broad our horizons for next-generation development of semiconductor photocatalysts.

Some doping methods and defect construction methods suitable for scale-up production are universal. Despite them, ion exchange is one of the effective methods suitable for heteroatom doping. This approach can introduce specific ions into the lattice of photocatalysts [183]. Even in organic semiconductors, doping can be performed without relying on strong oxidizing or reducing agents (dopants) [184]. In addition, chemical vapor deposition (CVD) and atomic layer deposition (ALD) are also commonly used industrial doping techniques [185]. By decomposing gaseous dopant sources under high-temperature conditions and reacting them with the surface of photocatalysts, specific elements can be functionally introduced. These two methods generally allow precise control of doping amounts and positions, making them very suitable for the preparation of panel film photocatalytic systems.

#### Metal-Free Doping

**Fig. 17:**
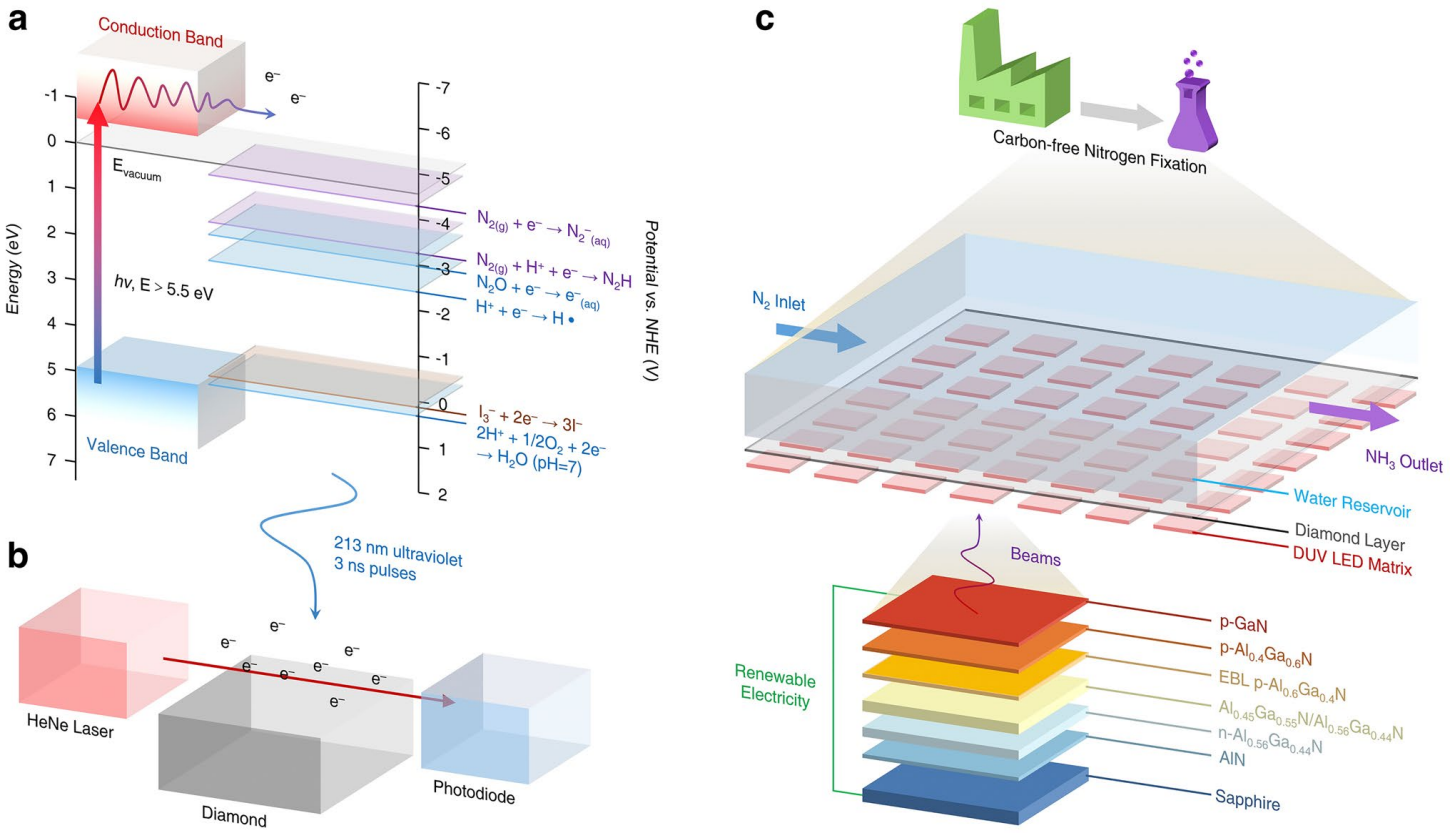
**a** Scheme of the electronic energy-level of diamond. **b** Scheme of transient light absorption measurement of solvated electrons produced by photoemission from the diamond surface. **c** Scheme of a proposed carbon-free strategy for photocatalytic nitrogen fixation combining diamond layers with DUV LED matrix

Metal-free doping often improves photocatalytic NRR performance by adjusting the chemisorption of N_2_. However, there are not enough samples for this type of catalyst. WO_3_·H_2_O, which has been confirmed to be photoactive, has been used to construct metal-free doping semiconductor catalyst [186]. The microwave-assisted synthesis of carbon-tungstic-acid hybrids (HWO-C) with enhanced abilities of N_2_ activation and photoinduced protonation are reported by Sun et al. [107]. The modification of introduced C in material proves that it plays a decisive role in N_2_ chemisorption. The synergistic N_2_ chemisorption by C and W enhances the superficial activity of material surface and then increases the NH_3_ yield rate. Moreover, diamond as a unitary semiconductor with a bandgap of 5.5 eV can acquire high electrical conductivity by using metal-free doping [108, 187]. Thanks to the unique property of diamond called negative electron affinity (NEA), when a diamond is illuminated with high-energy light source (*hv* > 5.5 eV), the excited electrons that ought to go into the CB can diffuse to the surface of the material and be directly emitted into the vacuum. With the aid of this phenomenon, Hamers et al. overcome the various limitations of N_2_ reduction by directlyintroducing solvated electrons into the reactant fluid [108]. The huge bandgap of H-terminated diamond makes it easy to break through the energy barrier required for nitrogen reduction (Fig. 17a). In N_2_-saturated H_2_O, the emitted electrons will interact with H_2_O or protons to form H atoms and then form N_2_H. Through this pathway, NH_3_ will be eventually formed (Fig. 17b). The diamond-based semiconductor catalysts with metal-free doping are based on a fundamentally different catalytic mechanism in the nitrogen photo-fixation.

Owing to these factors, we contend that the utilization of diamond in conjunction with deep ultraviolet (DUV) light for photocatalytic nitrogen fixation presents a viable and innovative approach. As depicted in Fig. 17c, leveraging the previously discussed catalytic mechanisms, we have conceptualized a potential carbon-free strategy for the amplification of photocatalytic nitrogen fixation, predicated on the integration of diamond thin films with DUV LED arrays. Diamond's inherent attributes, such as its remarkable hardness, chemical inertness, high thermal conductivity, and wide bandgap, endow it with the capacity to withstand corrosive environments, expedite heat dissipation, and capture high-energy photons, thereby supplying the requisite energy for nitrogen activation and conversion within photocatalytic systems. The DUV LEDs are capable of emitting deep ultraviolet light at wavelengths precisely aligned with the photocatalytic nitrogen fixation reaction requirements. Characterized by its high energy density, ease of parameter modulation, and superior stability (over 10,000 h), DUV light facilitates the accurate activation of catalysts, concomitantly reducing energy expenditure and equipment costs. The synergistic interaction between diamond and DUV light enhances the generation and separation of photogenerated charge carriers, augmenting the availability of active sites for the reaction. Once connected to the photovoltaic (PV) grid, this scale-up strategy may offer an excellent return on investment, owing to the long lifespan of all components.

At present, the prospects for cost reduction of industrial diamonds and DUV LEDs are both very optimistic. In terms of technological innovation, industrial diamonds benefit from the popularization of microwave plasma chemical vapor deposition (MPCVD) equipment, while DUV LEDs have seen significant improvements in production efficiency and product quality due to breakthroughs in ultrathin AlN templates and silicon substrate growth technologies by institutions such as the Guangdong Academy of Sciences Semiconductor Research Institute and the RIKEN in Japan [188, 189]. Regarding economies of scale, the rapid growth of the global market has prompted related enterprises to expand their production scale, achieving economies of scale in raw material procurement and other aspects, thereby further reducing costs. Additionally, the formation of industrial clusters allows enterprises to share resources and optimize supply chains, further lowering production costs. These factors collectively drive the cost reduction of industrial diamonds and DUV LEDs, providing broader application prospects in fields such as photocatalytic nitrogen fixation.

### Single-Atom Site Creating

Like its advantages in electrocatalysis [190], single-atom catalysts (SACs) exhibit significant superiority in the field of photocatalysis, primarily reflected in their high atomic utilization efficiency, which maximizes the use of metal atoms. Through their unique unsaturated coordination environment and electronic properties, SACs achieve absorption of a broader spectral range and efficient charge transfer. They also significantly enhance photocatalytic efficiency by strengthening light capture capabilities and improving surface reaction kinetics. Moreover, the stability and corrosion resistance of SACs are superior to those of traditional catalysts, thanks to the strong interactions between metal atoms and the support matrix. The selective control capability of SACs allows for more precise regulation of the reaction process to produce specific products. By altering the support or types of metal atoms, the structure and performance of SACs can be adjusted to optimize catalytic activity.

Li et al. have proposed a new model material that uses graphene embedded with FeN_3_ for nitrogen transamination through spin-polarized activated nitrogen, offering new ideas for industrial nitrogen fixation and ammonia production under ambient conditions [191]. The team has developed the inaugural metal-free single-atom catalyst, where boron atoms are dispersed on a graphite-nitrogen framework (B/g-C_3_N_4_). Utilizing sp3-hybridized boron atoms to activate nitrogen molecules, the catalyst markedly enhances the absorption of visible light, thereby facilitating the efficient reduction of nitrogen to ammonia at an exceptionally low onset potential of 0.20 V under visible-light irradiation. This catalyst exhibits not only high catalytic efficiency but also superior stability, coupled with the advantages of cost-effectiveness and facile synthetic accessibility [192].

**Fig. 18:**
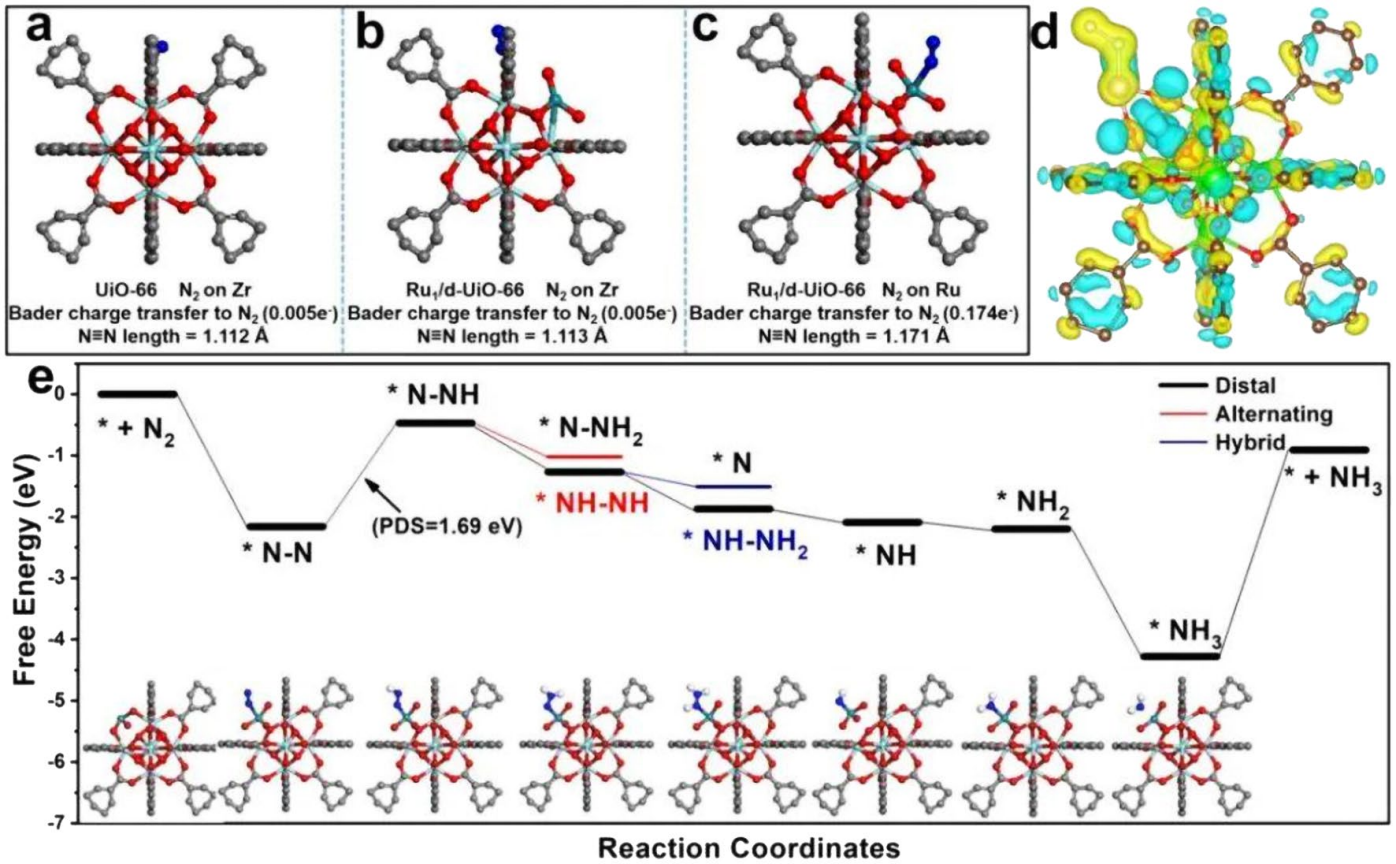
**a-c** The optimal adsorption configurations of nitrogen molecular on catalyst. **d** Electronic distribution on catalyst. **e** Free energy diagrams of catalyst through three distinct reaction pathways. Reproduced with permission [109]. Copyright 2023 Wiley–VCH

Meng et al. have proposed a strategy of photochemical etching to construct defects in situ and precisely induce the loading of metal single atoms. This strategy has enabled the high dispersion construction of Ru metal single atoms on metal–organic framework materials such as UiO-66 (Zr), significantly enhancing the activity of photocatalytic nitrogen fixation for ammonia synthesis [109]. Concurrently, the single-atom Ru catalysts exhibit a robust electron–metal–support interaction with the substrate, which facilitates the rapid transfer of photogenerated charge carriers between the metal single atoms and the metal–organic frameworks (MOF). This interaction significantly enhances the photocatalytic nitrogen fixation activity, achieving an impressive increase in the ammonia yield rate from 4.57 to 53.28 μmol g^–1^ h^–1^ under ambient temperature and pressure. DFT computations have been meticulously executed to dissect the electron-mediation-induced spin polarization (EMSI) phenomenon within the material in question (Fig. 18) and to expound upon the ramifications of the Ru single-atom immobilization upon UIO-66's local electronic architecture and its consequential influence on the NRR trajectory. The analytical outcomes underscore that the *d*-orbital electrons of the Ru atom are transferred to the π* antibonding orbitals of the N_2_ molecule, which catalyzes the activation of the N≡N triple bond. Within the Ru-anchored UIO-66 catalyst, the distal hydrogenation mechanism is adjudged to be more thermodynamically favorable in comparison with its alternating counterpart. Specifically, the initial hydrogenation stage involving the N_2_ molecule (*N_2_ → *NNH) is delineated as the kinetic bottleneck of the entire catalytic process.

Significant strides have been made in the industrialization of single-atom catalysts. For instance, Ding and colleagues have developed a multi-phase single-atom catalyst that has enabled the large-scale synthesis of n-propanol [193]. Wang and co-workers have created the In/Rh@S-1 catalyst, which is applicable for the production of light olefins and maintains stable performance under near-industrial conditions [194]. It is evident that the focus on stable and efficient single-atom catalysts currently lies in the anchoring and utilization of noble metal elements. In contrast, the industrialization path for single-atom photocatalysts remains underreported, clearly facing numerous challenges. Synthetically, achieving both stable dispersion of single atoms and increased loading is no easy feat. The majority of existing synthesis methods are complex and fail to ensure batch-to-batch consistency, making large-scale production difficult. In terms of performance, active sites are prone to poisoning by reaction intermediates or by-products, exhibit poor resistance to impurities and solvent interference, and require stringent reaction conditions; any fluctuations can lead to unstable performance. Regarding catalyst lifetime, factors such as the reaction environment and mechanical stress can compromise stability, leading to the detachment, agglomeration, or structural alteration of single atoms, and once deactivated, regeneration is challenging and often fails to restore initial performance. These factors collectively impede the industrialization of single-atom catalysts.

We posit that ball milling is a promising approach for the industrialization of single-atom photocatalysts. From a process standpoint, ball milling is straightforward, leveraging the rotation or vibration of a ball mill jar to facilitate mixing, refinement, and reaction through the collision, friction, and shearing of ball mill balls with the raw materials. The process is easy to master, conducive to personnel training, and highly scalable. By increasing the volume of the ball mill jar, the number of ball mill balls, and adjusting the milling time, production can be effortlessly scaled from milligram and gram levels in the laboratory to kilogram and even ton levels [195]. In terms of product quality, ball milling ensures the uniform dispersion of single atoms. The mechanical energy employed gradually breaks down and disperses the metallic particles, allowing atoms to evenly adhere to the support, thereby enhancing activity and selectivity, reducing agglomeration, and ensuring stability and longevity. Moreover, the stable and controllable process parameters guarantee uniform product quality. Cost-wise, the simplicity of the equipment and the primary consumable being the ball mill jar result in significantly lower investment costs compared to ALD and CVD, thereby reducing the industrialization threshold and initial risks. The high atom utilization rate and the absence of solvents or chemical reagents also minimize metal waste and raw material consumption. This method is notably environmentally friendly, as it typically operates without solvents, thus avoiding solvent volatilization and pollution. The predominance of physical mechanical actions also results in fewer by-products, easing the burden of subsequent treatment. In terms of energy efficiency, the process operates at room temperature, eliminating the need for high-temperature heating or calcination. The ball milling time can be flexibly adjusted according to production needs, resulting in shorter processing times and significant energy savings, which substantially reduce production costs.

### Multi-Component Assembling

As technology advances, better photocatalytic performance can be achieved by assembling semiconductors with functional materials. The functional materials here include conductors and semiconductors, which are classified according to their element composition and material type.

#### Conducting Polymer

It is a cost-effective strategy to enhance performance by combing well-designed catalysts with compatible conductor. The various components appearing in the same system may provide good conductivity, catalytic activity or other characteristics. Hoshino et al. designed a composite photocatalytic system which was comprised of a TiO_2_/poly(3-methylthiophene) (P3MeT) heterostructure [196]. Plentiful photogenerated carriers convert N_2_ to NH_3_ at the TiO_2_/polymer interface under ambient conditions [110]. The product synthesized under illumination is needle-like NH_4_^+^ClO_4_^−^ crystal. It should be added that the comparable NH_3_ yield rate can be inferred from the comparison with some other reported methods that the separation of photogenerated carriers on the TiO_2_/polymer interface, and the existence of P3MeT inhibits the accumulation of NH_3_ from TiO_2_ counterpart [197]. There are other follow-up work, such as the utilization of amorphous TiO_2_ [111]. These studies promote the development of inorganic/organic heterostructure semiconductor photocatalysts.

#### Conducting Metal Oxides

More attention has been paid to the combination of multiple metal oxides. Most of the transition metal oxides are semiconductors. The early research object was the combination of iron oxide (FeO_x_) and TiO_2_. In 1977, the Fe_2_O_3_/TiO_2_ binding material was obtained by heating the anatase-type TiO_2_ containing iron sulfide [94]. When the catalytic device is irradiated under UV light, traces of NH_3_ and N_2_H_4_ can be detected. When the iron oxide content in the component is 0.2%, the NH_3_ yield rate reaches the maximum value. The optimized Al_2_O_3_-supported Fe_2_O_3_/TiO_2_ hybrid was prepared for enhancing NH_3_ yield rate [112, 113]. The promoted NH_3_ yield rate was obtained by using gas–solid fluidized bed reactors with the adjustment of metal oxide contents. The formed Fe_3_O_4_/α-Fe_2_O_3_ heterostructure by in situ partial reduction is also an early-investigated composite for nitrogen photo-fixation under ambient conditions [114]. The catalytic products of this material included both H_2_ and NH_3_. The yield rate of H_2_ is about four times that of NH_3_.It can be seen that the catalytic process is still accompanied by the strong competition of HER, but for a cost-effective semiconductor-based composite, the selectivity of about 20% is fairly enough.

**Fig. 19:**
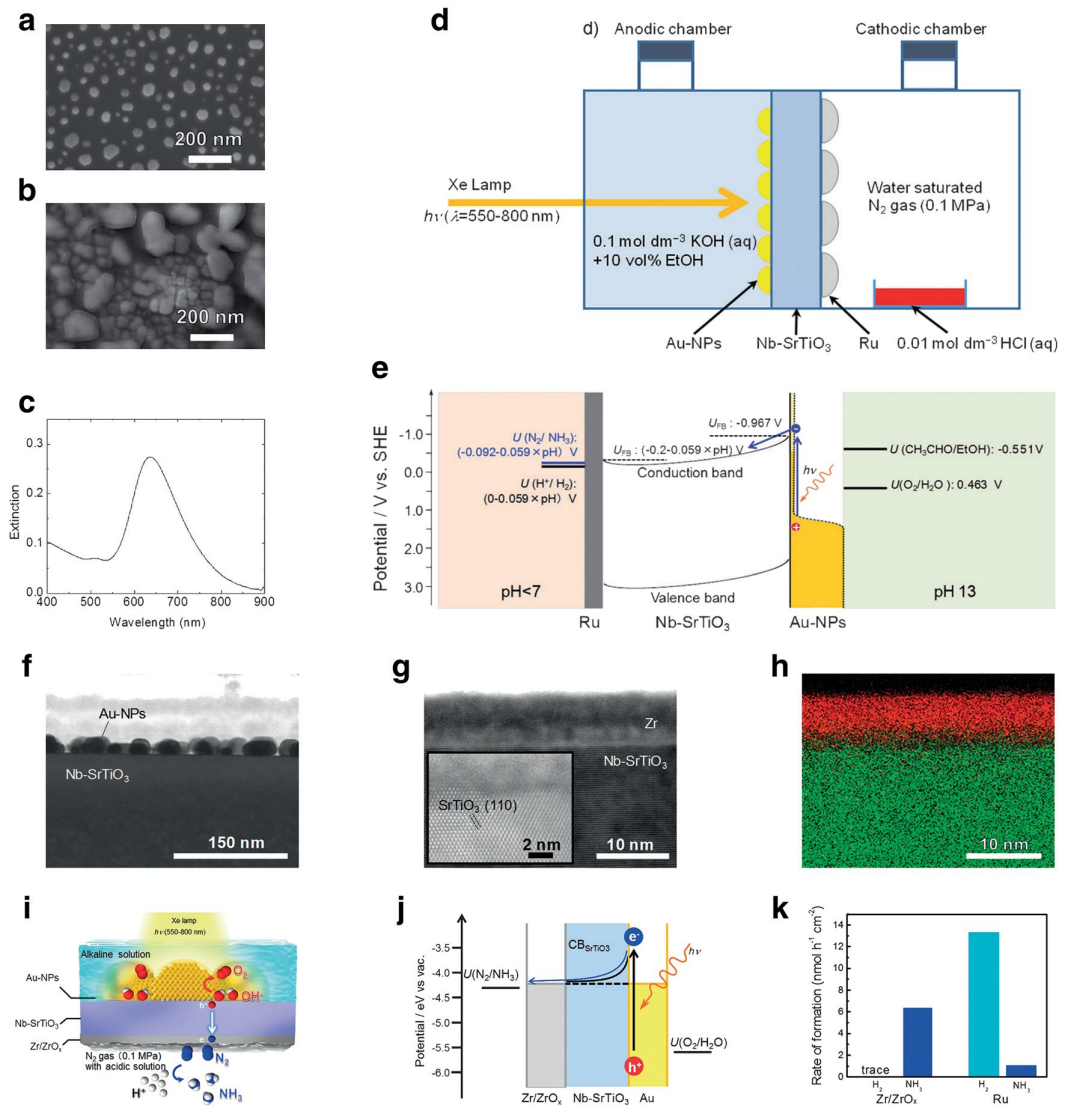
**a** SEM image of Au NPs on Nb-SrTiO_3_, prepared using the annealing method. **b** SEM image of Ru on Nb-SrTiO_3_, prepared using the electron beam evaporation method. **c** Extinction spectrum of the Au NPs on Nb-SrTiO_3_. **d** A schematic illustration of the NH_3_ synthesis device using a Nb-SrTiO_3_ photoelectrode loaded with Au NPs. **e** An energy diagram of the plasmon-induced ammonia photosynthesis system using a SrTiO_3_ substrate loaded with Au NPs. Cross-sectional view of the BF-STEM image of the **f** Au-NPs/Nb-SrTiO_3_ interface and the **g** Zr film deposited onto the single-crystalline Nb-SrTiO_3_with corresponding elemental mapping **h** by EDS spectrum (Zr: red, Ti: green). **i** Layout of the NH_3_ synthesis device using Au-NPs/Nb-SrTiO_3_/Zr/ZrO_x_. **j** Energy diagram of the plasmon-induced NH_3_ photosynthesis system using Au-NPs/Nb-SrTiO_3_/Zr/ZrO_x_. **k** Reaction rate of each product in the cathodic chamber over Au-NPs/Nb-SrTiO_3_/Zr/ZrOx and Au-NPs/ Nb-SrTiO_3_/Ru.Reproducedwith permission [137, 138]. Copyrights 2014 and 2016 Wiley–VCH

The catalysts applied in PEC nitrogen fixation are basically composites. Therefore, the catalysts used in these studies are classified as semiconductor-based multi-component assembly. The widespread utilization of PEC catalysis stems from the discovery of PEC water splitting in 1972 by Fujishima and Honda [198]. An early pioneering work performed a PEC cell for nitrogen fixation using p-GaP semiconductor materials [131]. The electrolyte was prepared by co-dissolving the catalyst precursor titanium tetraisopropanolate and aluminum trichloride (AlCl_3_) in ethylene glycol dimethyl. The Al metal was used as a photoanode, while cathodic photoelectrode contained GaP as light absorber and photocatalytic reactor to realize the photoreduction of N_2_. The disadvantages of this study are the high toxicity and environmental pollution. Years later, materials derived from titanates were used to attempt to realize nitrogen photo-fixation under mild conditions. With the deepening of research, more photocatalytic semiconductor-based composite materials based on using similar crystalline structures have been developed, such as barium titanate (BaTiO_3_), OV-enriched BaTiO_3_ (BaTiO_3_-OV), and their derivatives (RuO_2_-NiO-BaTiO_3_) [132–134]. Moreover, Kisch et al. reported a kind of photocatalyst capable of reducing N_2_ to ammonium salt and nitrite under ambient conditions was obtained by adjusting the ratio of Fe, Ti, and O in materials derived from natural ilmenite (FeTiO_3_) [135]. Subsequently, multi-layered photoelectrodes incorporating LSPR metals with semiconductor-based materials have been prepared for efficient clean-energy-related conversion [136]. Based on theory and experiment, two studies reported successively by Misawa et al. demonstrated the composite photoelectrodes assembled with multi-layer semiconductor-based materials (Au-NPs/Nb-SrTiO_3_/Ru and Au-NPs/Nb-SrTiO_3_/Zr/ZrO_x_) [137, 138]. With the participation of Au-NPs, the fabricated titanate-like semiconductors and plasmonic-metal building blocks possessed the enhanced photoelectro-catalytic activity. Both works involve multilayer semiconductor-based materials (Au-NPs/Nb-SrTiO_3_). For the former study, Au-NPs and Ru are uniformly fixed on both sides of Nb-SrTiO_3_ semiconductor. The deposited Ru acted as a co-catalyst to reduce N_2_ containing saturated water to NH_3_ in the cathodic chamber (Fig. 19a-d). Briefly, through this semiconductor composite, where excited electrons induce nitrogen and proton reduction in the Nb-SrTiO_3_ conduction band, while trapped holes at the Au-NPs/Nb-SrTiO_3_ water interface efficiently oxidize hydroxyl ions and ethanol, with the chemical potential difference accelerating the reaction process (Fig. 19e). While for the later study, the side of the Nb-SrTiO_3_ semiconductor where Ru was originally deposited has now been replaced with a thin layer of Zr/ZrO_x_, which is used as a substitute for acquiring the better selectivity for nitrogen photoreduction (Fig. 19f-h). The promotion of photoelectrochemical device with efficient NH_3_ photosynthesis is realized with trapped holes oxidizing species at the Au-NPs/Nb-SrTiO_3_ interface and photogenerated electrons reducing N_2_ to NH_3_ at the Zr/ZrO_x_ surface (Fig. 19i, j). Results of these two experiments demonstrated that Zr/ZrO interface was more favorable to absorb N_2_ than that of Ru, with better N_2_ photo-fixation selectivity, also validated predictions in the previous studies simultaneously (Fig. 19k) [59].

**Fig. 20:**
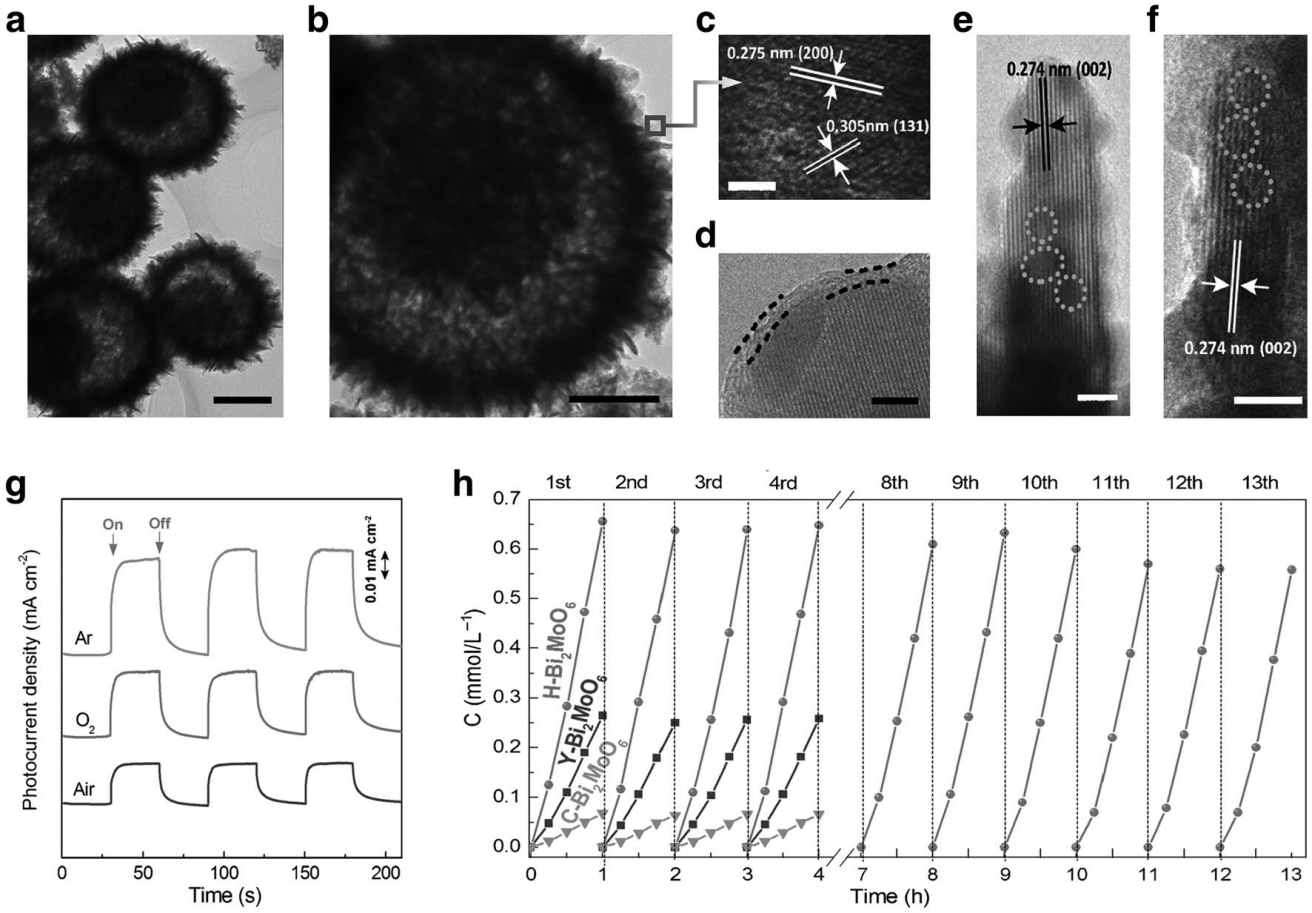
**a**, **b** TEM images, **c-f** HRTEM images of H-Bi_2_MoO_6_; the obvious disordered outer layer and lattice disorder induced by defects are marked by black parallel lines and grey circles. Scale bars: **a**, **b**, 200 nm, **c** 2 nm, **d-f** 5 nm. **g** Transient photocurrent responses of the H-Bi_2_MoO_6_ catalyst under different atmosphere. **h** Multicycle photocatalytic ammonia generation from air of different samples under simulated sunlight irradiation. Reproduced with permission [139]. Copyrights 2016 Wiley–VCH

In addition, another polyoxometalate-based material with excellent photocatalytic properties was also designed and fabricated to construct PEC system for nitrogen photo-fixation [139]. Through hydrothermal reaction, further calcination, and defect engineering, the modified hydrogenated bismuthmolybdate (H-Bi_2_MoO_6_) nanospheres with defective core–shell structure were obtained (Fig. 20a, b). The edge-exposed unsaturated Mo atom of Mo–O coordination polyhedron presented in the nanostructure was identified as the active centers of this catalyst (Fig. 20c-f). The unsaturated Mo can effectively adsorb, activate and reduce N_2_ molecule in combination with the occurrence of O defects. As expected, the photocurrent density of H-Bi_2_MoO_6_ in air is much lower than that in O_2_ and Ar, which confirms that the effective interface electron transfer between the catalyst and N_2_will not be blocked by surface carrier recombination and surrounding oxygen (Fig. 20g). As a nitrogen fixed photocatalyst, H-Bi_2_MoO_6_ has a reduction rate of up to 1.3 mmol g^–1^ h^–1^, which is 2.5 times and 9.5 times higher than that of traditional (Y-Bi_2_MoO_6_) and normal (C-Bi_2_MoO_6_) semiconductor catalysts. This is due to the optimized electronic structure and chemical adsorption sites, with a quantum efficiency of 0.73% at 500 nm and 0.25% at 600 nm (Fig. 20h).

Bismuth molybdate has been widely used in many fields as an environmentally friendly material with good stability, good dispersity, and good photocatalytic activity. Therefore, the modified bismuth molybdate-based catalysts need to be exploited and utilized for PEC nitrogen fixation.

**Fig. 21:**
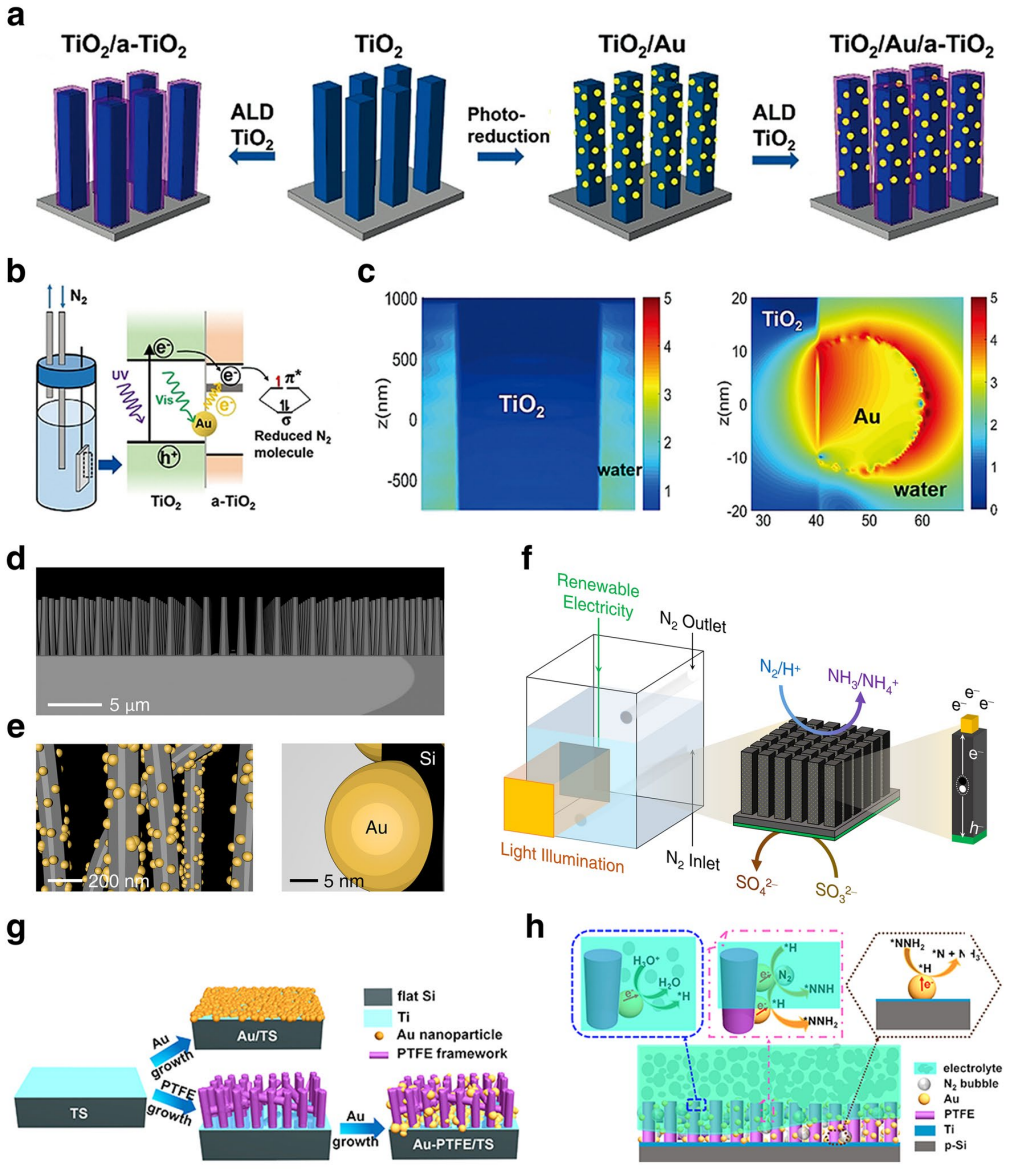
**a** Preparation process of TiO_2_/Au/a-TiO_2_ photoelectrodes. **b** Scheme of TiO_2_/Au/a-TiO_2_ for N_2_ photo-fixation. **c** FDTD simulations of electric field enhancement over TiO_2_ and TiO_2_/Au catalysts at 544 nm. Reproduced with permission [140]. Copyrights 2018 Wiley–VCH. **d, e** Scheme of GNPs-coated black silicon nanostructure. **f** Scheme of the cell. When the cell works, GNPs not only stabilize black silicon but also enhance light absorption. They act as electron trapping centers, promoting the effective separation of photogenerated electrons and holes, reducing the recombination of electron–hole pairs. The LSPR effect of GNPs aids in the direct bond breaking of absorbed N_2_ and its subsequent hydrogenation. **g** Scheme of the fabrication procedure of Au/TS and Au-PTFE/TS. **h** Scheme of NRR enhancement by introducing the aerophilic-hydrophilic hierarchical structure on Si-based photocathode. Reproduced with permission [143]. Copyright 2019 Elsevier

It is noteworthy that the tailored morphogenesis of semiconductor materials is designed to augment the adsorptive capacity of plasma-metal nanoparticles for gaseous species. Through photoreduction and atomic layer deposition, Gong et al. designed the plasmon-enhanced rutile TiO_2_ photoelectrode with rich superficial OVs (TiO_2_/Au/a-TiO_2_) as PEC catalyst for nitrogen fixation [140]. In this study, isolated Au NPs was anchored on the surface of rutile TiO_2_ (Fig. 21a). Meanwhile, OVs-enriched amorphous TiO_2_ film with precisely controlled thickness is conformally coated on the surface of Au NPs. This kind of ultrathin OVs-rich TiO_2_ film has contributed to the N_2_ adsorption, N_2_ activation, and better stability. The prospect of this kind of material lies in large-scale industrial production, and the NH_3_ yield can be further improved under the condition of applied bias (Fig. 21b, c). Moreover, Os as a common industrial catalyst for NH_3_ production or hydrogenation, was combined with Au NPs to study the catalytic activity of PEC nitrogen fixation under ambient conditions [141]. Due to the strong LSPR effect, Au NPs absorb the photo energy and activate N_2_. With the participation of Cs_2_O, the Os–Au nanohybrid can transform activated N_2_ into NH_3_ effortlessly. This strategy makes the noble metal Os free from the original high-temperature and high-pressure industrial catalytic environment, in line with the concept of a green way to acquire NH_3_.

As an important semiconductor, high-purity monocrystalline Si is widely used in many industries. Like a sponge that could absorb light, black silicon (bSi) is a new type of Si-based electronic semiconductor material that has been found in recent research to dramatically increase the efficiency of photoelectric conversion. It can capture almost all the sunlight (from visible light to infrared), improve the efficiency of light utilization, and generate hundreds of times the current density of traditional silicon materials. With the utilization of this gold nanoparticles (GNP), MacFarlane et al. prepared a nanostructured composite photoelectrode which was abbreviated as GNP/bSi/Cr [142]. As illustrated, the bSi wafer with nanorod arrays vertically grown on the upward side was uniformly coated with GNPs (Fig. 21d, e). When light illuminated on the electrode, on the upward side, the absorbed photoenergy was effectively converted into photocurrent to achieve NRR in the presence of GNPs. On the downward side, the layer-coated Cr acted as a sacrificial anode was used to gather holes (Fig. 21f). Because of the superior efficiency of photoenergy conversion, the photocatalytic system containing this kind of photocatalyst has two orders of magnitude higher NH_3_ yield rate than other systems containing plasmonic metals under the same working time. This considerable yield makes this catalytic system potentially useful for large-scale production. In addition, based on the creative modification of Si wafer, an aerophilic-hydrophilic hetero-structured photoelectrode was fabricated for PEC nitrogen fixation by Wang and co-workers [143]. This well-designed heterostructure semiconductor-based catalyst consists of three main parts: (i) a high-purity Si plate with a thin Ti film composite of its surface; (ii) a hydrophobic porous framework composed of polytetrafluoroethylene (PTFE); and (iii) a certain amount of well-dispersed and free-stacked Au NPs coated on the polymer framework (Fig. 21g). Each component plays an important role in the photocatalytic system, making these ingenious designs include the following advantages: (i) the passivated substrate formed a composite of TiO_2_ and Si, which can effectively absorb the optical energy and assist the PEC catalysis; (ii) the passivated interface was beneficial to the fixation of PTFE and the growth of Au NPs; (iii) PTFE has achieved good hydrophobicity and high chemical stability as the photoelectrode assembly, which were beneficial to the N_2_ diffusion, HER suppression, and long-term photo-fixation; and (iv) the superior NRR photocatalytic abilities from the well-dispersed Au NPs. As a highlight of this research, designing photoelectrode with this cheap, convenient, non-toxic and stable polymeric porous framework is an invaluable idea of great innovation (Fig. 21h). This may orient the design of PEC NRR system through a new pathway.

#### Conducting Metal Sulfides

Some other composites have improved their photocatalytic NRR properties by compounding with metal sulfides. For example, the hierarchical composite CdS/Pt/RuO_2_ was used to catalyze NRR under visible-light illumination under ambient conditions [115]. Pt improved the conductivity, with the assistance of CdS, N_2_ was activated and converted into Ru-N_2_ complexes. Similarly, the modified semiconductor-based composite with two introduced sulfides (CdS and Ag_2_S) further improved the NRR photocatalytic performance [116]. The combination of Ag_2_S and RuO_2_ facilitates the electron migration from CB to the potential of minimized CdS photo-corrosion. The limitation of these two works is that complexes containing noble metals are used in aqueous solution, which is unfavorable in cost and NH_3_-collection. In addition to the assembly of inorganic materials, there are also attempts to biomaterials.

#### Conducting Biomaterials

**Fig. 22:**
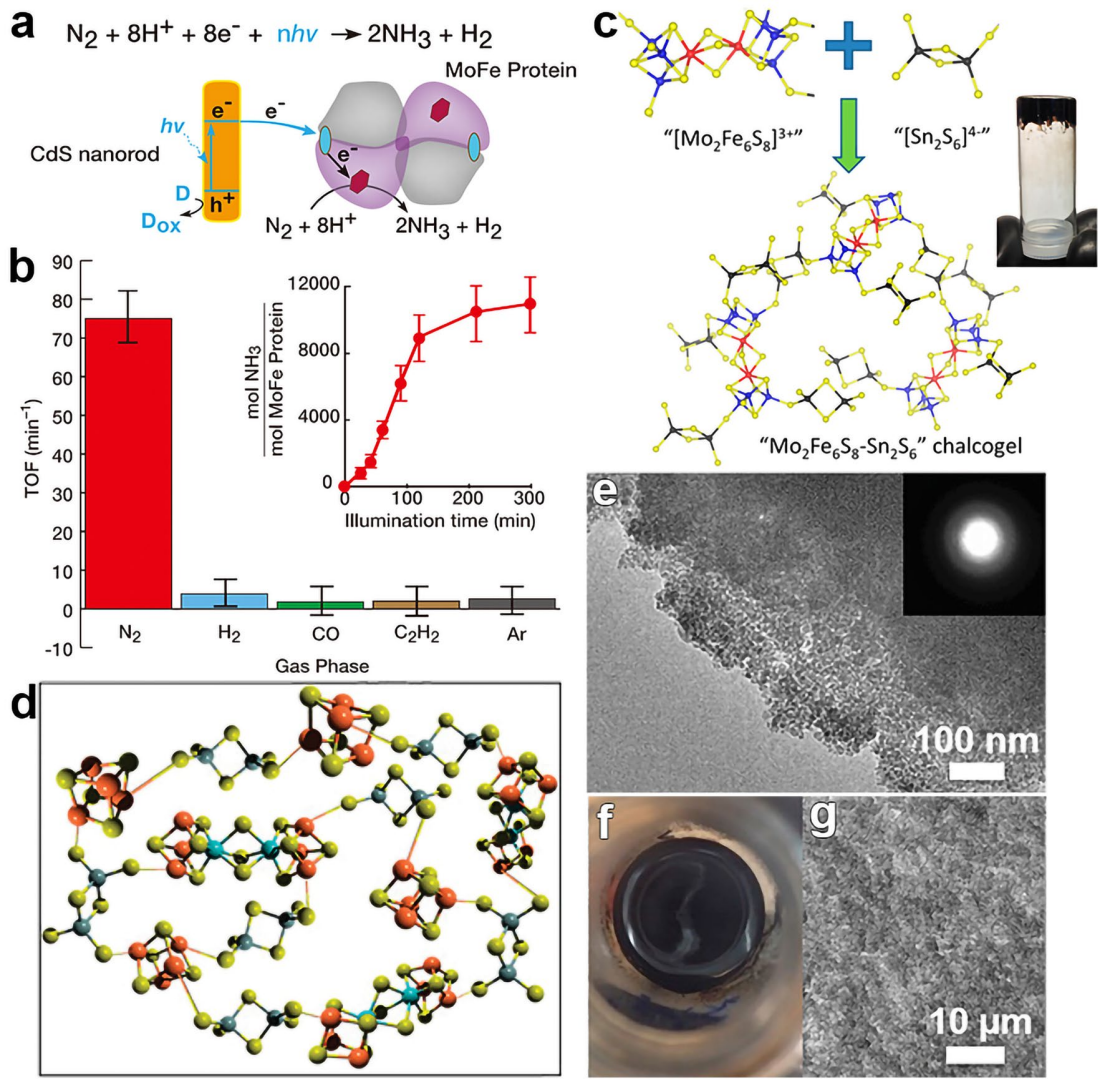
**a** Reaction catalyzed by CdS: MoFe protein biohybrids. **b** TOF of catalytic reduction of N_2_ to NH_3_ was measured under different gas configurations and controls. Reproduced with permission [117]. Copyright 2016 AAAS. **c** Schematic representation of Mo_2_Fe_6_S_8_ − Sn_2_S_6_ chalcogel, building block scheme (Mo, blue; Fe, red; S, yellow; Sn, black), and a complete chalcogel shown at right. Reproduced with permission [118]. Copyright 2015 ACS. **d** Chalcogel network consisting of “Mo_2_Fe_6_S_8_(SPh)_3_” and “Fe_4_S_4_” cores linked with Sn_2_S_6_^4−^ clusters. **e** TEM images of FeMoS–FeS–SnS chalcogel with corresponding SAED (inset e). **f** Photo of black FeMoS–FeS–SnS chalcogel. **g** SEM of FeMoS–FeS–SnS chalcogel

The combination of CdS and biomaterials have been applied in other clean energy conversion [199–202]. Meanwhile, the research on semi-artificial nitrogen photo-fixation using this type of technique was firstly reported by King et al. [117]. Conventional biological nitrogen fixation is accomplished by nitrogenase. The newly constructed photocatalytic system chose CdS nanorod as a substitute for Fe protein, and switched energy supply from ATP to photon energy (Fig. 22a, b). Herein, the morphology-optimized semiconductor material acts as an energy converter. The supplied energy allows the biomaterial to complete the subsequent complex bond cleavage process. The turn over frequency (TOF) of this technology is 75 (min^−1^), 63% of the ATP-coupled reaction rate for the nitrogenase under optimal conditions. Similarly, inspired by advanced MoFe-cofactor mechanism in nitrogenase, biomimetic amorphous network combined Fe_2_Mo_6_S_8_ chalcogel with Sn_2_S_6_ ligands as photocatalyst effectively converted N_2_ to NH_3_ mildly (Fig. 22c) [118]. Moreover, the modified two-component biomimetic clusters consisted of Fe_2_Mo_6_S_8_ (SPh)_3_ and Fe_3_S_4_ were linked by Sn_2_S_6_ to form an emerging chalcogel (Fig. 22d-g) [119]. The results of the theoretical model analysis show that Fe played a more important role in photocatalytic NRR [118]. Therefore, the developed nitrogenase analogues after understanding the reaction mechanism could have wider applications and better catalytic performances than nitrogenase in nature.

#### Conducting Carbonaceous Materials

The most widely used carbon-based material with photoresponse is g-C_3_N_4_. As a typical polymer semiconductor, C, N atoms in the structure are sp2 hybridized to form a highly delocalized π-conjugated system. Compared with traditional metal-based semiconductors represented by nanoscale TiO_2_, g-C_3_N_4_ has many advantages: innocuity, harmlessness, wider light response, and environmental friendliness. The assembly of CdS-derived ternary sulfides with g-C_3_N_4_ (ZnSnCdS/g-C_3_N_4_ and ZnMoCdS/g-C_3_N_4_) have been reported [120, 203]. The common advantages of these two photocatalysts were included as follows: (i) the efficient charge transfer due to the tight coupling between g-C_3_N_4_ and metal sulfides; (ii) the smooth transfer of photogenerated carriers back and forth in the structure; (iii) the improved absorption of visible light; and (iv) the establishment of internal electric field originated from the rearrangement of carriers in heterojunction.

Notably, direct Z-scheme semiconductor-based catalysts are considering to be more effective for photocatalytic NRR performance. Since Z-scheme catalyst can effectively inhibit the combination of electrons and holes, the redox ability of the catalyst can be guaranteed [121]. Based on this newly-developed photocatalyst type, many g-C_3_N_4_-based composites have emerged rapidly: (i) the MgAlFeO/g-C_3_N_4_ nanorod was in situ formed by hydrothermal reaction with atomic-ratio tuning [122]. Followed with the charge-carrier separation mechanism, nitrogen photo-fixation was realized; (ii) similar heterojunction photocatalyst W_18_O_49_/g-C_3_N_4_ was proposed to convert N_2_ to NH_3_ under full-spectrum illumination from UV to near-infrared region [123]. The introduced OVs induced the coherent oscillation of free electrons on the surface; (iii) the organo-functionalized all-solid-state Ga_2_O_3_-DBD/g-C_3_N_4_ was applied to convert N_2_ to NH_3_ through aromatic rings under ambient conditions [124]. The superior catalytic performance was benefited from the wide-range light absorption, high redox potential, and high charge-separation efficiency. The ·CO_2_^–^ formed by methanol participation in the oxidation reaction is the major species promoting the reduction of N_2_.

**Fig. 23:**
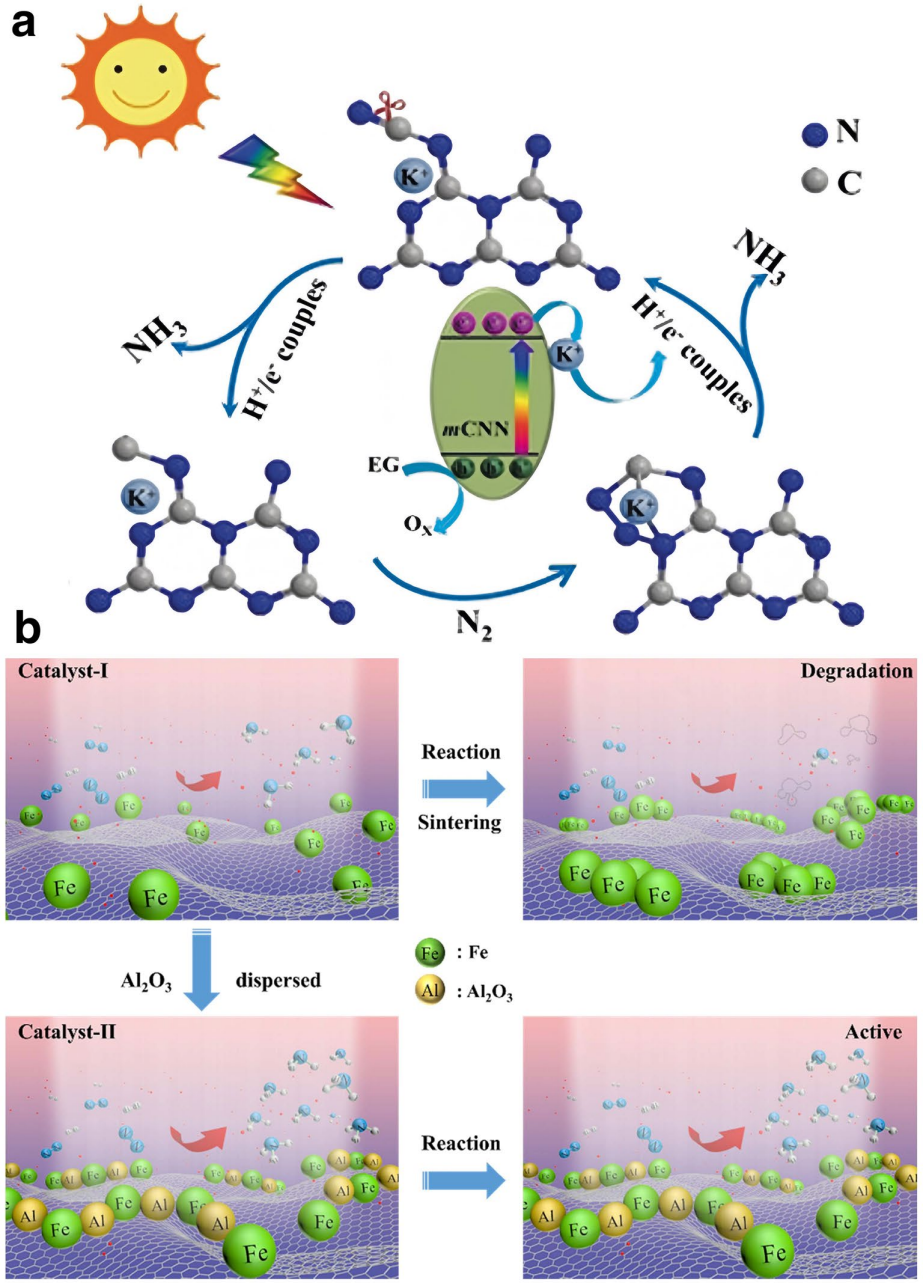
**a** Schematic illustration of NRR pathway using the *m*CNN photocatalyst with K^+^-assisted –C≡N regeneration. Reproduced with permission [125]. Copyright 2019 Wiley–VCH. **b** Schematic diagram of ammonia synthesis on Fe@3DG and Fe-Al@3DG. Reproduced with permission [127]. Copyright 2017 Elsevier

Moreover, g-C_3_N_4_ can also be modified with organic groups or metal cations to improve the photocatalytic NRR performance. In the work reported by Zhao et al., cyano groups and potassium cation were selected to modify the g-C_3_N_4_ nanoribbons (*m*CNN) [125]. Studies have shown that –C≡N in *m*CNN can be regenerated by means of embedded potassium cation via a pathway similar to the Mars van Krevelen process. As a result, it was confirmed that the regeneration of the cyano group enhanced the photocatalytic activity, maintained the catalytic cycle, and stabilized the photocatalyst (Fig. 23a). By introducing a new C–N bond as an intermediate in N_2_ to NH_3_ reaction and using metal cations to enhance N_2_ adsorption is worth learning. The conducting substrate rGO also has been considered in designing low-cost photocatalysts. The simple and reliable 2D rGO/g-C_3_N_4_ heterostructure hybrid catalyst was possessed to use in photo-fixation of N_2_ [126]. The NH_3_ yield rate monitored from aqueous samples with protonated g-C_3_N_4_ is four times higher than that of the original g-C_3_N_4_-based nanohybrid. Further research demonstrated that the region of face-to-face contact between g-C_3_N_4_ and rGO can significantly reduce the recombination of photogenerated carrier and improve the NRR performance. In general, g-C_3_N_4_/graphene-based hybrid semiconductor catalysts are appealing because of their competitive NRR catalytic activities, dramatically reduced cost, and outstanding stability, which make them viable alternatives to replace the conventional photocatalytic NRR catalysts [127]. For nitrogen photo-fixation, 3D self-assembly materials should also be developed on the basis of 2D/2D or 2D/0D composite materials with excellent catalytic properties. For example, Chen et al. reported that cross-linked 3D graphene combined with active metal materials can obtain excellent performance for photo-fixation [127, 128]. Catalysts with singular-existed iron-based semiconductor indicated a poor stability (Fe@3DG). With the evolution of design, the reintroduced semiconductor material Al_2_O_3_ formed by adding Al(NO_3_)_3_ plays a critical role in assisting dispersion and preventing aggregation of metal NPs in the catalyst (Fe-Al@3DG), which greatly improves the photocatalytic NRR performance of the binary metal–carbon composite (Fig. 23b). Hence some other clean-energy-related work is worth referring to [204–209].

### Heterostructure Creating

**Fig. 24:**
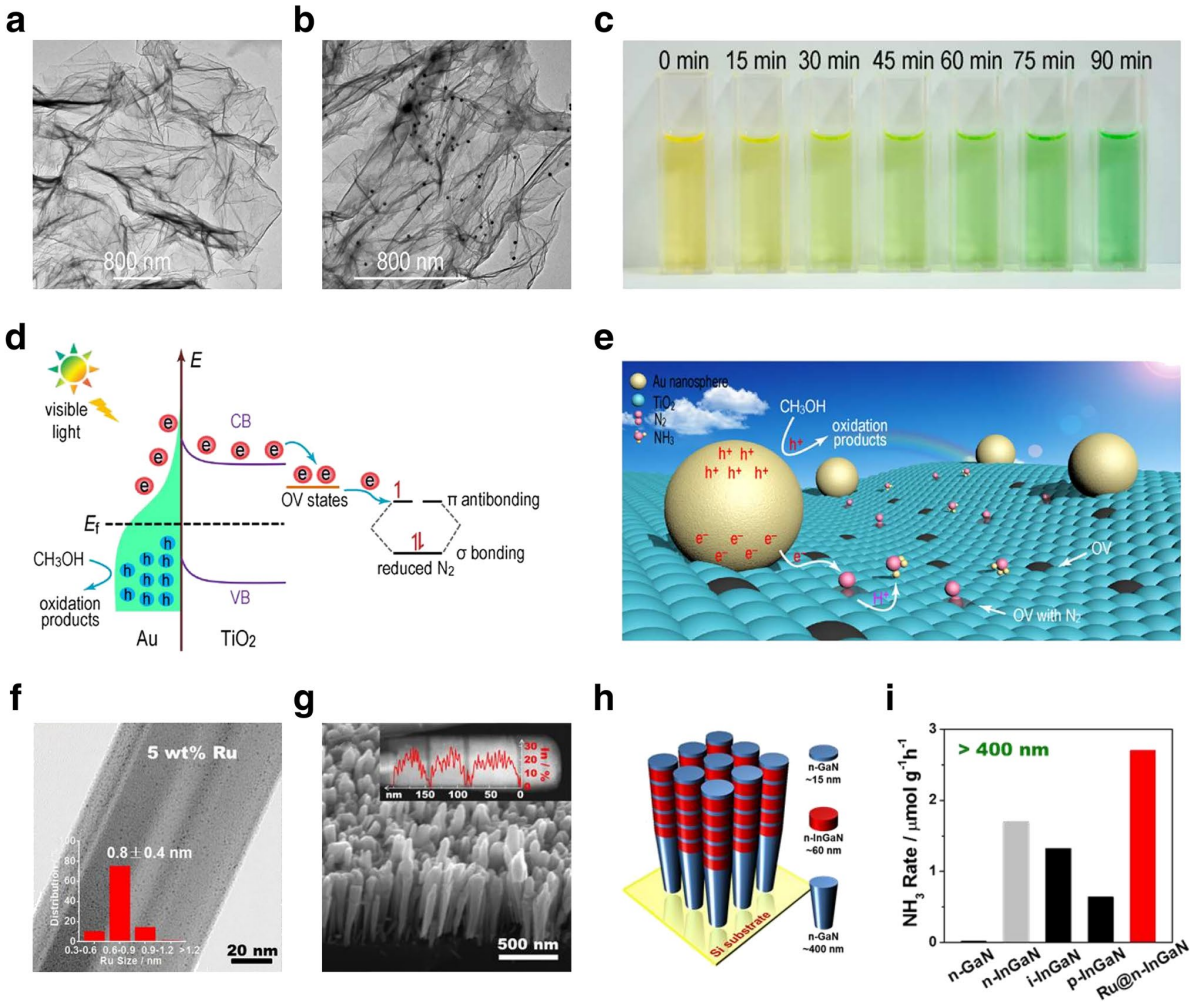
**a**, **b** SEM/TEM of Au/TiO_2_-OV. **c** Photograph of indophenol-blue test. **d** Schematic illustration of the plasmonic hot electron generation, injection, N_2_ reduction processes under visible light. **e** Artistic illustration of the efficient plasmonic N_2_ photo-fixation. Reproduced with permission [129]. Copyright 2018 ACS. **f** TEM of the 5 wt% Ru-modified GaN NWs.** g** SEM of the InGaN/GaN nanowires on Si (111) substrate. **h** Schematic of the InGaN/GaN nanowire structure. **i** Rate of NH_3_ generation over various III-nitride semiconductors under visible light. Reproduced with permission [130]. Copyrights 2017 Wiley–VCH

Two or more design strategies have been used in some studies for the construction of semiconductor-based catalysts. A typical work reported by Yu et al. demonstrates the Au anchored OVs-rich TiO_2_ NSs (Au/TiO_2_-OV) with improved performance could achieve efficient photocatalytic NRR under ambient conditions [129]. Noble metal doping and defect construction are combined ingeniously. Au NPs can be evenly distributed on the TiO_2_-OVs NSs. (Fig. 24a, b). The high NH_3_ yield rate was competitive and could be visually represented by chromogenic reaction (Fig. 24c). The “working-in-tandem” pathway included three steps complete the processes of adsorption, activation, and conversion in sequence. OVs act as the activation sites of N_2_ molecules on the surface of the material. Based on the LSPR effect of Au NPs under illumination, the generated plasmonic hot electrons can further convert activated N_2_ molecules into NH_3_ (Fig. 24d, e). Recently, Li et al. reported a composite semiconductor-based photocatalyst with rational design [130]. The one-dimensional n-scheme GaN (n-GaN) based semiconductor nanowires were grown on a Si (111) substrate using radio frequency plasma-assisted molecular beam epitaxy technique in N_2_ atmosphere. The ultrasmall Ru co-catalysts were deposited on the surface of nanowires to form the final catalyst (5 wt% Ru@n-InGaN) (Fig. 24f-h). As a contrast, with the doping of In, the bandgap of the material was shifted to the visible light region, which enhance the photo utilization. Moreover, when Ru was added, the Schottky junction constructed on the surface of the material can promote the transfer of photogenerated electrons from nitrides to Ru clusters. The cleavage procedure of N≡N bond was further facilitated. The double doping strategy not only improves the photoresponse performance of the semiconductor substrate, but also improves the catalytic ability of the overall material to activate and reduce N_2_ (Fig. 24i). Generally speaking, the combination of design strategies can effectively solve the N_2_ activation and reduction as two key obstacles in photocatalytic nitrogen fixation and broaden the application scope of semiconductor-based catalysts. Moreover, this strategy is continuously evolving [210].

## Conclusions and Perspectives

From a multifaceted analytical standpoint, the conversion and sequestration of clean energy through the auspices of mild photocatalytic nitrogen fixation is accorded a position of strategic eminence over the singular hydrogen economy paradigm. Ammonia and nitric acid, as quintessential feedstocks, command the foremost priority in the synthesis hierarchy for the production of nitrogenous fertilizers and chemical commodities. In the prospective hydrogen economy, liquid ammonia emerges as the preeminent solution to the logistical challenges inherent in hydrogen transportation. This comprehensive review meticulously encapsulates the advancements in the design of photocatalytic nitrogen fixation systems and the pivotal transition from experimental to industrial scales. The domains that warrant fervent scholarly attention are delineated as follows: (i) the quest for alternative catalysts to noble and rare earth metals or the enhancement of their catalytic efficiency; (ii) the architectural innovation of semiconductor-based catalysts, encompassing their structural, compositional, and morphological optimization, alongside the refinement of ancillary components within the photocatalytic matrix; (iii) the incisive development of innovative, efficacious, and eco-friendly nitrogen fixation methodologies that aspire to rival the hegemony of the Haber–Bosch and Ostwald processes, aligning with the exigencies of future demands. Despite the commendable progress in augmenting the photocatalytic NRR and NOR efficiencies, the trajectory of research and innovation remains unflagging. As we project into the horizon of this burgeoning field, it is imperative to concurrently address the inevitable challenges that accompany its evolution. Consequently, several prescriptive recommendations are proffered for consideration. It is aspired that, with the holistic refinement of these dimensions, the lofty goal of achieving green nitrogen fixation under ambient conditions can be realized in the forthcoming epoch. Appended herewith, Table 1 provides a compendium of the catalytic performance metrics reported within the selected literature corpus.

Firstly, as one of the four molecules that are abundant in nature and of significant interest for human utilization (H_2_O, CO_2_, N_2_, CH_4_), pinpointing the sources of inefficiency is of paramount importance. Extensive research has delineated a hierarchy of feasibility: H_2_O is significantly more favorable than CO_2_, with N_2_ and CH_4_ trailing behind. The mechanism of the hydrogen evolution reaction (H_2_ORR) has been largely elucidated through years of dedicated research. In contrast, the mechanisms of NRR and NOR, remain in their nascent stages of understanding. The NRR and NOR processes are more complex due to the higher number of protons and electrons involved, the more challenging adsorption of N_2_, and the more stringent conditions required to cleave the N≡N triple bond. Consequently, these processes are inherently more intricate than the hydrogen or carbon dioxide reduction reactions, with less predictable catalytic interfaces, making their realization significantly more challenging.

In numerous studies on the NRR mechanism, analogous steps to the carbon dioxide reduction reaction (CO_2_RR) have been employed for elucidation. However, the bond energy of the C=O bond does not compare to the strength of the N≡N bond, rendering many theoretically derived materials ineffective for the adsorption and activation of N_2_. The exploration of the NOR mechanism is even less developed. Most of the identified direct mild nitrogen fixation catalysts, particularly those composite materials based on semiconductors, lack in-depth investigation at the nanoscale regarding the functionality of each constituent component. Therefore, there is an imperative need for the integration of advanced real-time detection methods with sophisticated theoretical calculations to further elucidate the photocatalytic mechanisms of NRR and NOR.

Secondly, the standardization of experimental protocols and the validation of activity for artificial mild photo-fixation of nitrogen systems are essential for material screening and scale-up. In the foreseeable future, as the cornerstone of the burgeoning energy economy, catalysts must undergo scientific comparison not only of their catalytic performance but also of the estimated costs associated with their catalytic systems. Although normalization methods can facilitate the comparison of experimental data within a single study, direct comparison of the various NRR or NOR catalytic performances obtained through different technologies remains a formidable challenge. Additionally, there is a dearth of simple and efficient characterization methods for the photoreaction process and high-throughput detection technologies for products. It is advocated that the technologies discussed in this review be applied to research and mass production with the aim of developing industrial standard components. Cost accounting is crucial for the further development of this energy economy. The application of photocatalytic nitrogen fixation is currently constrained by its low efficiency. Fortunately, the supply of light energy is entirely clean and carbon–neutral, with costs incurred solely by the catalytic device, thereby eliminating losses in energy conversion. However, current systems are characterized by high costs and low efficiency, indicating a long developmental trajectory ahead. Therefore, it is imperative to provide as much information as possible regarding the catalytic system to facilitate researchers' ability to assess the optimal scheme. In this regard, standardization policies for clean energy conversion are worthy of emulation. The guidelines proposed by Chorkendorff and colleagues in the field of electrocatalytic NRR are particularly instructive for the field of photocatalysis. These guidelines will provide researchers with a framework to further test and explore the true catalytic performance of catalysts and the reliability of the final products, including a series of methods to exclude false positive errors [211–213]. In the synthesis of catalysts, nitrogen-containing compounds are often introduced, which, if not removed prior to colorimetric detection, can lead to false positive errors. The low efficiency of photocatalytic nitrogen fixation results in low NH_3_ yields, and the failure to eliminate such interference can mislead researchers. Fortunately, relevant studies have been reported, identifying all potential interfering substances and emphasizing their impact on each quantification method [214]. The influence of components within the device on experimental outcomes should also be considered [215].

Currently, achieving a satisfactory balance between efficiency and yield in nitrogen fixation under mild conditions remains a formidable challenge. The primary issue to overcome is the low efficiency, with most NRR reverse fuel cells operating at room temperature exhibiting efficiencies in the range of 1% to 15%. This low efficiency inevitably leads to unsatisfactory yields. Furthermore, it is essential to discuss catalytic systems that exhibit high efficiency or high yield. For instance, pioneering studies by the MacFarlane team have increased the Faradaic efficiency (FE) to 70%, albeit at the expense of a slow proton mass transfer process that significantly reduces the production rate of these systems. Subsequent improvements have accelerated NH_3_ production, yet they still do not meet the requirements set by the U.S. Department of Energy (DOE). Another project led by O'Hayre and collaborators is developing a novel button-sized reverse fuel cell made from high-temperature ceramic materials [216, 217]. Although this catalytic system has achieved the highest record for NH_3_ production rate, the Faradaic efficiency is unsatisfactory, and the NH_3_ yield per unit remains too low. The overall production rate of this technology is reportedly 70 times lower than the DOE's target. This underscores the reliance on the exploration of new materials, which is key to addressing fundamental issues. The development of new materials must still strive to achieve several constant objectives: (i) enhancing the basic photocatalytic NRR and NOR performance; (ii) improving long-term stability; (iii) enhancing environmental adaptability and broadening the scope of application; and (iv) reducing the cost of constructing catalytic devices. Although it is challenging to obtain materials that possess all of these advantages simultaneously, the mass production of catalysts can be advanced through a comprehensive consideration of alternative solutions. In general, enhancing the performance of the catalytic system requires two contributions: (i) the selection of catalysts with superior performance; (ii) the continuous optimization of other components. Additionally, new catalytic materials and potential novel catalytic methods must be considered. Despite the challenges of achieving environmentally friendly and economical large-scale mild photo-fixation, efforts must be made to find the right direction to balance efficiency and rate, ultimately promoting the development of the ammonia economy [218].

Thirdly, it is necessary to comprehensively assess the current status of photocatalytic nitrogen fixation from multiple aspects. The economic and environmental impacts of photocatalytic nitrogen fixation techniques are pivotal to their potential adoption and scalability. Economically, these techniques offer a promising alternative to the energy-intensive H-B process by operating under ambient conditions, thus avoiding high-pressure and high-temperature infrastructure requirements. This can lead to significant reductions in operational costs, particularly when sunlight serves as the primary energy source. However, the cost-effectiveness is currently limited by the need for high-performance photocatalysts, which often rely on rare or expensive materials such as noble metals. Advances in catalyst design, including the use of abundant elements, single-atom catalysts, and defect engineering, aim to reduce these material costs. Environmentally, photocatalytic nitrogen fixation has the potential to minimize greenhouse gas emissions by eliminating fossil fuel-derived hydrogen and leveraging solar energy, a renewable resource. Furthermore, these processes can reduce environmental degradation associated with ammonia production, as they avoid the CO_2_-intensive pathways of traditional methods. However, concerns remain about the lifecycle impacts of photocatalysts, particularly regarding their synthesis, stability, and disposal, which may involve energy—intensive processes or toxic by-products. A comprehensive lifecycle assessment (LCA) of photocatalyst materials and systems is crucial to fully realize their environmental benefits while ensuring their economic viability for widespread adoption.

It is gratifying to note that the productivities of mild photo-fixation processes have gradually increased. Through ongoing endeavor, the effect of nitrogen fixation under ambient condition is making progress on the established goal [219]. With the attempts of industrial nitrogen fixation under ambient condition by some research groups through various means, the development of this direction is getting better. Objectively speaking, the nitrogen photo-fixation under ambient condition is still very difficult. However, the future of green ammonia industry is indisputably promising and prospective with the synergetic collaborations of practitioners from various disciplines.

The continuous advancement of these technologies has provided novel insights and methodologies for the research of photocatalytic nitrogen fixation. Moving forward, we will focus on discussing key research areas that warrant the attention of the academic community. Emerging technologies, notably artificial intelligence (AI) and machine learning (ML), have the potential to transform photocatalytic nitrogen fixation by expediting catalyst design, discovery, and optimization. AI-driven algorithms can analyze extensive datasets from experimental and computational studies, facilitating the swift identification of correlations between material properties and catalytic performance. Machine learning models can predict catalyst activity and stability based on features such as electronic structure, bandgap, and surface properties, significantly reducing reliance on trial-and-error experimentation. Moreover, AI-powered generative models can propose novel catalyst designs, suggesting compositions or structural modifications tailored for nitrogen adsorption, activation, and reduction. Beyond catalyst discovery, AI and ML can optimize reaction conditions by analyzing data from in situ and operando measurements, providing insights into the effects of temperature, light intensity, and reactant concentrations on photocatalytic performance. These tools can also aid in the rational design of heterostructures, dopants, and defect engineering strategies by pinpointing the most promising combinations for enhanced charge separation and nitrogen activation. Furthermore, the integration of AI with high-throughput screening platforms and robotic synthesis systems enables the efficient exploration of multidimensional parameter spaces, significantly shortening the time from concept to application. As these technologies evolve, they are poised to address challenges in selectivity, efficiency, and scalability, bringing photocatalytic nitrogen fixation closer to practical application. By combining computational insights with experimental validation, AI and ML offer a paradigm shift in designing environmentally sustainable and economically viable nitrogen fixation systems.

Overall, we strongly believe that with the enrichment of photocatalytic nitrogen fixation systems, a subversive revolution of clean energy with economic benefits and environmental protection will coming soon. With the ongoing endeavors of multiple scientific subfields, the future photocatalytic efficiency and productivity will undoubtedly surpass the contents discussed in this article. As a result of scientific and technological progress and the crystallization of human wisdom, green ammonia energy will grow to dominate the new energy market in the future.
